# Oxaphospholes and Bisphospholes from Phosphinophosphonates and α,β-Unsaturated Ketones

**DOI:** 10.1002/chem.201302014

**Published:** 2013-08-26

**Authors:** Anna I Arkhypchuk, Andreas Orthaber, Viorica Alina Mihali, Andreas Ehlers, Koop Lammertsma, Sascha Ott

**Affiliations:** [a] Department of Chemistry, Ångström Laboratories, Uppsala University, Box 523, 751 20 Uppsala (Sweden); [b] Department of Chemistry and Pharmacochemistry, VU University Amsterdam, de Boelelaan 1083, 1081 HV Amsterdam (The Netherlands)

**Keywords:** cumulenes, density functional calculations, domino reactions, phosphaorganic chemistry, reaction mechanisms

## Abstract

The reaction of a {W(CO)_5_}-stabilized phosphinophosphonate **1**, (CO)_5_WPH(Ph)–P(O)(OEt)_2_, with ethynyl- (**2 a**–**f**) and diethynylketones (**7**–**11**, **18**, and **19**) in the presence of lithium diisopropylamide (LDA) is examined. Lithiated **1** undergoes nucleophilic attack in the Michael position of the acetylenic ketones, as long as this position is not sterically encumbered by bulky (*i*Pr)_3_Si substituents. Reaction of all other monoacetylenic ketones with lithiated **1** results in the formation of 2,5-dihydro-1,2-oxaphospholes **3** and **4**. When diacetylenic ketones are employed in the reaction, two very different product types can be isolated. If at least one (Me)_3_Si or (Et)_3_Si acetylene terminus is present, as in **7**, **8**, and **19**, an anionic oxaphosphole intermediate can react further with a second equivalent of ketone to give cumulene-decorated oxaphospholes **14**, **15**, **24**, and **25**. Diacetylenic ketones **10** and **11**, with two aromatic acetylene substituents, react with lithitated **1** to form exclusively ethenyl-bridged bisphospholes **16** and **17**. Mechanisms that rationalize the formation of all heterocycles are presented and are supported by DFT calculations. Computational studies suggest that thermodynamic, as well as kinetic, considerations dictate the observed reactivity. The calculated reaction pathways reveal a number of almost isoenergetic intermediates that follow after ring opening of the initially formed oxadiphosphetane. Bisphosphole formation through a carbene intermediate **G** is greatly favored in the presence of phenyl substituents, whereas the formation of cumulene-decorated oxaphospholes is more exothermic for the trimethylsilyl-containing substrates. The pathway to the latter compounds contains a 1,3-shift of the group that stems from the acetylene terminus of the ketone substrates. For silyl substituents, the 1,3-shift proceeds along a smooth potential energy surface through a transition state that is characterized by a pentacoordinated silicon center. In contrast, a high-lying transition state **TS(E′–F′)_R=Ph_** of 37 kcal mol^−1^ is found when the substituent is a phenyl group, thus explaining the experimental observation that aryl-terminated diethynylketones **10** and **11** exclusively form bisphospholes **16** and **17**.

## Introduction

Known for 60 years through the pioneering work of Wittig and Geissler,^1^ phospholes have experienced a remarkable renaissance over the last decade. The interest is fuelled by the tetrahedral nature of the phosphorous center and the resulting low aromaticity of the heterocycle. Phosphole’s low aromaticity is reflected in a relatively modest “nucleus-independent chemical shift” (NICS) value of −5.3 compared to that of its N-analogue, that is, pyrrole (NICS=−15.1).^2^ As the P center remains outside the π-conjugation paths, it allows fine tuning of the optical properties of the whole system by chemical modifications such as metal coordination, oxidation, or addition of electrophiles (formation of phosphonium salts) on the heteroatom.^3^–^6^ The electronic impact that the chemically modified P center has on the adjacent π system has been coined the “doping effect”.^4^, ^7^ Annelation of (hetero)aromatic ring systems enables additional ways to modify the electronics of phospholes.^8^ The possibility to tune their electronic properties, and thus their absorption and emission behavior over a large range makes phospholes targets for applications in organic electronic devices, such as OLEDs,^9^ and as dyes within organic solar cells.^10^

From a synthetic viewpoint, early routes to phospholes were typically based on reactions between dilithium salts and dichlorophosphines^11^ or the addition of phosphines to acetylenes, followed by formation of the heterocycle.^12^, ^13^ Most of these procedures suffer from restrictions regarding the substrate structure and do not allow large variation of the substituents on the final product.

The development of the so-called Fagan–Nugent synthetic strategy in 1988 gave new life to phosphole chemistry.^14^ This methodology, based on treating acetylenes with an activated zirconocene complex followed by quenching of the newly formed zirconium metallacycle by phosphorus dichloride, allowed the preparation of large libraries of target structures.^15^ The biggest drawback of the method, the low selectivity when asymmetrically substituted acetylenes are used, was overcome by simply linking the two reacting acetylenes through an inert bridge. This modification resulted in not only increased selectivity, but also improved yields.^16^ The versatility and elegance of the method made it possible to introduce a large variety of substituents at the 2- and 5-positions of the phosphole ring and at the phosphorus center.^9^, ^17^

Yet, despite the flexibility of the Fagan–Nugent method, it is not suitable for preparing heterophospholes like oxaphospholes,^18^, ^19^ which represent one of the biggest classes of phosphorus-containing molecules. Recently, benzannelated 1,3-oxaphospholes drew much attention due to their intriguing luminescent properties.^20^ 1,2-Oxaphospholes have been addressed in a few instances, but remain an almost unexplored type of phosphorus-containing heterocycle due to the absence of reliable synthetic approaches.^21^

Herein, we present a synthetic strategy to convert readily available phosphinophosphonates and ethynylketones into 1,2-oxaphospholes. The prepared 1,2-oxaphospholes are highly substituted and thus offer the possibility for rich follow-up chemistry. Furthermore, it is shown that introduction of a second acetylene into the ketone substrate initiates subsequent chemistry and leads to the formation of oxaphospholes with appended cumulene frameworks or conjugated bisphospholes. The reaction outcome is determined by the substituents at the remote acetylene termini. We show that these reactions are of general applicability and can be used for the preparation of a variety of oxaphospholes and bisphospholes. The reaction sequences are followed by in situ FTIR spectroscopy using the stretching vibrations of the tungsten-coordinated CO ligands as sensitive reporters. Reaction mechanisms that rationalize all transformations are proposed and key steps are supported by DFT calculations.

## Results and Discussion

Phosphinophosphonates are well-established reagents in the so-called phospha-Wittig–Horner reaction,^22^ that is, the *P* analogue of the Horner–Wadsworth–Emmons reaction.^23^ As such, they convert simple aldehydes and ketones to the corresponding phosphaalkenes. Stabilization of phosphinophosphonates, as well as phosphaalkene products, is ensured either by bulky substituents at P^III^ or the coordination of metal fragments to the same center. The latter strategy allows for a larger substrate scope at P^III^ and therefore a {W(CO)_5_}-coordinated phosphinophosphonate **1** was chosen for this study.

**Scheme 1:**
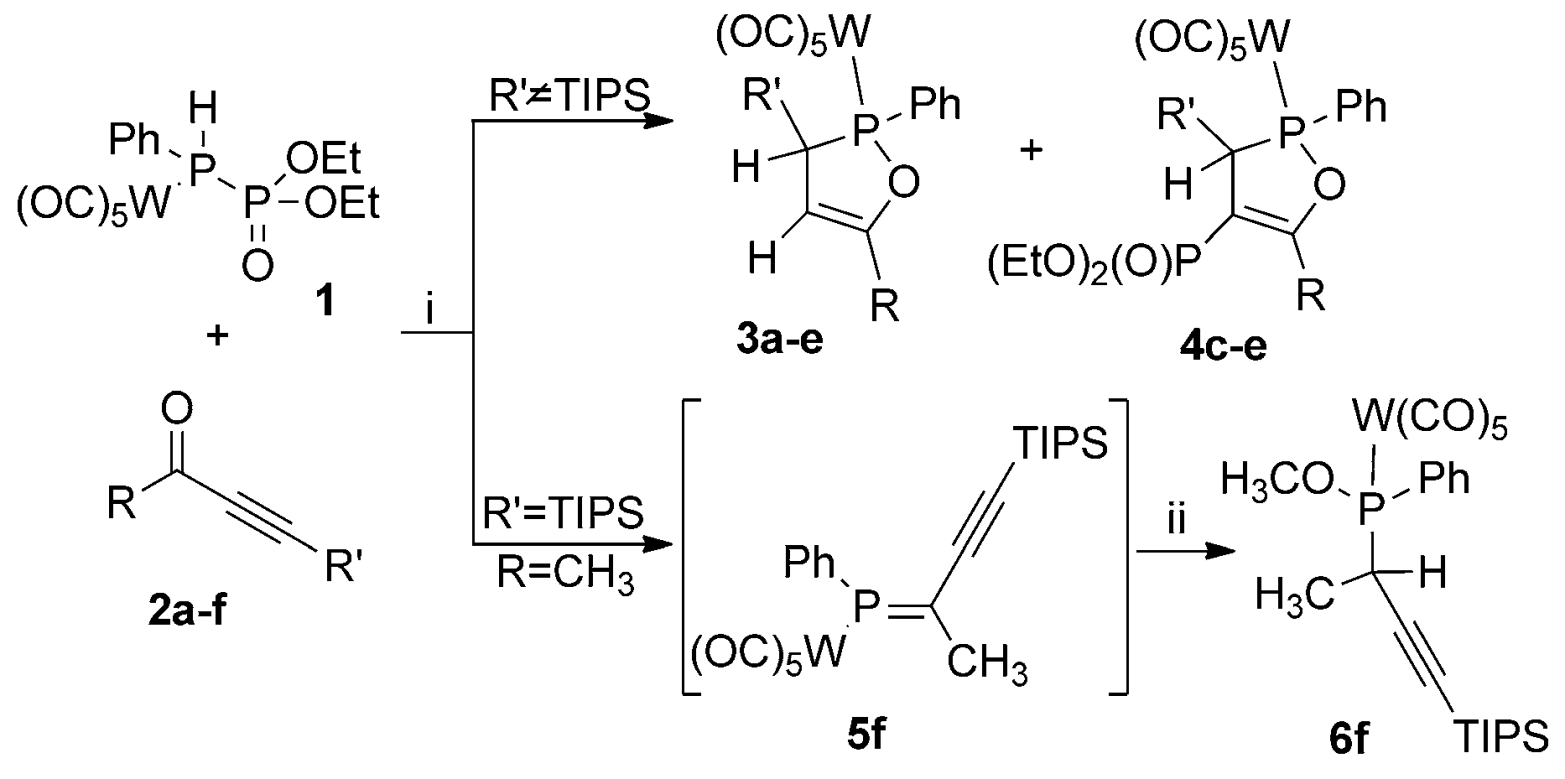
Reaction of 1 with monoacetylenic ketones. i) Lithium diisopropylamide (LDA; 1.1 equiv), −30 °C, 30 min, then 2 (1 equiv), 1 h at −50 °C; ii) MeOH (10 equiv), −50 °C, 30 min. a: R=CH_3_, R′=TMS; b: R=CH_3_, R′=TES; c: R=CH_3_, R′=Ph; d: R=Ph, R′=Ph; e: R=Ph, R′=TES; f: R=CH_3_, R′=TIPS.

**Reaction of phosphinophosphonate 1 with monoacetylenic ketones**: In the majority of cases, the reaction of **1** with monoacetylenic ketones does not produce the, perhaps expected, phosphaalkenes, but instead results in the formation of 1,2-oxaphospholes. As shown in the reaction between ethynyl methyl ketones **2 a**–**c**,**f** or ethynyl phenyl ketones **2 d**,**e** with **1** (Scheme 1 and Table 1), it is clear that the acetylene terminus directs the reaction outcome.

**Table 1:** Product yields for the reaction of 1 with monoacetylenic ketones.

Entry	R	R′	Combined yield [%]^[a]^ products3and4	Yield [%] product6
A	CH_3_	TMS	38	0
B	CH_3_	TES	35	0
C	CH_3_	Ph	33	0
D	Ph	Ph	45	0
E	Ph	TES	43	0
F	CH_3_	TIPS	0	65

[a] Reaction outcome can be directed to either **3** or **4** by appropriate choice of workup procedure as demonstrated for the reaction of **18** with **1** (Scheme 6, Table 3).

**Figure 1:**
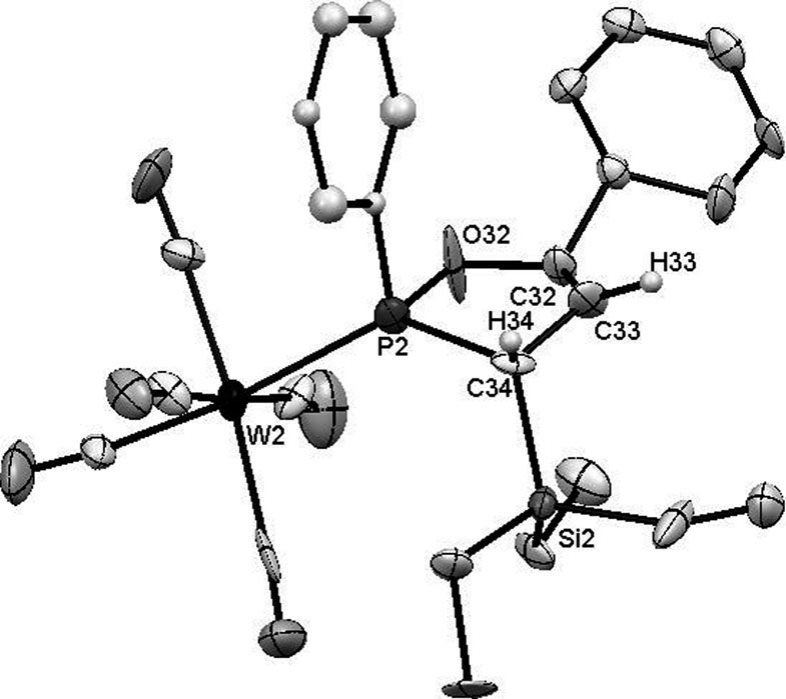
Crystal structure of compound 3 e (ellipsoids set to 50 % probability). All protons are omitted for clarity except for those at the oxaphosphole. Only one of the possible two phenyl ring positions at phosphorus is presented.

With its very bulky (*i*Pr)_3_Si (TIPS) substituent at the acetylene terminus, ketone **2 f** reacts with lithiated **1** (abbreviated as **1Li**) as expected to produce phosphaalkene **5 f**, which is, however, unstable and can only be isolated as its methanol-addition product. Two stereocenters at the former carbonyl carbon atom and the phosphorus center give rise to two diastereomeric products in a ratio of 3:2 (*δ*(^31^P)=134.8 and 134.3 ppm). If less sterically demanding acetylene termini, such as (Et)_3_Si (TES) in ketones **2 b**,**e** or phenyl in ketones **2 c**,**d**, are used, the reaction proceeds very differently and 2,5-dihydro-1,2-oxaphospholes **3 b**–**e** and **4 c**–**e** are obtained as the sole products. Compounds **3** and **4** only differ in the phosphonate group at C4 of the oxaphosphole, which can be removed by basic workup (see below). Oxaphospholes **3** exhibit characteristic ^31^P NMR chemical shifts for compounds with silyl groups at C5 (**3 a**,**b**,**e**; *δ*=132 ppm), whereas those with a phenyl substituent (**3 c**,**d**) resonate at *δ*=149.6 ppm. For compounds **4 b**–**e**, the ^31^P NMR spectra feature AB coupling patterns with typical doublets at chemical shifts of *δ*=161.5 and 16.0 ppm (^3^*J*_PP_=35 Hz) for heterocycle **4 e** and approximately 152 and 15 ppm (^3^*J*_PP_=28 Hz) for compounds **4 c**,**d**. The molecular structure of compound **3 e** was confirmed by X-ray diffraction analysis of single crystals obtained by slow evaporation of a solution in pentane (Figure 1).

In the solid state, the TES group at C5 resides in the sterically least demanding position, *trans* to the *P*-phenyl substituent. In general, it seems that it is the system’s desire to avoid steric clashes between the *P*-bound phenyl group and the bulky C5 substituent that determines the formation of only one diastereomer for **3 b**–**e** and **4 c**–**e**. Supporting this notion, two diastereomeric products can be observed when the steric bulk of C5 is decreased, as in trimethylsilyl (TMS)-substituted **3 a**, which can be isolated as both *trans* and *cis* isomers (*δ*(^31^P)=132.0 and 140.8 ppm, respectively) in a 5:1 ratio.

**Scheme 2:**
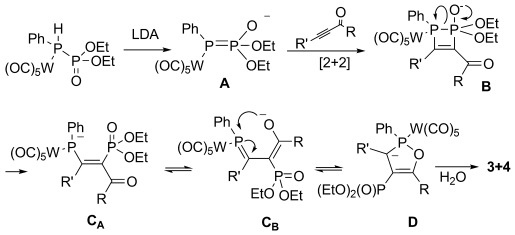
Mechanism for the formation of complexes 3 and 4.

Formation of **3 a**–**e** and **4 c**–**e** can be explained by the mechanism presented in Scheme 2. The sequence is initiated by LDA-promoted abstraction of a proton from **1** to form intermediate **A**. Subsequent [2+2] cycloaddition with the acetylene unit of the ketone results in intermediate **B**; this cycloaddition is prevented if the substituent at the acetylene is too bulky, as in **2 f**. Ring opening of **B** gives intermediate **C**, which can be depicted in several isomeric forms. One of these, **C_B_**, stems from rotation around the newly formed C–C bond and engages in an intramolecular nucleophilic attack that results in the formation of heterocycle **D**. Hydrolysis during aqueous workup gives rise to products **3 a**–**e** and **4 c**–**e**. The reactions with ketones **2 a**–**e** exclusively follow the pathway depicted in Scheme 2. No traces of phosphaalkenes that would arise from the usual phospha-Wittig–Horner reaction could be detected even when the reaction was quenched at low temperatures with MeOH or 1,3-butadiene.

**Scheme 3:**
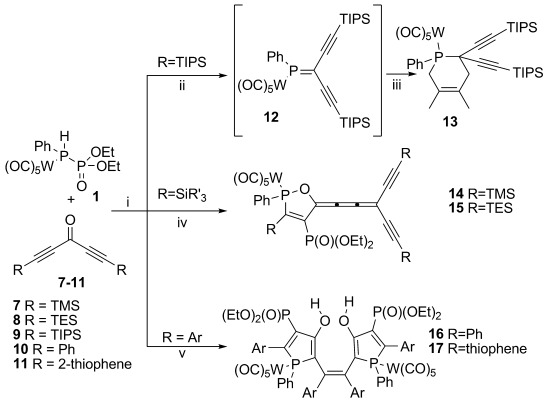
Reaction of 1 with symmetric diacetylenic ketones. i) BuLi (1.1 equiv), −30 °C, 30 min; ii) 9 (1.05 equiv), −78 °C, 30 min; iii) 2,3-dimethylbuta-1,3-diene (10 equiv), −78 °C to RT, 1 h; iv) 7 or 8 (2 equiv), −78 °C to −30 °C, 1.5 h; v) 10 or 11 (1.05 equiv), −50 °C, 1.5–2 h.

**Reaction of 1 with diacetylenic ketones**:

*Symmetric ketones*: Having identified an exclusive pathway for the preparation of 1,2-oxaphospholes, we were prompted to investigate the reactivity of diacetylenic ketones. The reaction of symmetric ketones **7**–**11** with **1** proved to be very complex and highly dependent on the acetylene termini (Scheme 3 and Table 2).

**Table 2:** Yields of products in the reaction of 1 with symmetric diacetylenic ketones.

Ketone	R	Product	Yield [%]	*δ*(^31^P), P^III^ [ppm]	*δ*(^31^P), P^V^ [ppm]	^3^*J*_PP_ [Hz]
**7**	TMS	**14**	7	168.2	6.4	59
**8**	TES	**15**	57	167.5	6.8	63
**9**	TIPS	**13**	35	9.7	–	–
**10**	Ph	**16**	38	38.4	13.6	38
**11**	thiophene	**17**	50	37.2	13.4	32

In analogy to monoacetylenic ketones, substrates with very bulky substituents at the acetylene termini, such as the TIPS groups in **9**, exhibit the traditional phospha-Wittig behavior and afford thermally unstable phosphaalkenes such as **12**, which were detected in situ by ^31^P NMR spectroscopy and trapped with 1,3-butadiene to give compounds like **13**. For the ketones with smaller silyl substituents, such as TES or TMS, the only observed reaction products are cumulenes **14** and **15**, respectively.

**Scheme 4:**
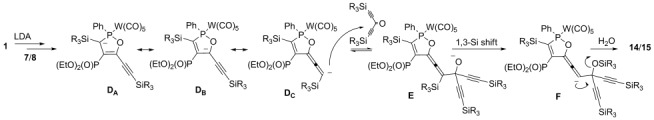
Proposed mechanism of the formation of 14 and 15.

The first steps in the mechanism for the formation of cumulenes **14** and **15** are identical to those depicted in Scheme 2. Due to the presence of a second acetylene unit, intermediate **D** can be described by additional resonance structures **D_B_** and **D_C_** (Scheme 4). Of these, **D_C_** is an allenyl anion that can engage in nucleophilic attack on a second ketone to form intermediate **E**. This adduct can undergo a 1,3-silyl shift to give **F**, in which the newly formed OSiR_3_ can function as a leaving group to generate the observed cumulenes **14** and **15**. Quenching of the reaction mixture with MeOH at low temperature allowed the isolation of protonated intermediate **F**.

**Scheme 5:**
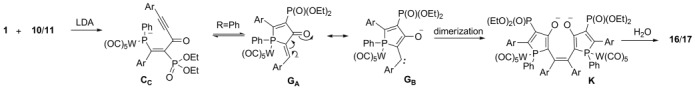
Proposed mechanism for the reaction between aryl-substituted ketones and 1.

Changing the termini at the acetylenes from silyl groups to aromatic groups alters the reactivity of the system dramatically. The only compounds that can be detected from the reaction of **1** with bis(phenylethynyl)ketone **10** and bis(2-thiophenylethynyl)ketone **11** are bisphospholes **16** and **17**, which are isolated as bright-orange and -red solids in 38 and 50 % yield, respectively. Both compounds are characterized by two doublets in their ^31^P NMR spectra at approximately *δ*=37 and 13 ppm, respectively, with coupling constants of around ^3^*J*_PP_=32 Hz. The proposed mechanism for the formation of **16** and **17** is presented in Scheme 5. Again, the first steps, up to intermediate **C**, are identical to those in Schemes 2 and 4. Of the different representations of **C**, **C_C_** contains a formal negative charge at the phosphorus center that enables a 5-*exo* attack at the carbon atom of the second acetylene unit to form intermediate **G_A_**. Although this intermediate can be described as **G_A_** with a localized negative charge, it can also be represented as **G_B_**, which exhibits carbene character. It is this latter resonance form that enables the carbene dimerization to generate **K**. Hydrolysis of **K** during workup affords the final products **16** and **17**.

**Scheme 6:**
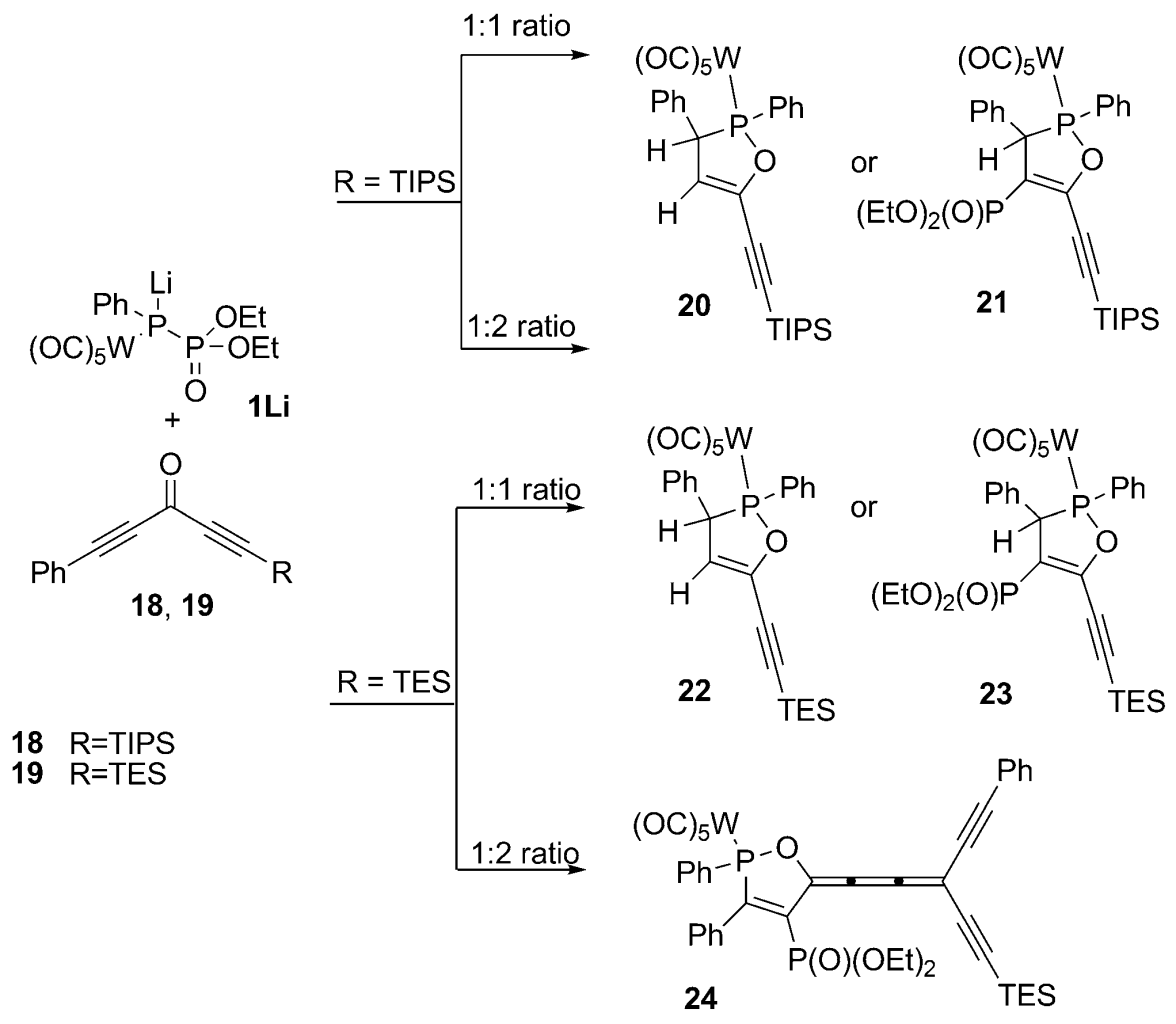
Reaction of 1 with asymmetric diacetylenic ketones 18 and 19.

*Asymmetric ketones*: To differentiate between the reactivity of dissimilarly substituted diacetylenes, as well as to obtain mechanistic insights by determining the rate- and product-determining steps, a series of reactions with asymmetrically disubstituted ketones were performed. Thus, ketones **18**, which carries phenyl and TIPS groups at the acetylene termini, and **19**, having instead phenyl and TES groups, were reacted with **1** (Scheme 6 and Table 3).

**Table 3:** Products yields for the reaction of 1 with asymmetric diacetylenic ketones.

Ketone	Ratio	Product	Yield [%]	*δ*(^31^P), P^III^ [ppm]	*δ*(^31^P), P^V^ [ppm]	^3^*J*_PP_ [Hz]
**18**	1:1	**20**^[a]^	37	150.0	–	–
**18**	1:1	**21**^[b]^	30	155.9	12.4	27
**18**	1:2	**20**^[a]^	31	150	–	–
**19**	1:1	**22**^[a]^	10	150.1	–	–
**19**	1:1	**23**^[b]^	27	156.0	11.7	28
**19**	1:2	**24**^[c]^	–	150.7	6.0	43
				150.5	6.2	43

[a] Reaction was quenched by addition of water at −50 °C. [b] Reaction was quenched by direct application to a silica gel column. [c] Reaction was quenched by direct application on silica after 1.5 h; only the red fraction was collected and concentrated to obtain crude products for ^31^P NMR spectroscopy. Two isomers for complex **24** were found.

**Figure 2:**
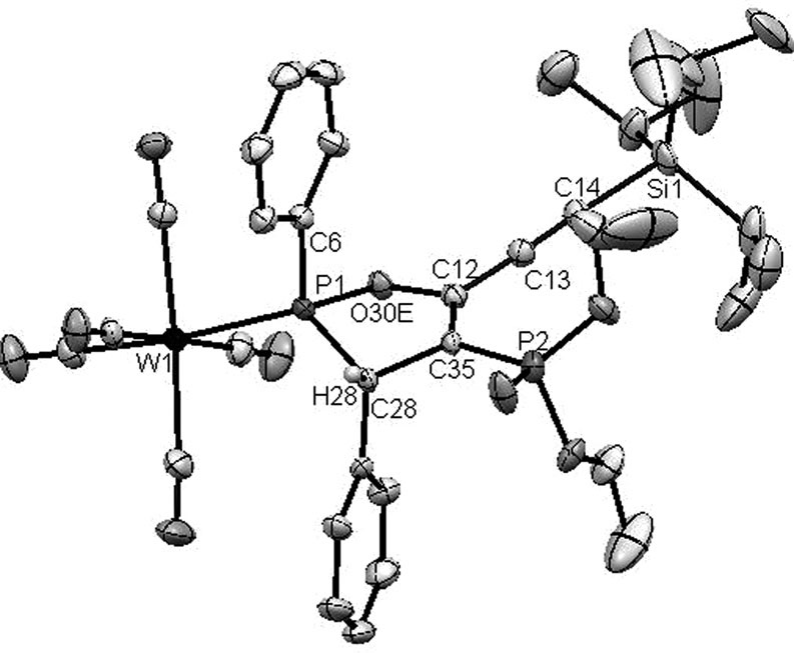
Crystal structure of compound 21 (ellipsoids set to 50 % probability). All protons are omitted for clarity except for those at the oxaphosphole ring.

As expected, the acetylenic TIPS group prevents [2+2] cycloaddition in the early stages of the reaction. It is then not surprising that **1Li** attacks the phenyl-terminated acetylene of **18** to give oxaphospholes **20** (*δ*(^31^P)=150.0 ppm) and **21** (*δ*(^31^P)=155.9 (d, ^3^*J*_PP_=27 Hz, P^III^) and 12.4 ppm (d, *J*=27 Hz; P^V^)), which differ only in the presence or absence of the phosphonate group. This reaction behavior is identical to that described for the monoacetylenic ketones in Scheme 1. It is noteworthy that the ratio of **20** to **21** depends on the pH during workup. Quenching the reaction mixture by filtration through a plug of (acidic) silica gives only **21**, whereas aqueous (basic) workup at −50 °C leads to loss of the phosphonate group and the formation of **20**. The molecular structure of **21** was confirmed by X-ray diffraction analysis of single crystals obtained by evaporation of a solution in CH_2_Cl_2_ at −30 °C (Figure 2).

The TIPS group in **18** not only prevents the [2+2] cycloaddition at its adjacent acetylene in the reaction with **1Li**, but also prevents subsequent chemistry in **20** and **21**. Even if the ratio between **18** and **1** is changed from 1:1 to 1:2, no formation of cumulene-type products is observed, thus supporting the notion that nucleophilic attack of the respective **D_C_** intermediate (Scheme 4) on a second molecule of ketone is not viable with TIPS groups at the acetylene terminus.

Executing the reaction of **1Li** with ketone **19** results in the selective formation of the corresponding oxaphospholes **22** (*δ*(^31^P)=150.0 ppm) and **23** (*δ*(^31^P)=156.0 (d, ^3^*J*_PP_=28 Hz, P^III^) and 11.7 ppm (d, *J*=28 Hz; P^V^)) with their ratio again dependent on the workup (see above). Evidently, also in this case, **1Li** attacks the phenyl acetylene rather than the TES acetylene. It is important to note that **22** and **23** were the exclusive products when the starting materials were used in an equimolar ratio. This indicates that the formation of the oxaphosphole is faster than the nucleophilic attack of intermediate **D_C_** on a second equivalent of the ketone. Thus, if the ratio between ketone **19** and compound **1** was increased to 2:1, the only detectable product was cumulene **24**, which is, however, unstable under the reaction condition and tends to polymerize on workup. Nevertheless, the ^31^P NMR spectrum of the characteristically bright-red crude reaction mixture indicates the presence of two isomeric forms of **24** with chemical shifts of *δ*=150.7 (d, ^3^*J*_PP_=43 Hz, P^III^) and 6.0 ppm (d, *J*=43 Hz; P^V^) for the first isomer and *δ*=150.5 (d, ^3^*J*_PP_=43 Hz, P^III^) and 6.2 ppm (d, *J*=43 Hz; P^V^) for the second. The presence of two isomers can be expected based on the *cis*/*trans* isomers of the butatriene system.

**Scheme 7:**
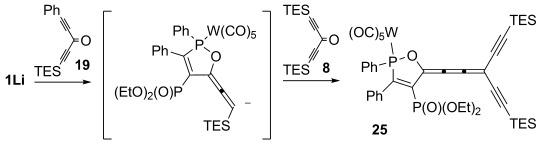
Stepwise reaction of 1 with two different ketones.

*Reaction with two different ketones*: If intermediate **D** with a reactive appended acetylene moiety can indeed be formed selectively, it should also be possible to subsequently react it with a second, different diacetylenic ketone. This hypothesis was tested by successively adding two different ketones to **1Li** in a one-pot procedure (Scheme 7).

Thus, one equivalent of ketone **19** was first added to **1Li** at −78 °C. After stirring the resulting dark-red solution for 30 min, one equivalent of ketone **8** was added and the reaction mixture was allowed to warm to −10 °C. Direct quenching of the reaction mixture on silica gel allowed the isolation of **25** as the sole product. Other cumulenes that would arise from scrambling of the ketones were not observed, nor were any oxaphospholes of type **22** or **23**. The exclusive formation of **25** convincingly illustrates the control that is possible during the reaction sequence, allowing the selective introduction of different substituents at C5 of the oxaphosphole and at the acetylene termini.

NMR studies

*^31^P NMR studies*: The ^31^P chemical shifts of all compounds are summarized in Tables 2, 3, and 4. Those of oxaphospholes **3**, **20**, and **22** are at around *δ*=150 ppm, whereas that for the C5-phenyl-substituted **3 e** resonates at higher field (*δ*=132 ppm). The presence of a P^V^ substituent at C4 in **4**, **21**, and **23** (resonating at *δ*=12–16 ppm) causes a downfield shift of the P^III^ resonance. The cumulene substituent in **14**, **15**, and **25** induces an upfield shift of the P^V^ resonance of approximately *δ*=6 ppm, enabling the unambiguous identification of these compounds from the crude reaction mixtures.

**Table 4:** ^31^P NMR chemical shifts and coupling constants for oxaphospholes.

Compound	*δ*(^31^P) [ppm]	Compound	*δ*(^31^P^III^) [ppm]	*δ*(^31^P^V^) [ppm]	^1^*J*_PP_ [Hz]
**3 a**	132.0	–	–	–	–
**3 b**	132.0	–	–	–	–
**3 c**	149.6	**4 c**	153.0	15.8	28
**3 d**	149.6	**4 d**	151.2	14.8	28
**3 e**	132.8	**4 e**	161.5	16.0	36
**20**	150.0	**21**	156.0	12.4	27
**22**	150.1	**23**	156.0	11.7	27

**Figure 3:**
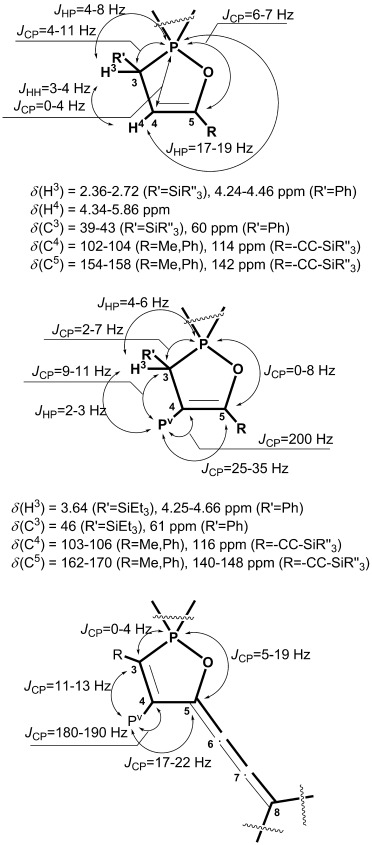
Summary of the chemical shifts and carbon–phosphorus and proton–carbon coupling constants for ring carbon atoms and protons.

*^1^H and* ^*13*^*C NMR studies*: Because oxaphospholes are still rather rare heterocycles, we report their ^1^H and ^13^C NMR spectroscopic signatures in detail (Figure 3 and Table 4). The H^3^ proton in **3** and **4** is featured as a multiplet due to ^2^*J*_HP_ (ca. 4–8 Hz) and ^3^*J*_HH_ (3–4 Hz) or ^3^*J*_HP_ (ca. 3 Hz) couplings, respectively. The chemical shift of the C^3^ carbon atom in compounds bearing a phenyl group is generally deshielded compared to that of the silyl-substituted derivatives. ^1^*J*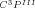 coupling constants of the P^III^ center are in the range of 7–11 Hz for the compounds with R′=Ph and 2–6 Hz for those with R′=TES. For silyl-substituted cumulenes **14** and **15** this coupling constant was not detected, whereas it is 4 Hz for the phenyl-substituted analogue. Coupling between the C^3^ and P^V^ atoms is generally 9–13 Hz and is independent of the ring substitution pattern. Another characteristic resonance in the ^13^C NMR spectra of **4**, **21**, and **23** is observed for the C^4^ carbon atom, which has a rather large ^1^*J*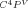 coupling constant of up to 200 Hz. The presence of acetylene substituents at C^5^ in **20**–**23** gives rise to an upfield shift of approximately *δ*=15 ppm compared to those of the other oxaphospholes.

**Scheme 8:**
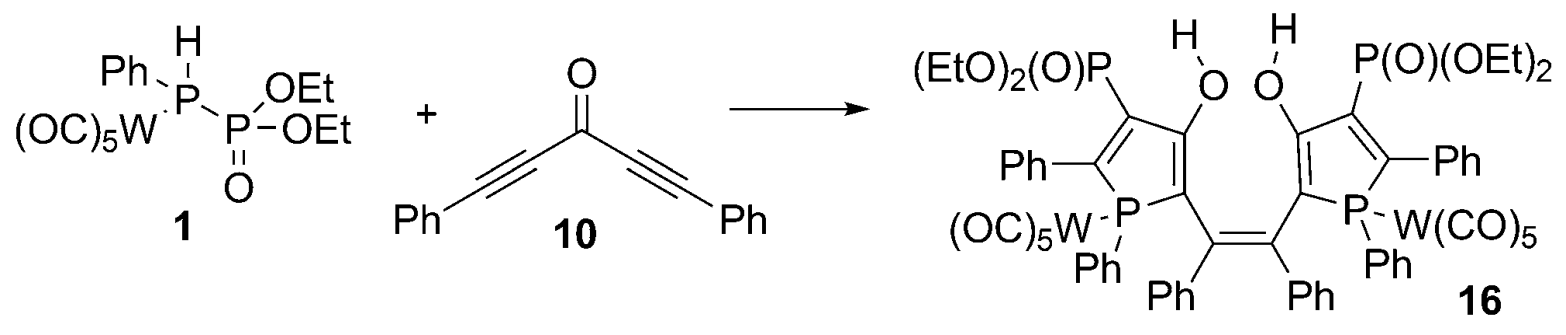
Reaction followed by in situ FTIR spectroscopy.

**Figure 4:**
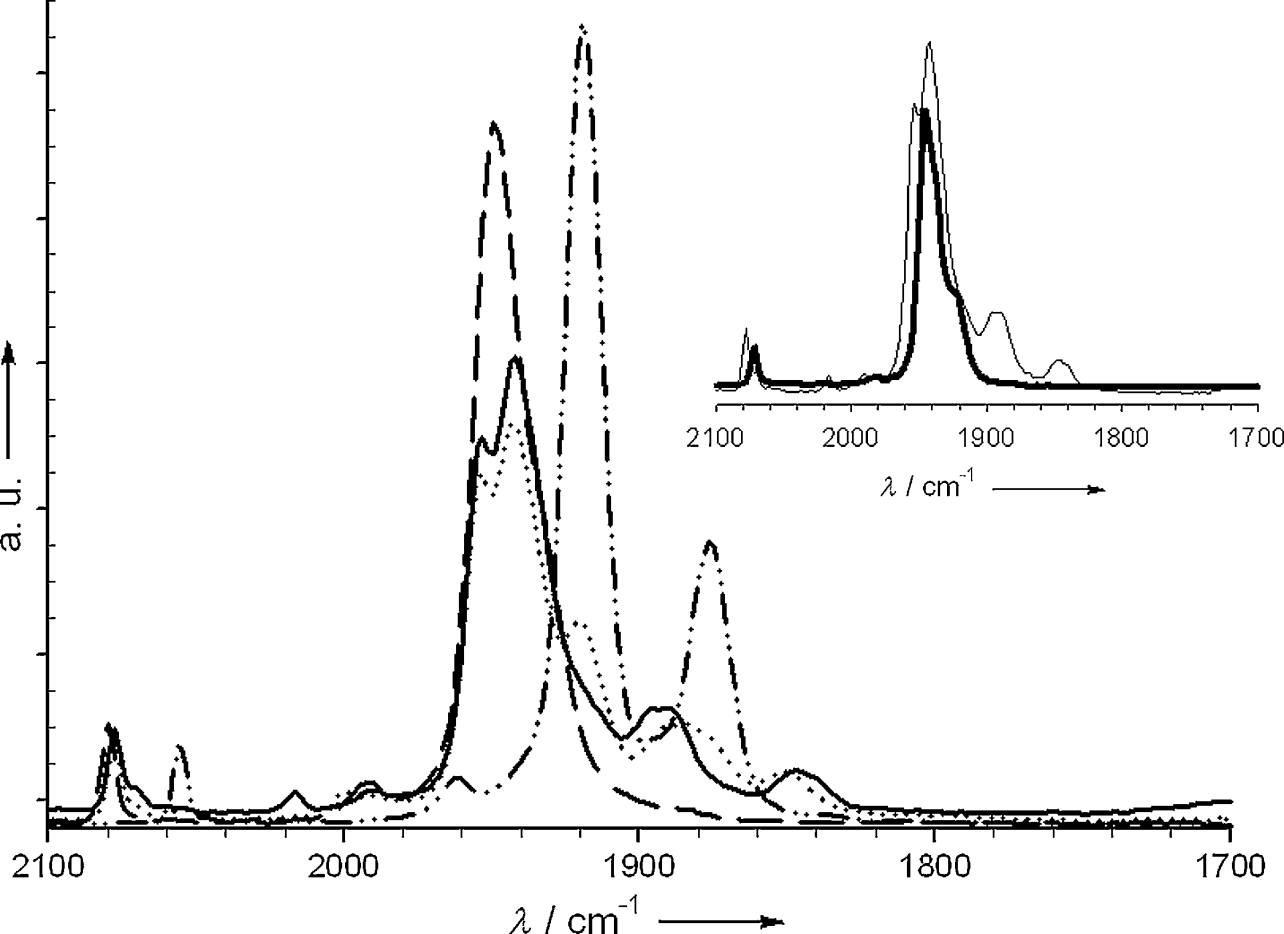
In situ IR spectra obtained during the reaction of 1 (- - - -; 43 mm in THF at −50 °C) with BuLi (–••–••), followed by addition of 10 (43 mm) before (••••) and after (—) quenching with water at the same temperature. Inset: overlay of the final crude IR spectrum and that of purified 16 (thick line).

**Reaction monitoring by in situ IR spectroscopy**: The CO stretching vibrations of the *P*-bound {W(CO)_5_} fragment provide convenient spectroscopic handles to detect chemical transformations at the W-coordinated phosphorous center. We examined the behavior of the carbonyl vibrational frequencies by following the reaction of **1** and **10** to form bisphosphole **16** by in situ FTIR spectroscopy (Scheme 8 and Figure 4).

Compound **1** exhibits two absorptions in the typical carbonyl region at $\tilde \nu $
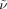
_CO_=2080 (w) and 1950 cm^−1^ (s; Figure 4). Upon treatment with BuLi, new bands appear at $\tilde \nu $
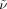
_CO_=2060, 1920, and 1875 cm^−1^, while those of **1** disappear within minutes, indicating the fast and complete lithiation of **1**. On addition of ketone **8**, **1Li** is consumed within 5 min with concomitant emergence of several very broad bands, most prominently at $\tilde \nu $
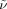
_CO_=2080, 1940, 1880, and 1840 cm^−1^. The multiple vibrational frequencies indicate high complexity of the reaction mixture and the formation of various side products, which could not, however, be isolated. The IR spectrum of the reaction mixture remains in essence unchanged, even after quenching with water. Absorptions that result from bisphosphole **16** at $\tilde \nu $
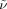
_CO_=2077 and 1936 cm^−1^ can be observed in the reaction mixture and are identical to those of the purified product (inset Figure 4).

**Calculations**: The reaction mechanism proposed to explain the observed products was scrutinized by using DFT calculations. In particular, insights were sought into the reasons why aryl groups at the acetylene termini give rise to bisphospholes, whereas silyl groups promote cumulene-decorated oxaphosphole formation. To reduce computational time, the following modifications were made: ethyl groups in the phosphonate were substituted by methyl groups, the transition metal was changed to chromium, and functional groups at the acetylenic termini that do not participate in the immediate reaction were changed to hydrogen atoms.

**Scheme 9:**
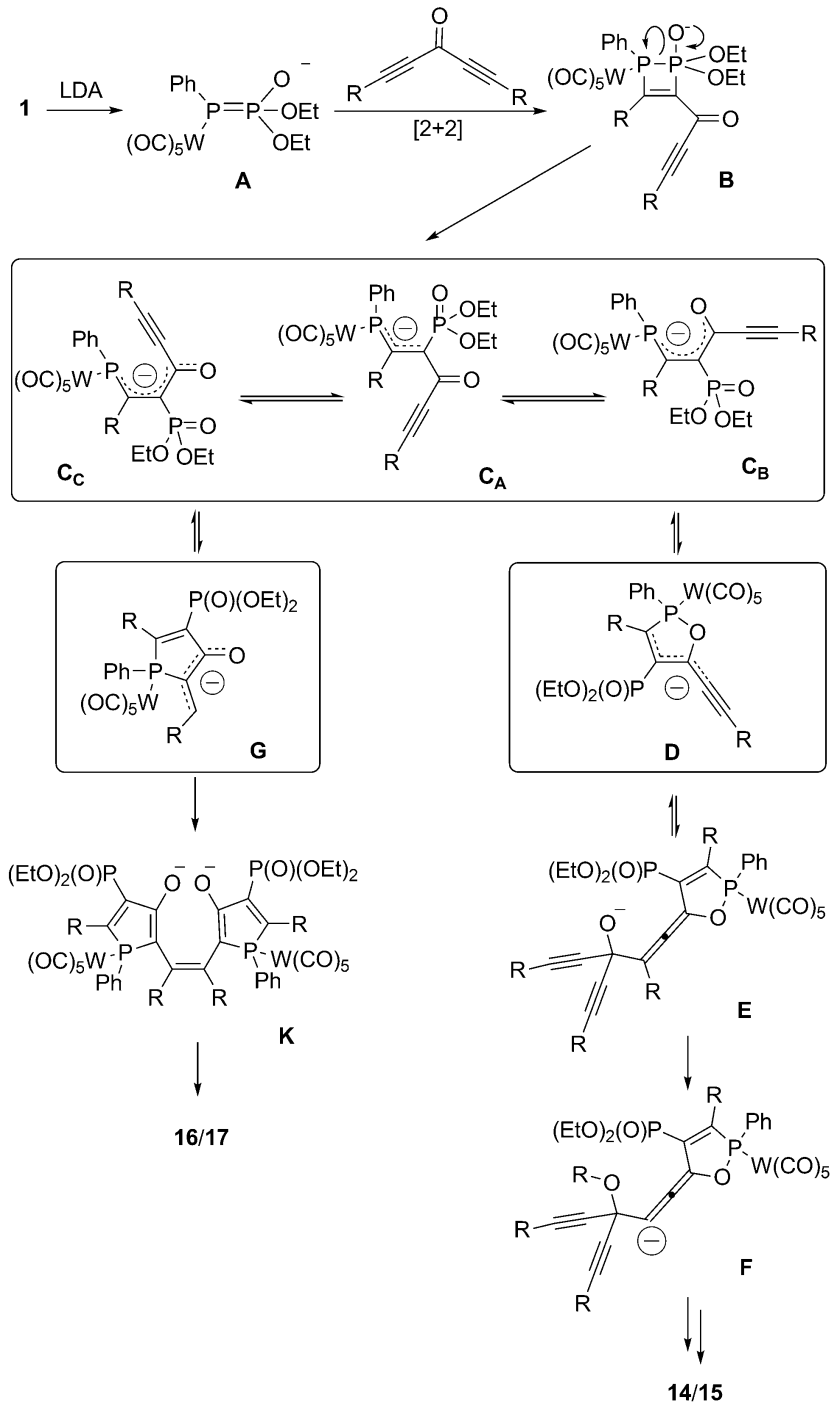
General mechanism for the reaction of 1 with diacetylenic ketones.

Scheme 9 summarizes all (model) intermediates with R being either Ph or TMS; in the text this is identified as subscripts, that is, **D_R=Ph_** and **D_R=TMS_**, respectively. For simplicity, the P–P bond cleavage (**B**→**C**) and the carbene dimerization (**G**→**K**), can be considered irreversible, while all other intermediates are presumably in equilibrium. This assumption focuses the calculations on the distinguishing factors of **C** and the conversion of **C** to **D** and on to **F** and the dimerization to form **K** via **G**.

First, we investigated whether there is a conformational preference for intermediate **C** that depends on the substituent R (Table 5), and whether such a preference could explain the product selectivity. Namely, conformer **C_C_** forecasts phosphole formation (**G**), whereas **C_B_** is more similar to **D**. DFT calculations on the three conformers **C_A_**, **C_B_**, and **C_C_** reveal that **C_A_** is less favorable by at least 1.4 and 2.2 kcal mol^−1^ for phenyl and TMS substitution, respectively. However, no clear conformational preference could be found for either **C_B_** or **C_C_** that would depend on the substituents. This prompted us to investigate subsequent steps of the suggested mechanism.

**Table 5:** Relative B3LYP/6-31G^*^ energies of the intermediates C for R=Ph and R=TMS.

Entry	C_A_	C_B_	C_C_	C_A_	C_B_	C_C_
R	TMS	TMS	TMS	Ph	Ph	Ph
Δ*E* [kcal mol^−1^]	0	−2.4	−2.2	0	−1.4	−1.5

Phosphole formation (**C_C_**→**G**) is slightly endothermic for both the phenyl- and silyl-substituted derivatives (by 4.9 and 8.7 kcal mol^−1^, respectively), whereas oxaphosphole formation (**C_B_**→**D**) is energetically favored by 6.2 and 17.1 kcal mol^−1^, respectively (Table 6). Assuming that intermediates **C**, **D**, and **G** are in equilibrium, the **G**→**D** transformation is thus calculated to be downhill by 24.2 kcal mol^−1^ for the TMS-substituted derivative, but by only 9.6 kcal mol^−1^ for the Ph-substituted derivative. Put differently, the formation of **D_R=TMS_** is greatly favored, whereas **G_R=Ph_**, although endothermic, may still be accessible under the experimental conditions.

**Table 6:** Relative B3LYP/6-31G^*^ energies (in kcal mol^−1^) for the cyclizations and dimerizations.

R	C_C_→G	C_B_→D	G′→K′	D′→E′	TS(D′→E′)
TMS	8.7	−17.1	−17	−27	−12
Ph	4.5	−6.2	−23	−17	+10

**Figure 5:**
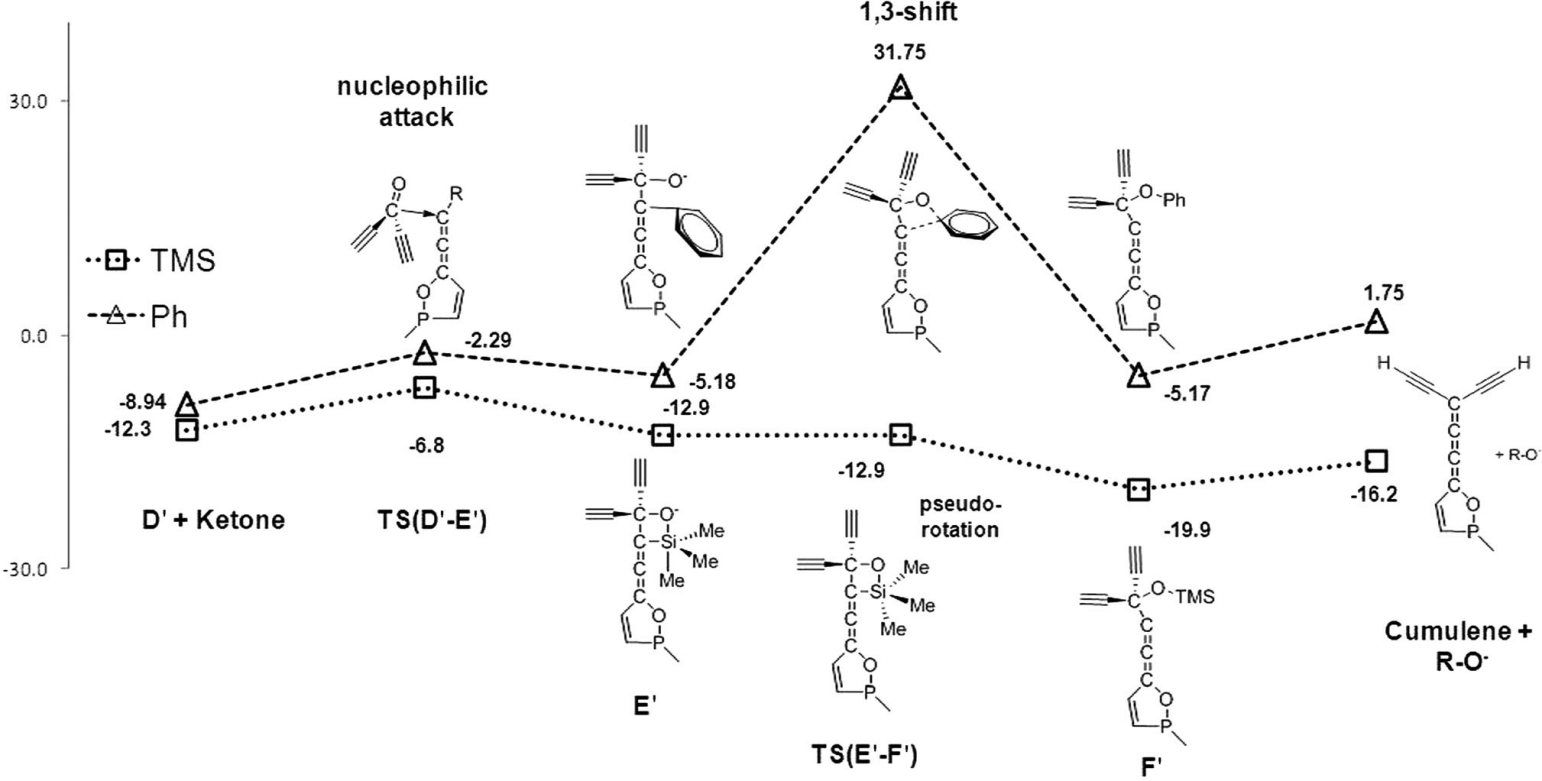
Reaction coordinate for the formation diacetylenic cumulenes 14/15.

Next, the energetics of the nucleophilic attack of **D** on a second ketone (**D**→**E**) and the subsequent 1,3-R shift (R=TMS or Ph) was investigated by using the simplified structures **D′**, **E′**, and **F′** in Figure 5. The transition-state energies for the nucleophilic attack towards the diacetylenic ketone (**TS(D′**–**E′)**) were found to be low lying irrespective of the substituents, suggesting reversibility of this elemental step. Interestingly, there is a distinct difference in the stationary points **E′_R=Ph_** and **E′_R=TMS_**. Whereas the former shows the expected twisted arrangement of the phenyl substituent, the latter involves the formation of a Si–O bond and thus a pentacoordinated Si center with one methyl group and the O atom in the axial positions. In this case, the almost isoenergetic transition state of the TMS migration resembles a “Berry-pseudorotation”,^24^, ^25^ which brings the O atom into an equatorial position and the terminal allene C atom into the elongated axial position that promotes the bond rupture. In contrast, the transition state for the phenyl migration **TS(E′**–**F′)_R=Ph_** is a typical 1,3-shift of significantly higher energy with a relative barrier of approximately 37 kcal mol^−1^. Thermodynamic considerations of the final elimination result in the TMS-substituted reaction being exothermic by 16 kcal mol^−1^, whereas the Ph-substituted reaction is slightly endothermic (+2 kcal mol^−1^).

**Scheme 10:**
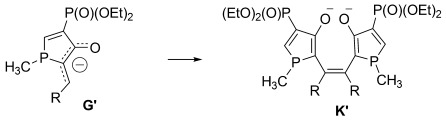
Structures of simplified model compounds for the dimerization of G′ to form bisphospholes.

Bisphosphole formation (**G**→**K**) was theoretically investigated by using the simplified structures presented in Scheme 10, and was found to be energetically favorable for the TMS- and phenyl-substituted compounds by 17 and 23 kcal mol^−1^, respectively. The dimerization step, **G′→K′**, is thus more exothermic for the phenyl- than for the TMS-substituted case, which somewhat explains the preferred bisphosphole formation for phenyl-substituted compounds.

In conclusion, the differences in the reactivity for the compounds with two different substituents mainly originate from the final silyl or phenyl migration converting intermediate **E** into **F**. The phenyl migration proceeds through a high-energy transition state that is inaccessible at low temperatures, whereas the silyl shift proceeds without a significant barrier. On the other hand, the bisphosphole formation is much more exothermic for the phenyl substituent (≈20 kcal mol^−1^) than for the TMS-containing compound (≈10 kcal mol^−1^). Moreover, the **C_C_**→**G** transformation is thermodynamically more costly for R=TMS than for R=Ph (Table 6).

**UV/Vis spectroscopic and electrochemical characterization**: The electronic absorption properties of selected compounds are summarized in Table 7. Cumulenes **15** and **25** are bright-red compounds with longest-wavelength absorption maxima at 470 and 460 nm, respectively. It is interesting to note that introduction of a phenyl substituent instead of a TES group on the C3 carbon atom of oxaphosphole **25** leads to a hypsochromic shift of 10 nm (compared to **15**) and a large reduction in the extinction coefficient. Comparison of the optical properties with those of a peracetylenic butadiyne (*λ*_max_=424 nm)^26^ reveals that the longest wavelength absorption maxima of **15** and **25** are bathochromically shifted by about 40 nm. It is thus clear that the lowest energy absorptions are not isolated within the cumulene-based π system, but rather extend over the entire oxaphosphole unit.

**Table 7:** Electronic absorption spectra of compounds 15–17 and 25.^[a]^

Compound	*λ*_max_ [nm] (*ε* [m^−1^ m^−1^×10^3^])
cumulenes	**15**	303 (47.3), 322 (47), 342 (47.2), 470 (86)
	**25**	300 (19), 334 (13.6), 460 (14.4)
bisphospholes	**16**	316 (16.6), 445 (7.3)
	**17**	329 (21.7), 484 (11.8)

[a] Measured in CH_2_Cl_2_.

The lowest-energy absorption maxima of bisphospholes **16** and **17** differ by approximately 40 nm, indicating a sizeable contribution of the peripheral thiophene substituents to the compounds’ frontier molecular orbitals. The absorptions at 445 and 484 nm for **16** and **17**, respectively, are considerably redshifted compared, for example, to 2,5-bis(thienyl)phosphole (*λ*_max_=412 nm)9c, indicating that the unsaturated ethylene bridge between the two phosphole units mediates a certain degree of delocalization.

The electrochemical behavior of the compounds was studied by cyclic voltammetry and the results are summarized in Table 8. Simple oxaphospholes **20** and **23** with acetylene substituents at C5 are inert to reductive processes within the solvent window and feature only one irreversible oxidation at a potential of 1.27 and 1.12 V versus Fc^+/0^, respectively. The difference of 150 mV arises from the presence of the phosphonate group in **23**, which seems to contribute to the compounds’ HOMOs and shifts the potential cathodically. Expanding the π system further by inclusion of the cumulene portion in **15** and **25** shifts the oxidation potential further cathodically by another 160 and 180 mV, respectively, compared to that of **23**. Reversible reductive processes that are observed for **15** and **25** at around −1.4 V are presumably localized on the π-conjugated portions of the compounds, as they are almost identical to the values reported for peracetylenic butatriynes.^27^ The effect that the oxaphospholes in **15** and **25** have on the electronic properties of the compounds is thus comparable to that imposed by two additional acetylene substituents.

**Table 8:** Electrochemical properties of compounds 15, 16, 17, 20, 23, and 25.^[a]^

	Compound	Reduction *E*_cp_ [V]	Oxidation *E*_pa_ [V]
cumulenes	**15**^[c]^	−1.81	−1.40^[b]^	0.94	1.36
	**25**	−1.67	−1.35^[b]^	0.96	–
bisphospholes	**16**	−2.38	−2.00	0.43	1.03
	**17**	−2.34	−1.87	0.35	1.14
oxaphospholes	**20**	–	–	1.27	–
	**23**	–	–	1.12	–

[a] Measured for 1 mm solutions of the analyte in CH_2_Cl_2_ (0.1 m NBu_4_PF_6_), glassy C electrode, *ν*=100 mV s^−1^. All potentials are given versus F^c+/0^. [b] Peak is reversible, reported value corresponds to *E*_1/2_=(*E*_pa_+*E*_pc_)/2. [c] Measured for a 0.6 mm solution due to low solubility of the complex.

Bisphospholes **16** and **17** are oxidized at relatively mild potentials of 0.43 and 0.35 V, respectively, with the presence of the electron-rich thiophene unit in **17** being responsible for a cathodic shift of 80 mV. In comparison, {W(CO)_5_}-coordinated 2,5-bis(thienyl)phosphole has an oxidation potential of 0.70 V.9c In the cathodic scan, electrochemically reversible one-electron reductions are observed at −2.00 and −1.87 V for **16** and **17**, respectively. It is thus interesting to note that the thiophene substituents in **17** shift both the reduction and oxidation potentials to smaller absolute values. This result is consistent with the UV/Vis spectrum that shows **17** to have the lowest energy absorption maximum of this series.

## Conclusion

We have shown that phosphinophosphonate **1** reacts preferentially in the Michael-addition position of acetylenic ketones. The observed behavior is fundamentally different to that of classical Horner–Wadsworth–Emmons reactions, which react in a 1,2-fashion and convert acetylenic ketones to the corresponding alkenes.^28^ The reactivity of **1** can, however, be changed to the usual attack at the carbonyl carbon atom by the presence of sterically demanding (*i*Pr)_3_Si termini on the acetylenes. In all other cases, **1** reacts with monoacetylenic ketones **2 a**–**e** to afford highly functionalized 2,5-dihydro-1,2-oxaphospholes **3** and **4**. The presented approach provides reliable access to this rather rare class of heterocycles.

Inclusion of a second acetylene in the ketone substrate promotes further reactivity of the system. Diacetylenic ketones that contain at least one silyl substituent as an acetylene terminus also form 2,5-dihydro-1,2-oxaphospholes, which react further with a second equivalent of ketone to form an appended cumulene framework. The reaction sequence allows a high degree of control as the two diacetylenic ketones can be added successively and substituents at the oxaphosphole and the cumulene can thus be introduced selectively. Completely different reactivity is observed when both acetylene moieties of the diethynylketones are terminated by aromatic groups. In this case, ethenyl-bridged bisphospholes are formed.

The initial steps for the preparation of both types of compounds are identical, but diverge after the ring-opening of a putative oxadiphosphetane intermediate. DFT computational studies suggest that many of the initial reaction intermediates are in equilibrium and that bisphosphole formation is significantly more exothermic for ketone substrates with aromatic acetylene termini. Moreover, the formation of cumulenic oxaphospholes is highly favored for silyl-terminated ethynylketones. In contrast to the easily accessible 1,3-silyl shift to form cumulenes, the corresponding putative phenyl shift proceeds through a large relative barrier that completely inhibits the aryl migration while the silyl rearrangement is greatly favored.

## Experimental Section

**General**: All reactions were performed under argon by using modified Schlenk techniques. Diethyl ether and THF were freshly distilled from sodium/benzophenone prior to use. ^1^H, ^13^C, and ^31^P spectra were recorded on a JEOL Eclipse+ and a Varian Mercury+ operating at proton frequencies of 400 and 300 MHz, respectively. Chemical shifts are reported in ppm and referenced internally to residual solvent peaks (^1^H, ^13^C) or externally to aqueous H_3_PO_4_ (85 %). High-resolution mass spectral analyses (HRMS) were performed on a high resolution and FTMS+pNSI mass spectrometer (OrbitrapXL). Ketones **2 a**–**f** were prepared following literature procedures (for references please see the Supporting Information).

**X-ray data**: CCDC-848398 http://www.ccdc.cam.ac.uk/cgi-bin/catreq.cgi(**3 e**) and CCDC-933297 http://www.ccdc.cam.ac.uk/cgi-bin/catreq.cgi(**21**) contain the supplementary crystallographic data for this paper. These data can be obtained free of charge from The Cambridge Crystallographic Data Centre via http://www.ccdc.cam.ac.uk/data_request/cif. A summary of the data can be found in Table SI1 of the Supporting Information.

**General procedure for the reaction of 1 with monoacetylenic ketones**: Compound **1** (1 equiv) was dissolved in THF (5–15 mL) and the solution was cooled to −20 °C. Subsequently, LDA (1 equiv, 2 m in THF/heptanes) was added dropwise and the reaction mixture was stirred for 20 min. After this time, the solution was cooled to −78 °C and the ketone (1.05 equiv in THF) was added dropwise. The reaction mixture was stirred for 1.5 h at −50 °C and directly poured onto a silica gel plug and washed with THF. After removal of all volatile compounds, the residue was subjected to column chromatography to give the final product.

**Oxaphosphole 3 a**: Prepared from **1** (0.15 g, 0.26 mmol) and ketone **2 a** (0.04 g, 0.27 mmol). The final compound was obtained as a colorless oil (0.04 g, 38 %, mixture of two isomers in a 4:1 ratio) after chromatography by using CH_2_Cl_2_ (10 %) in pentane as eluent (*R*_f_=0.57). Major isomer: ^31^P NMR (161 MHz, CDCl_3_): *δ*=132 ppm (^1^*J*_PW_=277 Hz); ^1^H NMR (400 MHz, CDCl_3_): *δ*=7.42–7.39 (m, 2 H; Ph), 7.35–7.30 (m, 3 H; Ph), 4.81 (ddq, ^3^*J*_PH_=18, ^3^*J*_HH_=4, ^5^*J*_HH_=1 Hz, 1 H; *H*C=), 2.36–2.34 (m, 1 H; *H*CP), 2.03 (dd, ^4^*J*_HP_=3, ^4^*J*_HH_=1 Hz, 3 H; CH_3_), 0.26 ppm (s, 9 H; TMS); ^13^C NMR (100 MHz, CDCl_3_): *δ*=198.6 (d, *J*=26 Hz), 196.1 (d, *J*=7 Hz), 154.8 (d, *J*=7 Hz), 146.2 (d, *J*=26 Hz), 129.5 (d, *J*=2 Hz), 128.2 (d, *J*=9 Hz), 125.5 (d, *J*=12 Hz), 101.9 (d, *J*=3 Hz), 42.5 (d, *J*=4 Hz), 15.6 (d, *J*=4 Hz), −1.7 ppm (d, *J*=2 Hz); Minor isomer: ^31^P NMR (161 MHz, CDCl_3_): *δ*=140.8 ppm (^1^*J*_PW_=274 Hz); ^1^H NMR (400 MHz, CDCl_3_): *δ*=7.51–7.48 (m, 2 H; Ph), 7.45–7.43 (m, 3 H; Ph), 4.78 (ddq, ^3^*J*_PH_=19, ^3^*J*_HH_=2, ^4^*J*_HH_=1 Hz, 1 H; *H*C=), 3.08–3.06 (m, 1 H; *H*CP), 2.10 (dd, ^4^*J*_HP_=3, ^3^*J*_HH_=1 Hz, 3 H; CH_3_), −0.22 ppm (s, 9 H; TMS); ^13^C NMR (100 MHz, CDCl_3_): *δ*=199.2 (d, *J*=27 Hz), 196.3 (d, *J*=8 Hz), 155.9 (d, *J*=6 Hz), 137.9 (d, *J*=28 Hz), 131.3 (d, *J*=2 Hz), 129.9 (d, *J*=15 Hz), 128.4 (d, *J*=10 Hz), 99.9 (d, *J*=3 Hz), 44.7 (d, *J*=7 Hz), 15.3 (d, *J*=5 Hz), −1.8 ppm (d, *J*=2 Hz); IR (pentane): $\tilde \nu $
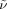
=2076, 1989, 1956, 1943, 1942, 1914, 1665 cm^−1^; HRMS (solution in MeOH): *m*/*z* calcd for C_18_H_21_O_7_PSiWNa: 615.02012 [*M*+H_2_O+Na]^+^; found: 615.01994.

**Oxaphosphole 3 b**: Prepared from **1** (0.156 g, 0.27 mmol) and ketone **2 b** (0.05 g, 0.27 mmol). The final compound was obtained as a colorless oil (0.056 g, 35 %) after column chromatography by using pentane/CH_2_Cl_2_ (95:5) as eluent (*R*_f_=0.26). ^31^P NMR (161 MHz, C_6_D_6_): *δ*=132.0 ppm (^1^*J*_PW_=277 Hz); ^1^H NMR (400 MHz, C_6_D_6_): *δ*=7.34–7.29 (m, 2 H; Ph), 7.10–7.05 (m, 2 H; Ph), 6.96–6.92 (m, 1 H; Ph), 4.34 (ddq, ^3^*J*_HP_=19, ^3^*J*_HH_=4, ^4^*J*_HH_=2 Hz, 1 H; *H*C=), 2.35 (ddq, ^2^*J*_HP_=7, ^3^*J*_HH_=4, ^5^*J*_HH_=2 Hz, 1 H; *H*CP), 1.60 (dd, ^4^*J*_HP_=4, ^4^*J*_HH_=2 Hz, 3 H; CH_3_), 0.96 (t, ^3^*J*_HH_=8 Hz, 9 H; CH_3_CH_2_Si), 0.85–0.76 ppm (m, 6 H; CH_3_C*H*_2_Si); ^13^C NMR (100 MHz, C_6_D_6_): *δ*=198.4 (d, *J*=26 Hz), 196.3 (d, ^1^*J*_PW_=126, ^1^*J*_PC_=7 Hz), 154.4 (d, *J*=6 Hz), 146.5 (d, *J*=26 Hz), 129.5 (d, *J*=2 Hz), 128.3 (d, *J*=9 Hz), 125.5 (d, *J*=12 Hz), 102.0 (d, *J*=3 Hz), 39.2 (d, *J*=5 Hz), 15.1 (d, *J*=4 Hz), 7.5 (s), 3.3 ppm (d, *J*=2 Hz); HRMS (solution in CHCl_3_ with addition of AgCF_3_COO): *m*/*z* calcd for C_42_H_50_O_12_Si_2_P_2_W_2_Ag: 1341.03996 [2*M*+Ag]^+^; found: 1341.04221.

**Oxaphospholes 3 c and 4 c**: Prepared from **1** (0.31 g, 0.55 mmol) and ketone **2 c** (0.08 g, 0.55 mmol). Final compounds were obtained after chromatography by using diethyl ether (1 %) in pentane as eluent to give pure **3 c** (*R*_f_=0.11; 0.016 g, 5 %) followed by pure diethyl ether to give **4 e** (*R*_f_=0.46, 0.11 g, 28 %) as white solids.

*Compound **3 c***: ^31^P NMR (121 MHz, CDCl_3_): *δ*=149.6 ppm (^1^*J*_PW_=287 Hz); ^1^H NMR (300 MHz, CDCl_3_): *δ*=7.56–7.52 (m, 3 H; Ph), 7.47–7.33 (m, 3 H; Ph), 7.30–7.27 (m, 3 H; Ph), 7.20–7.17 (m, 1 H; Ph), 5.09 (dd, ^2^*J*_HP_=18, ^3^*J*_HH_=3 Hz, 1 H; HC=), 4.26–4.21 (m, 1 H; HCPh), 2.19 ppm (br s, 3 H; =CCH_3_); ^13^C NMR (75 MHz, CDCl_3_): *δ*=198.7 (d, *J*=29 Hz), 194.8 (d, *J*=8 Hz), 158.3 (d, *J*=7 Hz), 142.9 (d, *J*=30 Hz), 138.4 (d, *J*=5 Hz), 130.2 (d, *J*=2 Hz), 129.2 (d, *J*=3 Hz), 129.0 (d, *J*=5 Hz), 128.6 (d, *J*=9 Hz), 128.0 (d, *J*=4 Hz), 126.4 (d, *J*=12 Hz), 103.7 (s), 59.8 (d, *J*=11 Hz), 15.9 ppm (d, *J*=3 Hz); IR (CH_2_Cl_2_): $\tilde \nu $
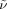
=2306, 2076, 1946, 1605, 1198, 1118 cm^−1^; HRMS (solution in MeOH/CHCl_3_ with addition of AgCF_3_COO): *m*/*z* calcd for C_21_H_15_O_6_PWAg: 684.91665 [*M*+Ag]^+^; found: 684.91528.

*Compound **4 c***: ^31^P NMR (121 MHz, CDCl_3_): *δ*=153.0 (d, ^3^*J*_PP_=28, ^1^*J*_PW_=293 Hz; P^III^), 15.8 ppm (d, ^3^*J*_PP_=28 Hz, P^V^); ^1^H NMR (300 MHz, CDCl_3_): *δ*=7.53–7.49 (m, 4 H; Ph), 7.44–7.41 (m, 1 H; Ph), 7.39–7.34 (m, 3 H; Ph), 7.28–7.23 (m, 2 H; Ph), 4.26–4.23 (m, 1 H; *H*CPh), 3.71–3.56 (m, 2 H; OCH_2_CH_3_), 3.35–3.15 (m, 2 H; OCH_2_CH_3_), 2.54 (dd, ^3^*J*_HP_=2, 1 Hz, 3 H; =CCH_3_), 0.90 (dt, ^3^*J*_HH_=7, ^4^*J*_HP_=7 Hz, 3 H; OCH_2_CH_3_), 0.77 ppm (t, ^3^*J*_HH_=7 Hz, 3 H; OCH_2_CH_3_); ^13^C NMR (CDCl_3_): *δ*=198.0 (d, *J*=30 Hz), 194.3 (d, ^1^*J*_CW_=126, ^1^*J*_CP_=8 Hz), 170.2 (dd, *J*=32, 8 Hz), 141.6 (d, *J*=29 Hz), 137.1 (d, *J*=5 Hz), 130.5 (d, *J*=2 Hz), 129.2 (s), 129.1 (s), 128.7 (d, *J*=9 Hz), 128.2 (d, *J*=3 Hz), 126.2 (d, *J*=12 Hz), 105.7 (d, *J*=200 Hz), 61.5 (d, *J*=5 Hz), 61.1 (d, *J*=5 Hz), 60.8 (dd, *J*=10, 7 Hz), 16.3 (d, *J*=3 Hz), 15.9 (d, *J*=7 Hz), 15.5 ppm (d, *J*=8 Hz); IR (CH_2_Cl_2_): $\tilde \nu $
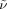
=2307, 2078, 1950, 1630, 1137 cm^−1^; HRMS (solution in MeOH/CHCl_3_): *m*/*z* calcd for C_25_H_24_O_9_P_2_WNa: 737.03026 [*M*+Na]^+^; found: 737.02821.

Oxaphospholes **3 d and 4 d**: Prepared from **1** (0.24 g, 0.43 mmol) and ketone **2 d** (0.09 g, 0.43 mmol). Compound **4 d** was obtained by filtration (0.09 g, 27 %) as a white solid after treating the crude mixture with pure diethyl ether. “Procedure” Chromatography of the residue by using CH_2_Cl_2_ (20 %) as eluent gave **3 d** (0.05 g, 18 %) as a white solid.

*Compound **3 d***: ^31^P NMR (121 MHz, CDCl_3_): *δ*=149.6 ppm (^1^*J*_PW_=289 Hz); ^1^H NMR (300 MHz, CDCl_3_): *δ*=7.84 (dd, ^3^*J*_HH_=8, ^4^*J*_HH_=2 Hz, 2 H; Ph), 7.60–7.33 (m, 13 H; Ph), 5.86 (dd, ^3^*J*_HP_=18, ^3^*J*_HH_=4 Hz, 1 H; HC=), 4.46 ppm (dd, ^2^*J*_HP_=5, ^3^*J*_HH_=4 Hz, 1 H; *H*CPh); ^13^C NMR (75 MHz, CDCl_3_): *δ*=198.5 (d, *J*=29 Hz), 194.8 (d, *J*=8 Hz), 158.4 (d, *J*=7 Hz), 142.3 (d, *J*=30 Hz), 138.0 (d, *J*=5 Hz), 130.3 (d, *J*=2 Hz), 129.7 (s), 129.3 (d, *J*=3 Hz), 129.1 (d, *J*=5 Hz), 128.8 (s), 128.7 (d, *J*=9 Hz), 128.2 (d, *J*=4 Hz), 126.4 (d, *J*=13 Hz), 125.6 (s), 102.5 (s), 60.1 ppm (d, *J*=11 Hz); IR (CH_2_Cl_2_): $\tilde \nu $
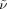
=2077, 1947, 1712, 1144, 1024 cm^−1^; HRMS (solution in MeOH/CHCl_3_ with addition of AgCF_3_COO): *m*/*z* calcd for C_26_H_17_O_6_PWAg: 748.93196 [*M*+Ag]^+^; found: 748.93386.

*Compound **4 d***: ^31^P NMR (121 MHz, CDCl_3_): *δ*=151.2 (d, ^1^*J*_PP_=28, ^1^*J*_PW_=294 Hz; P^III^), 14.8 ppm (d, ^1^*J*_PP_=28 Hz, P^V^); ^1^H NMR (300 MHz, CDCl_3_): *δ*=8.02 (dd, ^3^*J*_HH_=7, ^4^*J*_HH_=2 Hz, 2 H; Ph), 7.72–7.63 (m, 2 H; Ph), 7.59–7.50 (m, 6 H; Ph), 7.45–7.37 (m, 5 H; Ph), 4.72 (dd, *J*_HP_=6, 3 Hz, 1 H; *H*CPh), 3.63–3.48 (m, 2 H; OCH_2_CH_3_), 3.44–3.24 (m, 2 H; OCH_2_CH_3_), 0.90 (t, ^3^*J*_HH_=7 Hz, 3 H; OCH_2_CH_3_), 0.73 ppm (t, ^3^*J*_HH_=7 Hz, 3 H; OCH_2_CH_3_); ^13^C NMR (75 MHz, CDCl_3_): *δ*=198.3 (d, *J*=30 Hz), 194.7 (d, *J*=8 Hz), 166.7 (dd, *J*=28, 8 Hz), 141.6 (d, *J*=29 Hz), 137.4 (d, *J*=4 Hz), 131.6 (s), 130.9 (d, *J*=2 Hz), 130.1 (dd, *J*=3, 1 Hz), 129.8 (s), 129.6 (d *J*=3 Hz), 129.4 (d, *J*=6 Hz), 129.0 (d, *J*=9 Hz), 128.6 (d, *J*=3 Hz), 128.4 (s), 126.8 (d, *J*=12 Hz), 106.9 (d, *J*=200 Hz), 62.4 (dd, *J*=11, 7 Hz), 62.0 (d, *J*=5 Hz), 16.1 (d, *J*=7 Hz), 15.8 ppm (d, *J*=7 Hz); IR (CH_2_Cl_2_): $\tilde \nu $
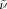
=2078, 1950, 1596, 1025 cm^−1^; HRMS (solution in MeOH/CHCl_3_ with addition of AgCF_3_COO): *m*/*z* calcd for C_30_H_27_O_6_PW: 777.06396 [*M*+H]^+^; found: 777.06253.

**Oxaphospholes 3 e and 4 e**: Prepared from **1** (0.14 g, 0.24 mmol) and ketone **2 e** (0.06 g, 0.24 mmol). Final compounds were obtained after chromatography by using CH_2_Cl_2_ (10 %) in pentane as eluent to give pure **3 e** (*R*_f_=0.37, 0.045 g, 28 %) as a white solid followed by 1:1 diethyl ether in pentane as eluent to give **4 e** (*R*_f_=0.26, 0.03 g, 15 %) as a pale-yellow oil.

*Compound **3 e***: ^31^P NMR (161 MHz, CDCl_3_): *δ*=132.8 ppm (^1^*J*_PW_=279 Hz); ^1^H NMR (400 MHz, CDCl_3_): *δ*=7.65 (d, ^3^*J*_HH_=7 Hz, 2 H; Ph), 7.43–7.28 (m, 8 H; Ph), 5.58 (dd, ^3^*J*_HP_=19, ^3^*J*_HH_=4 Hz, 1 H; HC=), 2.72 (dd, ^2^*J*_HP_=8, ^3^*J*_HH_=4 Hz, 1 H; *H*CTES), 1.04 (t, ^3^*J*_HH_=8 Hz, 9 H; SiCH_2_CH_3_), 0.96–0.83 ppm (m, 6 H; SiCH_2_CH_3_); ^13^C NMR (100 MHz, CDCl_3_): *δ*=198.5 (d, *J*=27 Hz), 196.0 (d, ^1^*J*_CW_=126, ^1^*J*_CP_=8 Hz), 155.1 (d, *J*=6 Hz), 145.9 (d, *J*=26 Hz), 130.8 (d, *J*=5 Hz), 129.6 (d, *J*=2 Hz), 128.8 (s), 128.7 (s), 128.3 (d, *J*=9 Hz), 125.4 (d, *J*=12 Hz), 124.8 (s), 101.9 (d, *J*=4 Hz), 40.4 (d, *J*=6 Hz), 7.6 (s), 3.3 ppm (d, *J*=2 Hz); HRMS (solution in MeOH/CHCl_3_): *m*/*z* calcd for C_26_H_28_O_6_PSiW: 679.09021 [*M*+H]^+^; found: 679.09109.

*Compound **4 e***: ^31^P NMR (161 MHz, CDCl_3_): *δ*=161.5 (d, ^1^*J*_PP_=36, ^1^*J*_PW_=281 Hz; P^III^), 16.0 (d, ^1^*J*_PP_=36 Hz; P^V^); ^1^H NMR (400 MHz, CDCl_3_): *δ*=7.82–7.79 (m, 4 H; Ph), 7.51–7.41 (m, 6 H; Ph), 4.01–3.88 (m, 2 H; OCH_2_CH_3_), 3.84–3.78 (m, 2 H; OCH_2_CH_3_), 3.64 (dd, *J*_HP_=5, 2 Hz, 1 H; *H*CTES), 1.09 (t, ^3^*J*_HH_=7 Hz, 3 H; OCH_2_CH_3_), 1.02 (t, ^3^*J*_HH_=7 Hz, 3 H; OCH_2_CH_3_), 0.84 (t, ^3^*J*_HH_=8 Hz, 9 H; SiCH_2_CH_3_), 0.51 (dq, ^3^*J*_HP_=16, ^3^*J*_HH_=8 Hz, 3 H; SiCH_2_CH_3_), 0.38 ppm (dq, ^3^*J*_HP_=16, ^3^*J*_HH_=8 Hz, 3 H; SiCH_2_CH_3_); ^13^C NMR (100 MHz, CDCl_3_): *δ*=198.8 (d, *J*=28 Hz), 196.1 (d, ^1^*J*_CW_=126, *J*_CP_=8 Hz), 162.2 (d, *J*=27 Hz), 137.4 (d, *J*=32 Hz), 132.3 (d, *J*=3 Hz), 131.7 (d, *J*=15 Hz), 131.3 (dd, *J*=6, 2 Hz), 130.7 (s), 129.5 (d, *J*=1 Hz), 128.3 (d, *J*=11 Hz), 128.0 (s), 103.5 (d, *J*=205 Hz), 62.3 (d, *J*=7 Hz), 61.4 (d, *J*=6 Hz), 46.0 (dd, *J*=10, 2 Hz), 15.8 (d, *J*=4 Hz), 15.7 (d, *J*=4 Hz), 7.6 (s), 3.6 ppm (d, *J*=2 Hz); HRMS (solution in MeOH/CHCl_3_): *m*/*z* calcd for C_30_H_37_O_9_P_2_SiW: 815.11914 [*M*+H]^+^; found: 815.11893.

**Reaction of 1 with ketone 2 f**: Compound **1** (0.31 g, 0.54 mmol, 1 equiv) was dissolved in THF (25 mL) and cooled to −20 °C. A solution of LDA (0.27 mL, 0.54 mmol, 1 equiv, 2 m in THF/heptanes) was added dropwise to the reaction mixture and stirred for another 20 min. After this time, it was cooled to −78 °C and a solution of ketone **2 f** (0.12 g, 0.54 mmol, 1 equiv) in THF (1 mL) was added dropwise. The reaction mixture was stirred for 30 min at the same temperature and subsequently methanol (0.2 mL) was added in one portion and the mixture was allowed to reach RT. Solvents were removed in vacuo and the residue was chromatographed on a silica gel column (*R*_f_=0.3, pentane) to give **6 f** (0.36 g, 66 %) as a mixture of two isomers in a 3:2 ratio. NMR data are given for the major isomer only (except ^31^P NMR). ^31^P NMR (161 MHz, C_6_D_6_): *δ*=134.8 (^1^*J*_PW_=287 Hz; major isomer), 134.3 ppm (^1^*J*_PW_=284 Hz; minor isomer); ^1^H NMR (400 MHz, C_6_D_6_): *δ*=7.67–7.63 (m, 2 H; Ph), 7.11–7.06 (m, 3 H; Ph), 3.08 (m, 1 H; HC), 3.00 (d, ^3^*J*_HP_=12 Hz, 3 H; OCH_3_), 1.27 (dd, ^3^*J*_HP_=15, ^3^*J*_HH_=7 Hz, 3 H; *H*CCH_3_), 1.10 ppm (br s, 21 H; TIPS); ^13^C NMR (100 MHz, C_6_D_6_): *δ*=198.4 (d, *J*=27 Hz), 196.6 (d, ^1^*J*_CW_=125, ^1^*J*_CP_=8 Hz), 136.8 (d, *J*=36 Hz), 131.0 (d, *J*=2 Hz), 130.4 (d, *J*=12 Hz), 128.4 (d, *J*=9 Hz), 105.5 (d, *J*=1 Hz), 87.7 (d, *J*=7 Hz), 54.2 (d, *J*=6 Hz), 34.4 (d, *J*=26 Hz), 19.6 (s), 16.0 (d, *J*=2 Hz), 11.4 ppm (d, *J*=1 Hz); HRMS (solution in MeOH): *m*/*z* calcd for C_25_H_33_O_6_PSiWaNa: 695.11910 [*M*+Na]^+^; found: 695.11898.

**Reaction between 1 and ketone 9**: Compound **1** (0.39 g (0.69 mmol, 1 equiv) was dissolved in THF (25 mL) and cooled to −20 °C. A solution of LDA (0.35 mL, 0.69 mmol, 1 equiv, 2 m in THF/heptanes) was added dropwise and the reaction mixture was stirred for 20 min at this temperature. After cooling to −78 °C, a solution of ketone **9** (0.27 g, 0.69 mmol, 1 equiv) in THF (1 mL) was added dropwise. The reaction mixture was stirred for 30 min at the same temperature and then 2,3-dimethylbuta-1,3-dyene (0.4 mL) was quickly added to the reaction mixture, which was then allowed to reach RT. All volatile compounds were removed in vacuo and the residue was chromatographed on a silica gel column (*R*_f_=0.6, pentane) to give **13** (0.22 g, 35 %) as a pale-yellow oil. ^31^P NMR (161 MHz, CDCl_3_): *δ*=9.7 ppm (^1^*J*_PW_=251 Hz); ^1^H NMR (400 MHz, CDCl_3_): *δ*=7.82–7.75 (m, 2 H; Ph), 7.39–7.33 (m, 3 H; Ph), 3.21 (dd, ^2^*J*_HH_=18, ^2^*J*_HP_=7 Hz; 1 H; CH_2_), 3.03 (d, ^2^*J*_HH_=17 Hz, 1 H; CH_2_), 2.75 (dd, ^2^*J*_HH_=18, ^2^*J*_HP_=7 Hz, 1 H; CH_2_), 2.43 (dd, ^2^*J*_HH_=17, ^3^*J*_HP_=17 Hz, 1 H; CH_2_), 1.83 (s, 3 H; CH_3_), 1.60 (s, 3 H; CH_3_), 1.09 (s, 21 H; TIPS), 0.97 ppm (s, 21 H; TIPS); ^13^C NMR (100 MHz, CDCl_3_): *δ*=199.0 (d, *J*=24 Hz), 196.4 (d, ^1^*J*_CW_=126, ^1^*J*_CP_=7 Hz), 135.6 (d, *J*=34 Hz), 130.0 (s), 129.9 (d, *J*=7 Hz), 128.1 (d. *J*=9 Hz), 125.4 (d, *J*=7 Hz), 121.1 (d, *J*=5 Hz), 105.4 (s), 104.7 (d, *J*=5 Hz), 86.9 (d, *J*=6 Hz), 85.6 (d, *J*=4 Hz), 44.8 (s), 34.8 (d, *J*=22 Hz), 34.2 (d, *J*=24 Hz), 21.3 (d, *J*=8 Hz), 20.0 (d, *J*=1 Hz), 18.5 (d, *J*=2 Hz), 18.4 (d, *J*=1 Hz), 11.2 (s), 11.1 ppm (s); IR (pentane): $\tilde \nu $
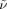
=2072, 1947, 1941, 1708 cm^−1^; HRMS (solution in MeOH/CHCl_3_ with addition of AgCF3COO): *m*/*z* calculated for C_40_H_57_O_5_PSi_2_Wag: 997.20392 [*M*+Ag]^+^; found: 995.20525.

**Reaction between 1 and ketone 11**: A solution of LDA (0.44 mL of a 2 m solution in THF/heptanes) was added dropwise at −30 °C to a solution of **1** (0.5 g; 0.88 mmol) in THF (50 mL). The reaction mixture was stirred for 30 min, then cooled to −78 °C. Ketone **11** (0.2 g; 0.88 mmol) was added and the resulting dark red-brown solution was stirred for 2 h at −20 °C. After this, water was added (50 mL) and the resulting suspension was stirred for 5 h at RT. The solvents were removed in vacuo and the residue was extracted with diethyl ether (3×50 mL). Combined organic fractions were dried over MgSO_4_. Removal of the solvent resulted in a crude red-brown product. Chromatographic purification on silica (2 % acetone in CH_2_Cl_2_; *R*_f_=0.6) afforded **17** (0.34 g, 49 %) as a red solid. ^31^P NMR (161 MHz, CD_2_Cl_2_): *δ*=37.2 (d, ^1^*J*_PP_=32, P^III^), 13.4 ppm (d, ^1^*J*_PP_=32 Hz, P^V^); ^1^H NMR (400 MHz, CD_2_Cl_2_): *δ*=11.5 (br s, 2 H; O*H*), 7.39–7.35 (m, 2 H; Ph), 7.28–7.24 (m, 8 H; Ph), 7.21–7.19 (m, 2 H; thiophene), 6.91–6.87 (m, 4 H; thiophene), 6.70–6.50 (br m, 2 H; thiophene), 6.25–5.80 (br m, 4 H; thiophene) 4.29–4.21 (m, 4 H; OCH_2_CH_3_), 4.09–4.00 (m, 2 H; OCH_2_CH_3_), 3.95–3.85 (m, 2 H; OCH_2_CH_3_), 1.41 (t, ^3^*J*_HH_=7 Hz, 6 H; OCH_2_CH_3_), 1.05 ppm (t, ^3^*J*_HH_=7 Hz, 6 H; OCH_2_CH_3_); ^13^C NMR (100 MHz, CD_2_Cl_2_): *δ*=198.1 (d, *J*=23 Hz), 196.4 (d, *J*_CP_=7 Hz, *J*_CW_=126 Hz), 161.4 (d, *J*=31 Hz), 155.4 (dd, *J*=23, 18 Hz), 133.6 (d, *J*=11 Hz), 133.5 (d, *J*=12 Hz), 131.0 (d, *J*=2 Hz), 130.0 (dd, *J*=5, 1 Hz), 128.4 (d, *J*=10 Hz), 128.3 (s), 128.3 (dd, *J*=166, 9 Hz), 126.6 (d, *J*=1 Hz), 124.8 (s), 118.8 (br m), 118.6 (br m), 63.8 (d, *J*=6 Hz), 63.5 (d, *J*=6 Hz), 15.9 (d, *J*=8 Hz), 15.7 ppm (d, *J*=7 Hz); IR (CH_2_Cl_2_): $\tilde \nu $
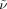
=3618, 2306, 1940, 1712, 1600, 1193, 1049 cm^−1^; HRMS (solution in ACN/CHCl_3_): *m*/*z* calcd for C_56_H_44_O_18_S_2_P_2_W_2_Na: 1646.92756 [*M*+Na]^+^; found: 1646.92837.

**Reaction of 1 and ketones 18 and 19**: Compound **1** (1 equiv) was dissolved in THF (5–15 mL) and cooled to −20 °C. A solution of LDA (1 equiv, 2 m in THF/heptanes) was added dropwise and the reaction mixture was stirred for 20 min. After cooling to −78 °C, a solution of the ketone (1 equiv) in THF was added dropwise. The reaction mixture was stirred for 1.5 h at −50 °C. For compounds **20** and **22**: the reaction mixture was quenched with water (0.5 mL) at −50 °C and allowed to warm to RT. Products were extracted with diethyl ether, washed with brine and concentrated in vacuo. Crude products were chromatographed by using CH_2_Cl_2_ (20 %) in pentane as eluent to give **20** (*R*_f_=0.68) as a white solid and **22** (*R*_f_=0.8) as a colorless oil. For compounds **21** and **23**: the reaction mixture was directly poured onto a silica plug and washed with THF. After removal of the solvent in vacuo the residue was subjected to column chromatography by using diethyl ether/pentane (1:1) as eluent (*R*_f_=0.5) to give final products **21** and **23** as white solids.

*Compound **20***: Reaction was performed by using **1** (0.18 g, 0.32 mmol) and **18** (0.1 g, 0.32 mmol) to give **20** (0.11 g, 37 %). ^31^P NMR (121 MHz, CDCl_3_): *δ*=150.0 ppm (s_satellite_, ^1^*J*_PW_=292 Hz); ^1^H NMR (300, CDCl_3_): *δ*=7.62–7.51 (m, 4 H; Ph), 7.47–7.38 (m, 4 H; Ph), 7.32–7.28 (m, 2 H; Ph), 5.72 (dd, ^3^*J*_HP_=17, ^3^*J*_HH_=4 Hz, 1 H; HC=), 4.26 (dd, ^2^*J*_HP_=4, ^3^*J*_HH_=4 Hz, 1 H; *H*CPh), 1.18 ppm (br s, 21 H; TIPS); ^13^C NMR (75 MHz, CDCl_3_): *δ*=198.3 (d, *J*=30 Hz), 194.5 (d, ^1^*J*_WC_=126, ^1^*J*_CP_=8 Hz), 142.4 (d, *J*=6 Hz), 142.0 (d, *J*=30 Hz), 137.0 (d, *J*=5 Hz), 130.4 (d, *J*=2 Hz), 129.4 (d, *J*=3 Hz), 129.0 (d, *J*=5 Hz), 128.7 (d, *J*=9 Hz), 128.4 (d, *J*=4 Hz), 126.7 (d, *J*=12 Hz), 114.4 (s), 98.2 (s), 96.2 (d, *J*=5 Hz), 59.9 (d, *J*=10 Hz), 18.6 (s), 11.2 ppm (s); IR (CH_2_Cl_2_): $\tilde \nu $
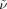
=2078, 1949, 1603, 1200, 1123 cm^−1^; HRMS (solution in CH_3_CN/CHCl_3_ with addition of AgCF_3_COO): *m*/*z* calcd for C_31_H_33_O_6_PSiWAg: 853.03549 [*M*+Ag]^+^; found: 853.03577.

*Compound **21***: Reaction was carried out by using **1** (0.18 g, 0.32 mmol) and **18** (0.1 g, 0.32 mmol) to give **21** (84 mg, 30 %). ^31^P NMR (121 MHz, C_6_D_6_): *δ*=156.0 (d, ^1^*J*_PW_=295, ^1^*J*_PP_=27 Hz; P^III^), 12.4 ppm (d, ^1^*J*_PP_=27 Hz); ^1^H NMR (300 MHz, CDCl_3_): *δ*=7.61–7.48 (m, 4 H; Ph), 7.46–7.36 (m, 4 H; Ph), 7.33–7.28 (m, 2 H; Ph), 4.44 (dd, *J*_HP_=6, *J*_HP_=3 Hz, 1 H; *H*CPh), 3.73–3.63 (m, 2 H; OCH_2_CH_3_), 3.56–3.36 (m, 2 H; OCH_2_CH_3_), 1.20 (br s, 21 H; TIPS), 1.00 (t, ^3^*J*_HH_=7 Hz, 3 H; OCH_2_CH_3_), 0.88 ppm (t, ^3^*J*_HH_=7 Hz, 3 H; OCH_2_CH_3_); ^13^C NMR (75 MHz, CDCl_3_): *δ*=197.9 (d, *J*=30 Hz), 194.2 (d, ^1^*J*_CW_=124, ^1^*J*_CP_=9 Hz), 148.9 (dd, *J*=25, 7 Hz), 141.0 (d, *J*=29 Hz), 136.1 (d, *J*=4 Hz), 130.6 (d, *J*=2 Hz), 129.3 (d, *J*=3 Hz), 129.1 (d, *J*=5 Hz), 128.7 (d, *J*=9 Hz), 128.5 (d, *J*=3 Hz), 126.7 (d, *J*=12 Hz), 116.1 (d, *J*=196 Hz), 105.2 (s), 95.1 (dd, *J*=5, 3 Hz), 61.9 (d, *J*=5 Hz), 61.8 (d, *J*=5 Hz), 61.7 (dd, *J*=9, 7 Hz), 18.5 (s), 15.9 (d, *J*=7 Hz), 15.8 (d, *J*=7 Hz), 11.2 ppm (s); HRMS (solution in MeOH/CHCl_3_): *m*/*z* calcd for C_35_H_42_O_9_PSiWNa: 903.14749 [*M*+H]^+^; found: 903.14884.

*Compound **22***: Reaction was performed by using **1** (0.11 g, 0.2 mmol) and **19** (0.05 g, 0.2 mmol). Compound **21** (15 mg, ca. 10 %) appears to be unstable upon chromatography and cannot be isolated in pure form. ^31^P NMR (121 MHz, CDCl_3_): *δ*=150.1 ppm (^1^*J*_WP_=292 Hz); ^1^H NMR (300 MHz, CDCl_3_): *δ*=7.63–7.41 (m, 8 H; Ph), 7.31–7.28 (m, 2 H; Ph), 5.73 (dd, ^3^*J*_HP_=17, ^3^*J*_HH_=4 Hz, 1 H; HC=), 4.28 (dd, ^2^*J*_HP_=4, ^3^*J*_HH_=4 Hz, 1 H; *H*CPh), 1.09 (t, ^3^*J*_HH_=8 Hz, 9 H; SiCH_2_CH_3_), 0.91–0.63 ppm (m, 6 H; SiCH_2_CH_3_); ^13^C NMR (75 MHz, CDCl_3_): *δ*=198.2 (d, *J*=30 Hz), 194.5 (d, ^1^*J*_CW_=126, ^1^*J*_CP_=8 Hz), 142.5 (d, *J*=6 Hz), 141.9 (d, *J*=30 Hz), 137.0 (d, *J*=5 Hz), 130.4 (d, *J*=2 Hz), 129.3 (d, *J*=3 Hz), 129.0 (d, *J*=5 Hz), 128.7 (d, *J*=7 Hz), 128.3 (d, *J*=4 Hz), 126.6 (d, *J*=12 Hz), 114.7 (s), 98.9 (s), 95.3 (d, *J*=6 Hz), 59.8 (d, 10 Hz), 7.4 (s), 4.0 ppm (s); HRMS (solution in ACN/CHCl_3_ with addition of AgCF_3_COO): *m*/*z* calcd for C_28_H_27_O_6_PSiWAg: 810.98850 [*M*+Ag]^+^; found: 810.98942.

*Compound **23***: Reaction was performed by using **1** (0.1 g, 0.18 mmol) and **19** (0.05 g, 0.18 mmol) to form **23** (39 mg, 27 %). ^31^P NMR (121 MHz, CD_2_Cl_2_): *δ*=156.0 (d, ^1^*J*_PP_=27 Hz; P^III^), 11.7 ppm (d, ^1^*J*_PP_=27 Hz; P^V^); ^1^H NMR (300 MHz, CD_2_Cl_2_): *δ*=7.64–7.54 (m, 4 H; Ph), 7.51–7.48 (m, 1 H; Ph), 7.44–7.39 (m, 3 H; Ph), 7.33–7.29 (m, 2 H; Ph), 4.66 (dd, *J*_HP_=6, 3 Hz, 1 H; *H*CPh), 3.74–3.35 (m, 4 H; OCH_2_CH_3_), 1.10 (t, ^3^*J*_HH_=8 Hz, 6 H; OCH_2_CH_3_), 1.03 (t, ^3^*J*_HH_=7 Hz, 3 H; SiCH_2_CH_3_), 0.91 (t, ^3^*J*_HH_=7 Hz, 3 H; SiCH_2_CH_3_), 0.82–0.73 ppm (m, 6 H; SiCH_2_CH_3_); ^13^C NMR (75 MHz, CD_2_Cl_2_): *δ*=198.2 (d, *J*=30 Hz), 194.5 (d, *J*=8 Hz), 140.9 (d, *J*=30 Hz), 136.5 (d, *J*=6 Hz), 130.9 (d, *J*=2 Hz), 129.5 (d, *J*=3 Hz), 129.3 (d, *J*=6 Hz), 129.0 (d, *J*=9 Hz), 128.7 (d, *J*=3 Hz), 126.9 (d, *J*=12 Hz), 116.9 (d, *J*=195 Hz), 105.9 (s), 94.5 (dd, *J*=5, 3 Hz), 61.8 (dd, *J*=9, 7 Hz), 62.2 (d, *J*=5 Hz), 62.1 (d, *J*=5 Hz), 16.0 (d, *J*=7 Hz), 15.9 (d, *J*=7 Hz), 7.34 (s), 4.1 ppm (s); IR (CH_2_Cl_2_): $\tilde \nu $
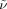
=2079, 1951, 1605, 1290, 1047 cm^−1^; HRMS (solution in MeOH/CHCl_3_): *m*/*z* calcd for C_32_H_37_O_9_P_2_SiW: 839.11859 [*M*+H]^+^; found: 839.12016.

**One-pot reaction of 1 with 19 and 8**: Compound **1** (0.1 g, 0.18 mmol) was dissolved in THF (10 mL) and the solution was cooled to −78 °C. After this, a solution of BuLi (0.1 mL, 1 equiv, 1.6 m in hexane) was added dropwise to the reaction mixture and stirred for 30 min. Subsequently, a solution of ketone **19** (0.047 g, 0.18 mmol) in THF (1 mL) was added dropwise and the reaction mixture was stirred for 1 h at −50 °C. After this, a solution of ketone **8** (0.054 g, 0.18 mmol) in THF (1 mL) was added. The reaction mixture was stirred for 30 min at −50 °C and then warmed to RT within 30 min. The reaction mixture was directly applied onto a silica gel plug and washed with THF. After removal of solvents in vacuo, the residue was chromatographed by using CH_2_Cl_2_ as eluent (*R*_f_=0.51) to give cumulene (31 mg, 17 %) as a bright-red amorphous solid.

*Cumulene **25***: ^31^P NMR (161 MHz, CD_2_Cl_2_): *δ*=150.5 (d, ^1^*J*_PP_=43, ^1^*J*_PW_=297 Hz, P^III^), 5.8 ppm (d, ^1^*J*_PP_=43 Hz); ^1^H NMR (400, CD_2_Cl_2_): *δ*=7.74–7.65 (m, 5 H; Ph), 7.40–7.30 (m, 3 H; Ph), 7.06–7.04 (m, 2 H; Ph), 4.17–3.97 (m, 4 H; OCH_2_CH_3_), 1.24 (t, ^3^*J*_HH_=7 Hz, 3 H; OCH_2_CH_3_), 1.21 (t, ^3^*J*_HH_=7 Hz, 3 H; OCH_2_CH_3_), 1.04 (t, ^3^*J*_HH_=8 Hz, 9 H; SiCH_2_CH_3_), 1.03 (t, ^3^*J*_HH_=8 Hz, 9 H; SiCH_2_CH_3_), 0.72–0.64 ppm (m, 12 H; SiCH_2_CH_3_); ^13^C NMR (100 MHz, CD_2_Cl_2_): *δ*=197.7 (d, *J*=30 Hz), 194.6 (d, ^1^*J*_CW_=126, ^1^*J*_CP_=8 Hz), 161.9 (dd, *J*=17, *J*=5 Hz), 157.9 (s), 142.9 (s), 142.6 (s), 135.5 (d, *J*=32 Hz), 133.3 (d, *J*=2 Hz), 131.5 (dd, *J*=13, 4 Hz), 130.8 (d, *J*=16 Hz), 130.2 (d, *J*=2 Hz), 130.0 (dd, *J*=178, 6 Hz), 129.6 (d, *J*=11 Hz), 129.1 (d, *J*=5 Hz), 128.4 (s), 101.9 (s), 101.5 (s), 100.9 (s), 63.4 (d, *J*=5 Hz), 63.3 (d, *J*=5 Hz), 7.4 (s), 4.4 ppm (s); IR (CH_2_Cl_2_): $\tilde \nu $
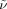
=2077, 1946, 1606, 1210, 1048 cm^−1^; HRMS (solution in ACN/CHCl_3_): *m*/*z* calcd for C_43_H_51_O_9_P_2_Si_2_W: 1013.20548 [*M*+H]^+^; found: 1013.20612.
