# Supplementary material for: Oxaphospholes and Bisphospholes from Phosphinophosphonates and α,β-Unsaturated Ketones
Source: Chemistry. 2013 Aug 26;19(41):13692–704. doi: 10.1002/chem.201302014 (PMC4304286; doi:10.1002/chem.201302014)
Supplement: Supplementary file 1 [file chem0019-13692-SD1.pdf]

CHEMISTRY
A EUROPEAN JOURNAL

Supporting Information

© Copyright Wiley-VCH Verlag GmbH & Co. KGaA, 69451 Weinheim, 2013

Oxaphospholes and Bisphospholes from Phosphinophosphonates and α,β-Unsaturated Ketones

Anna I. Arkhypchuk,[a] Andreas Orthaber,[a] Viorica Alina Mihali,[a] Andreas Ehlers,[b] Koop Lammertsma,[b] and Sascha Ott*[a]

chem_201302014_sm_miscellaneous_information.pdf

Electronic supporting information

Table of content

Experimental Section 2

Crystallographic data for complex 3e and 21 3

Preparation of the ketones 2a-f: 3

Shemical shifts and coupling constants for ring carbons and protons of 3a-e, 20 and 22 6

Shemical shifts and coupling constants for ring carbons and protons of 4c-e, 21 and 23 6

Shemical shifts and coupling constants for ring carbons and protons of 14, 15 and 25 6

NMR data 7

CV data for compounds 15 and 25 32

CV data for compounds 16 and 17 32

UV/Vis data for compounds 17 33

UV/Vis data for compounds 25 33

Computational Section 34

References 49

Experimental Section

General. All reactions were performed under argon using Schlenk techniques. Diethyl ether and THF were freshly distilled from sodium/benzophenone prior to use. ^{1}H, ^{13}C and ^{31}P spectra were recorded on spectrometers operating at proton frequencies of 400 MHz or 300 MHz. Chemical shifts are reported in ppm and referenced internally to residual solvent signals (^{1}H, ^{13}C) or externally to 85% $H_3PO_{4(aq)}$ (^{31}P).High resolution mass spectral analyses (HRMS) were performed on high resolution and FTMS+pNSI mass spectrometer (OrbitrapXL).

X-ray data. Crystallographic data sets were collected from single crystal samples mounted on a loop fiber and coated with N-paratone oil (Hampton Research). Crystal handling turned out to be different for the two compounds: while the crystals of **4** are stable once obtained and can be kept as solid blocks, the crystals of **3** (grown from slow evaporation of a pentane solution at 243K) melt at temperatures above 270K and required careful mounting at low temperatures (below 260-270K) to limit fast degradation of the crystals. We tried as best as we could: several data sets were collected for **4**, all solutions could reveal the same atom connectivity shown here and only the best data set resolution is here reported (one that shows the most intense and define diffraction; however no diffraction at high angles could be observed).

Crystallographic data sets were collected from single crystal samples mounted on a loop fiber and coated with N-paratone oil (Hampton Research). Collection was performed using a Bruker SMART APEX diffractometer equiped with an APEXII CCD detector, a graphite monochromator and a 3-circles goniometer. The crystal-to-detector distance was 5.0 cm, and the data collection was carried out in 512 x 512 pixel mode. The initial unit cell parameters were determined by a least-squares fit of the angular setting of strong reflections, collected by a 10.0 degrees scan in 33 frames over three different parts of the reciprocal space (99 frames total). Cell refinement and data reduction were performed with SAINT V7.68A (Bruker AXS). Absorption correction was done by multi-scan methods using SADABS96 (Sheldrick). The structure was solved by direct methods and refined using SHELXL97 (Sheldrick). All non-H atoms were

refined by full-matrix least-squares with anisotropic displacement parameters while hydrogen atoms were placed in idealized positions. Refinement of F2 was performed against all reflections. The weighted R-factor wR and goodness of fit S are based on F2. Full details concerning the data sets and crystal resolutions can be found in the respective CIF files deposited at the Cambridge Crystallographic Data Centre under the allocated deposition numbers CCDC 848398 (**3e**) and CCDC 933297 (**21**).

Crystallographic data for complex 3e and 21

Table SI1. Crystallographic data for complex **3e and 21**.

Compound	**3e**	**21**
Formula	$C_{26}H_{27}O_6PSiW$	$C_{35}H_{42}O_9P_2SiW$
M_w(g/mol); F(000)	678.39; 668	880.56 ; 884
T(K); wavelength (Å)	100; 0.71073	100(2) ; 0.71073
Crystal System	Triclinic	Triclinic
Space group	P1	P-1
Unit Cell: *a* (Å)	10.0541(6)	11.3593(9)
b (Å)	10.7550(6)	12.8431(10)
c (Å)	13.3048(8)	14.1496(11)
α (°)	99.628(1)	80.4220(10)
β (°)	95.379(1)	73.8380(10)
γ (Å)	105.907(1)	79.6320(10)
V(Å^3); Z; d_{calc} (g/cm^3)	1349.23(14); 2; 1.670	1935.5(3); 2; 1.511
θ range(°); completeness	1.57 to 30.03; 0.983	1.62 to 28.33; 0.991
Collected reflections; R_s	25480; 0.0374	23873; 0.0449
Unique reflections; R_{int}	12733; 0.024	9556; 0.0400
μ(mm^{-1}); Abs. Corr.	4.422; Semi-empirical from equivalents	3.147, multi-scan<br>T_{min} 0.6241; T_{max} 0.7457
$R_1(F)$;$wR(F^2)$ [I>2s(I)]	0.0353; 0.0888	0.0308; 0.0772
$R_1(F)$;$wR(F^2)$ (all data)	0.0420; 0.0959	0.0336; 0.0810
Parameters; Restraints	580;27	438; 8
GoF(F^2)	1.045	1.051
Residual electron density (e^-/Å^3)	2.239; -3.003	1.568; -1.567

Preparation of the ketones 2a-f:

2a. Ketone **2a** was prepared following a procedure of H.D. Verkruijsse at. al. [1]. NMR data are consistent with literature [2].

2b.

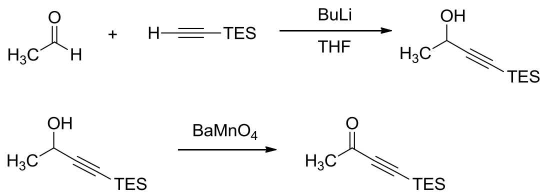

Synthesis of the alcohol: to the solution of 1.17g (8.4mmol) of TES-acetylene in 25ml THF at -50°C, solution of n-BuLi in hexane (3.4 ml, 2.5M) was added drop wise. The reaction mixture was stirred for 1 h and 0.34 g (8.4 mmol) of acetaldehyde was added at ones. Reaction mixture allowed to warm up to room temperature (r.t.) and quenched with sat. aqueous ammonium chloride solution. After 30 min reaction mixture was extracted 3 times with 50 ml diethyl ether, washed with brine and dried over $MgSO_4$. Solvents were removed *in vacuo* giving clean alcohol, which was used in next step without additional purification. Yield: 1.43 g, 92%. NMR data are consistent with literature [2].

Oxidation with $BaMnO_4$: to the solution of 1.4 g (7.7 mmol) of alkohol in 20 ml of dry CH_2Cl_2 3.6 g (14 mmol, 1.8 eq.) of barium manganate was added at once. The resulting suspension was stirred oven night at room temperature. Then the reaction mixture was filtered through a plug of silica, and the solvent removed in vacuum giving **2b** as pale yellow oil. Yield: 0.74 g, 53%. NMR data are consistent with literature [2].

2c. Ketone **2c** was prepared by oxidation of the corresponding alcohol (prepared by procedure of C. J. Tailor at. al. [3])by $BaMnO_4$ (see description for **2b**). NMR data are consistent with literature [4].

2d. Ketone **2d** was prepared following a procedure of She at. al., NMR data are consistent with literature [5].

2e. Ketone **2e** was prepared by the same procedure as **2b**. NMR data for alcohol and ketone are consistent with the literature [6].

2f. Ketone **2f** was prepared following a procedure of O. Klein at. al [7]. NMR data are consistent with literature [8].

7. Ketone **7** was prepared by procedure of T. Lange at. al. [9]. Oxidation of the corresponding alcohol was done by BaMnO4 (see preparation of 2b). NMR data of ketone 7 and corresponding alcohol are consistent with literature [9].

8. Ketone **8** was prepared following a literature procedure. NMR data for alcohol and ketone are consistent with the literature [10], [11].

9. Ketone **9** was prepared following a literature procedure. NMR data for alcohol and ketone are consistent with the literature [10], [11].

10. Ketone **10** was prepared following a literature procedure. NMR data for alcohol and ketone are consistent with the literature [10], [11].

11. Ketone **11** was prepared following a literature procedure. NMR data for alcohol and ketone are consistent with the literature [12].

18. Ketone **18** was prepared following a literature procedure. Oxidation of the corresponding alcohol was done by BaMnO4 (see preparation of **2b**). NMR data for alcohol and ketone are consistent with the literature [13].

19. Ketone **19** was prepared following a literature procedure[13]. Oxidation of the corresponding alcohol was done by BaMnO4 (see preparation of **2b**).

Alkohol: Yield: 76%. Pale yellow oil. H1 ($CDCl_3$): δ = 7.50-7.47 (m, 2H, Ph), 7.36-7.32 (m, 3H, Ph), 5.36 (bs, 1H, CH), 2.39 (bs, 1H, OH), 1.04 (t, $^3J_{HH}$=8 Hz, 9H, $SiCH_2C\underline{H}_3$), 0.68 (q, $^3J_{HH}$=8 Hz, 6H, $SiC\underline{H}_2CH_3$) ppm.

Ketone: Yield: 99 %. Pale yellow oil. ^1H ($CDCl_3$): δ = 7.65-7.62 (m, 2H, Ph), 7.51-7.39 (m, 3H, Ph), 1.07 (t, $^3J_{HH}$=8 Hz, 9H, $SiCH_2C\underline{H}_3$),0.72 (q, $^3J_{HH}$=8 Hz, 6H, $SiC\underline{H}_2CH_3$) ppm. ^{13}C ($CDCl_3$): δ = 160.7 (s), 133.6 (s), 131.5 (s), 128.9 (s), 119.7 (s), 104.3 (s), 98.2 (s), 91.8 (s), 89.7 (s), 7.5 (s), 4.0 (s). HRMS (solution in MeOH/$CHCl_3$): calc. for $C_{17}H_{20}SiONa$ 291.11756 $[M+Na]^+$, found 291.11757.

Shemical shifts and coupling constants for ring carbons and protons of 3a-e, 20 and 22

Table SI2. Shemical shifts and coupling constants for ring carbons and protons of heterocycles 3a-e, 20 and 22.

**Comp.**	**δ(C^3)**	**$^1J(C^3P)$**	**δ(C^4)**	**$^2J(C^4P)$**	**δ(C^5)**	**$^2J(C^5P)$**	**δ(H^3)**	**$^2J(H^3P)$**	**δ(H^4)**	**$^3J(H^4P)$**
1	2	3	4	5	6	7	8	9	10	11
**3a**	42.5	4	101.9	3	154.8	7	2.36	8	4.81	18
**3b**	39.2	5	102.0	3	154.4	6	2.35	7	4.34	19
**3c**	59.8	11	103.7		158.3	7	4.24	5[a]	5.09	18
**3d**	60.1	11	102.5		158.4	7	4.46	5	5.86	18
**3e**	40.4	6	101.9	4	155.1	6	2.72	8	5.58	19
**20**	59.9	10	114.4		142.4	6	4.26	4	5.72	17
**22**	59.8	10	114.7		142.5	6	4.28	4	5.73	17

[a] Coupling constants were resolved by applying enhanced resolution work up method to the original spectra.

Shemical shifts and coupling constants for ring carbons and protons of 4c-e, 21 and 23

Table SI3. Shemical shifts and coupling constants for ring carbons and protons of heterocycles 4c-e, 21 and 23.

**Comp.**	**δ(C^3)**	**$^1J(C^3P^V)$**	**$^1J(C^3P^{III})$**	**δ(C^4)**	**$^1J(C^4P^V)$**	**δ(C^5)**	**$^1J(C^5P^V)$**	**$^1J(C^5P^{III})$**	**δ(H^3)**	**$^3J(H^3P^V)$**	**$^2J(H^3P^{III})$**
1	2	3	4	5	6	7	8	9	10	11	12
**4c**	60.8	10	7	105.7	200	170.2	32	8	4.25	4	4
**4d**	62.4	11	7	106.9	200	166.7	28	8	4.72	3	6
**4e**	46.0	10	2	103.5	205	162.2	27		3.64	2	5
**21**	61.7	9	7	116.1	196	148.9	25	7	4.44	3	6
**23**	61.8	9	7	116.9	195	140.9	30	2	4.66	3	6

Shemical shifts and coupling constants for ring carbons and protons of 14, 15 and 25

Table SI4. Shemical shifts and coupling constants for ring carbons and protons of cumulenes 14, 15 and 25.

**Comp.**	**δ(C^3)**	**$^1J(C^3P^V)$**	**$^1J(C^3P^{III})$**	**δ(C^4)**	**$^1J(C^4P^V)$**	**$^2J(C^4P^{III})$**	**δ(C^5)**	**$^1J(C^5P^V)$**	**$^1J(C^5P^{III})$**	**δ(P^{III})**	**δ(P^V)**	**$^1J(PP)$**
1	2	3	4	5	6	7	8	9	10	11	12	13
14	129.2	11		143.5	189		169.1	19	19	168.2	6.4	59
15	129.0	11		143.0	188		168.9	22	18	167.5	6.8	63
25	131.5	13	4	130.0	178	6	161.9	17	5	150.5	5.8	43
24										150.7	6.0	43

NMR data

^{1}H NMR of complex **3a**

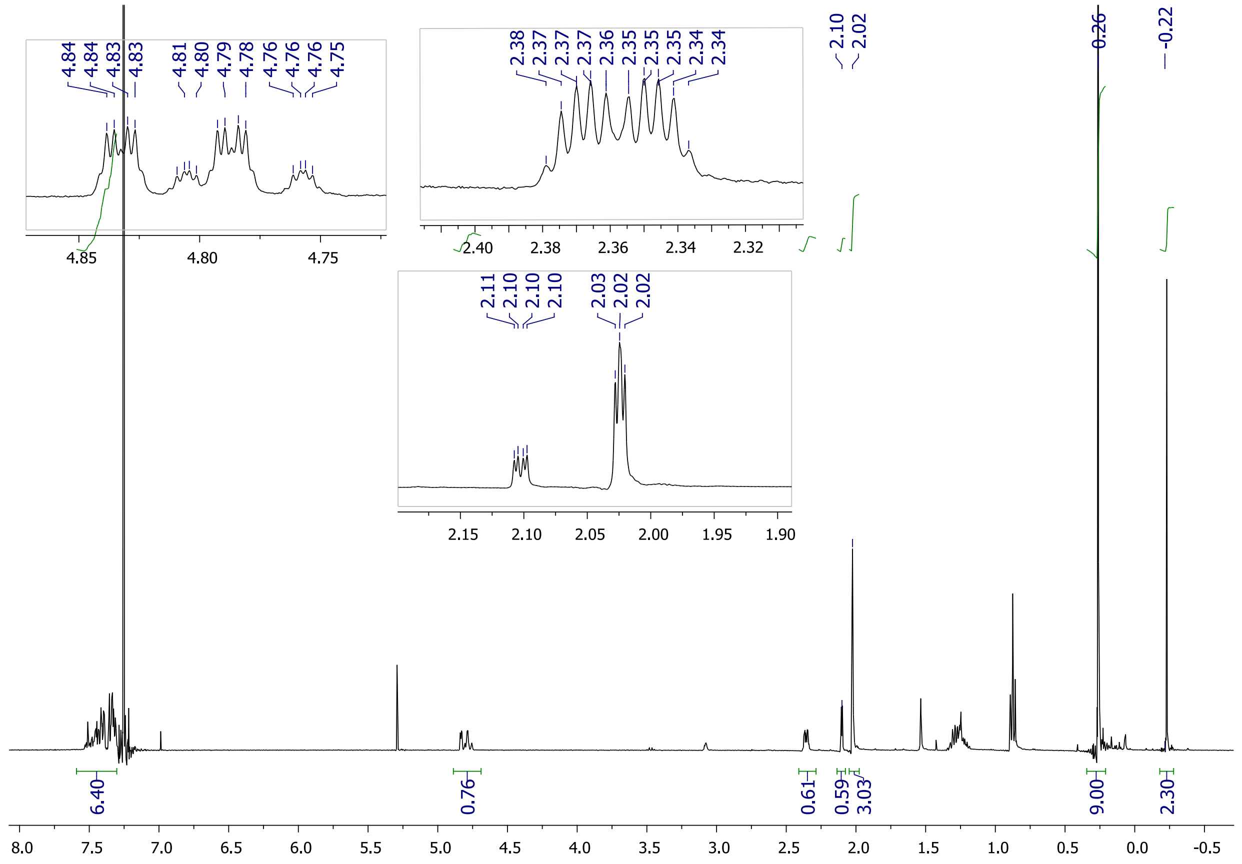

^{31}P NMR of complex **3a**

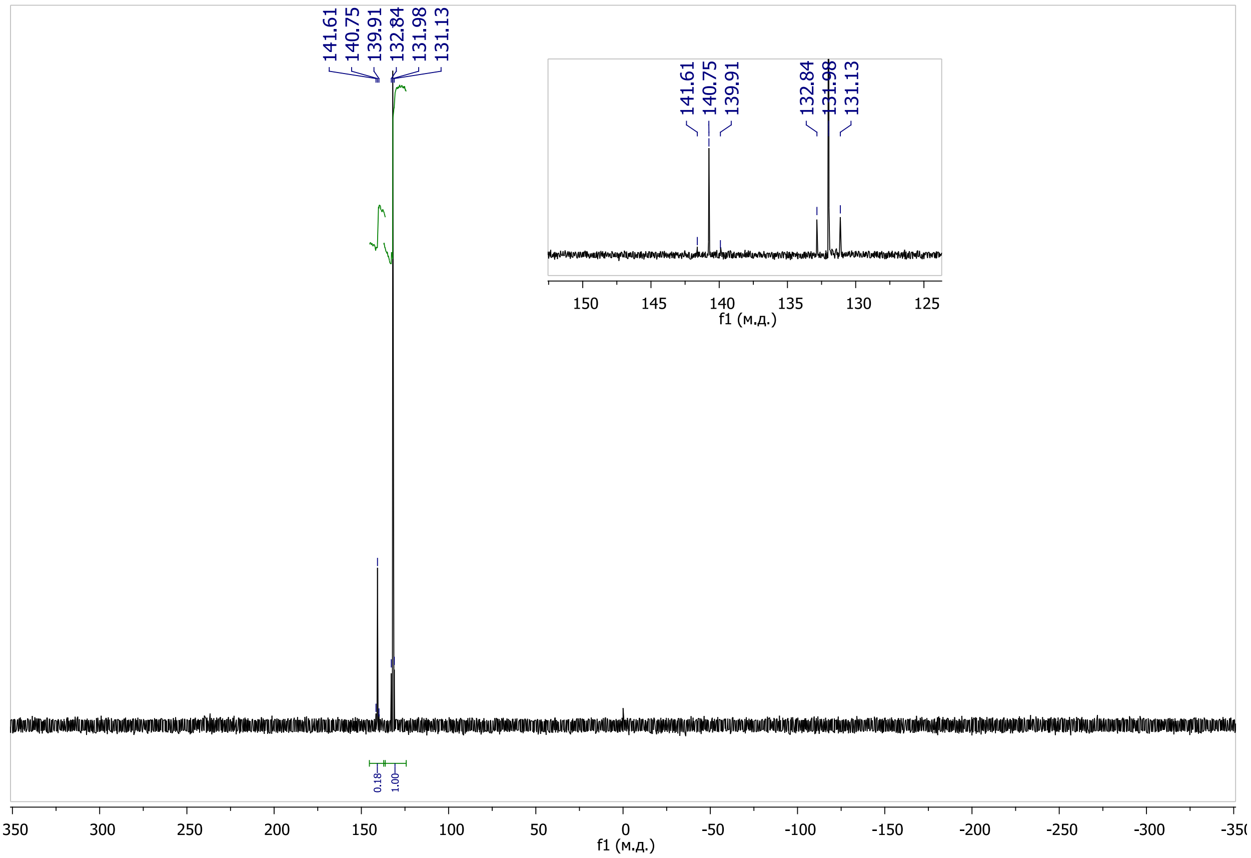

^{13}C NMR of complex **3a**

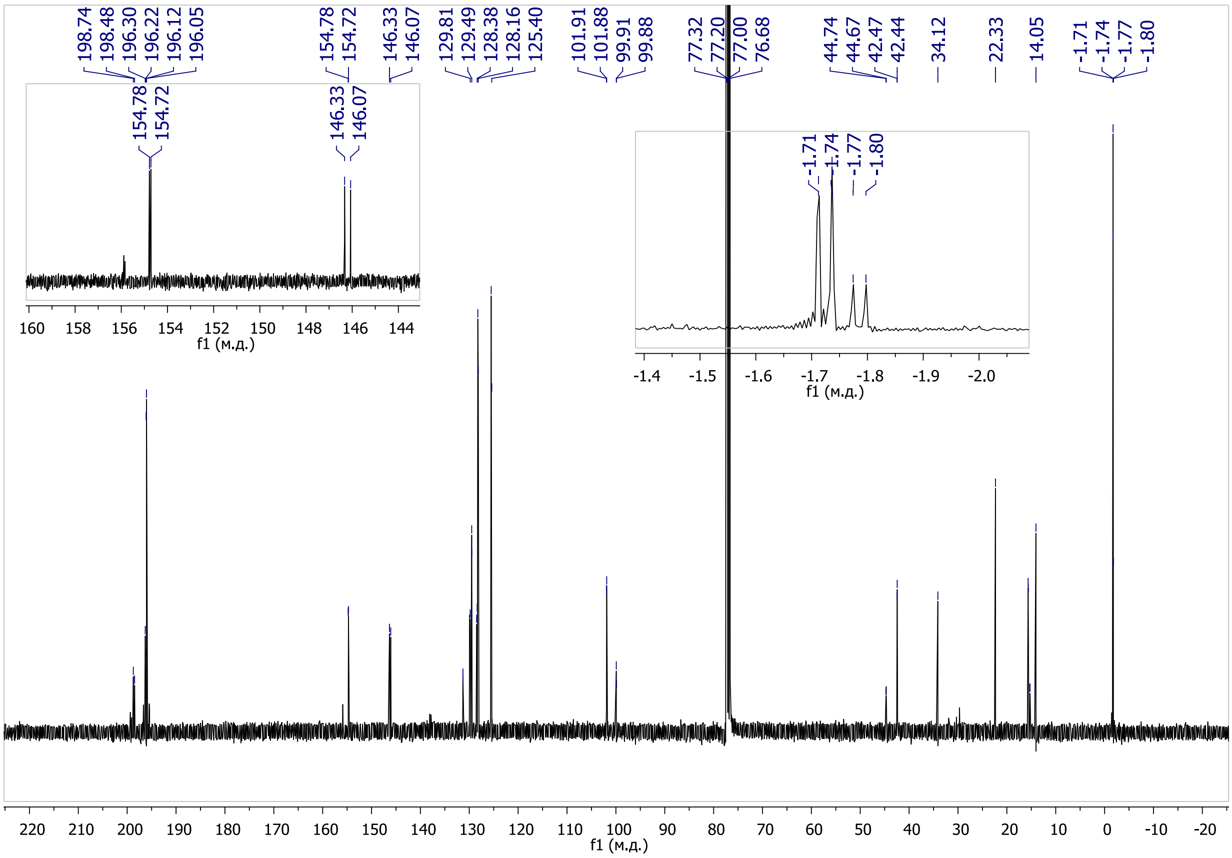

^{1}H NMR of complex **3b**

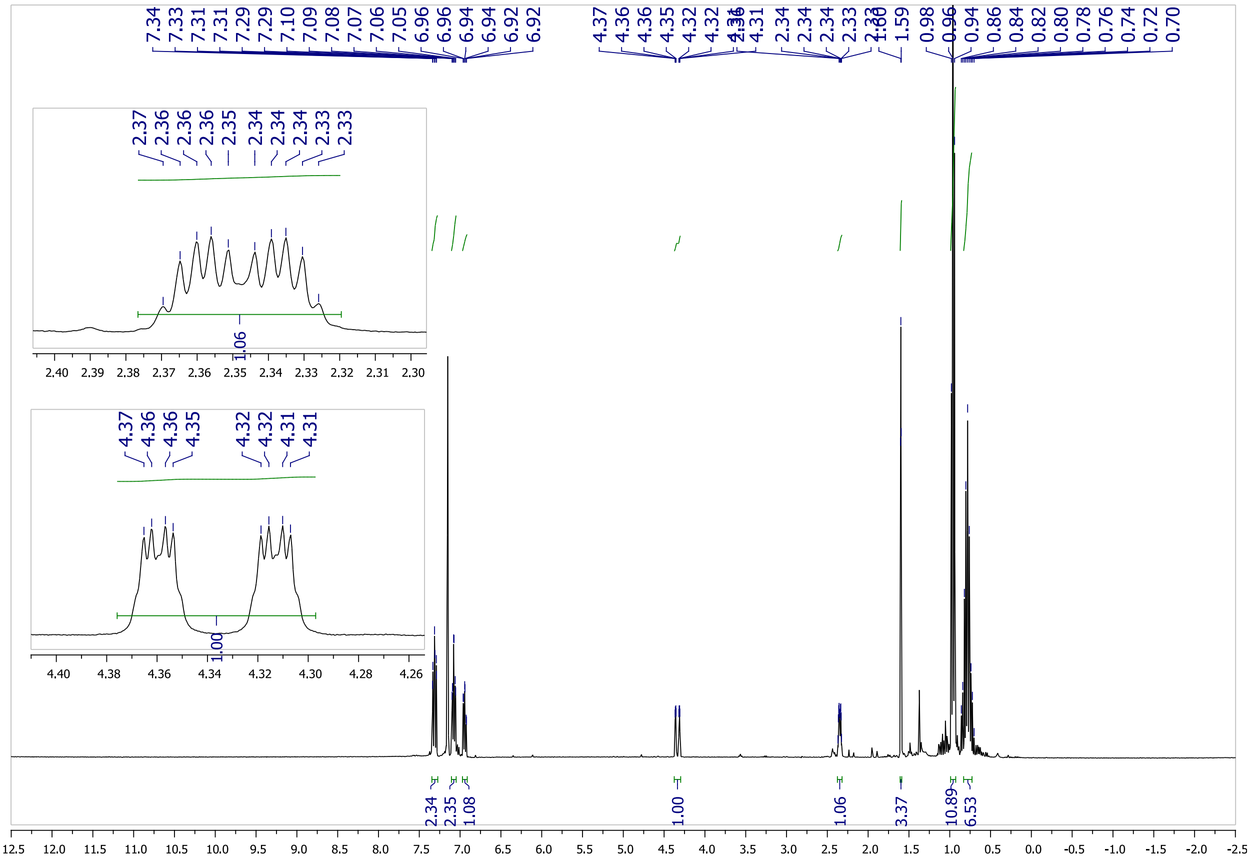

$^{1}H - ^{1}H$ COSY NMR of complex **3b**

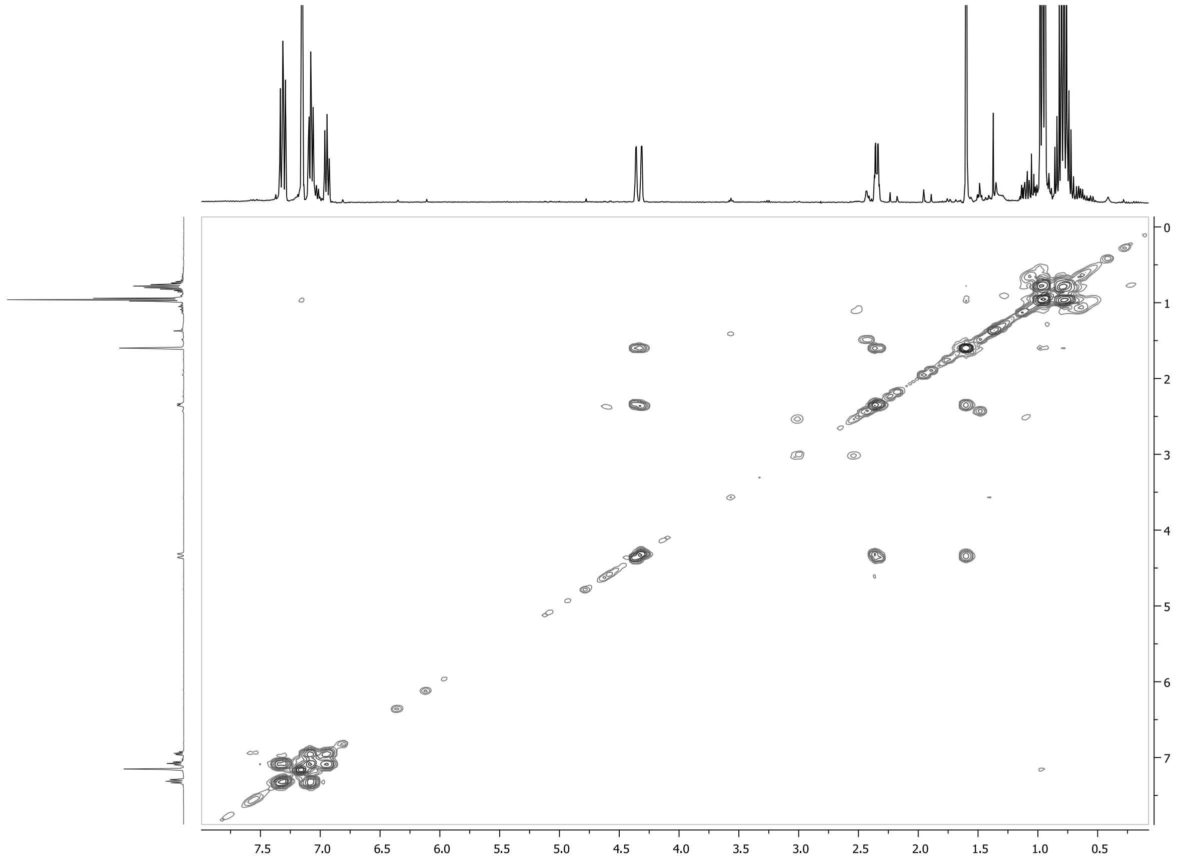

^{31}P NMR of complex **3b**

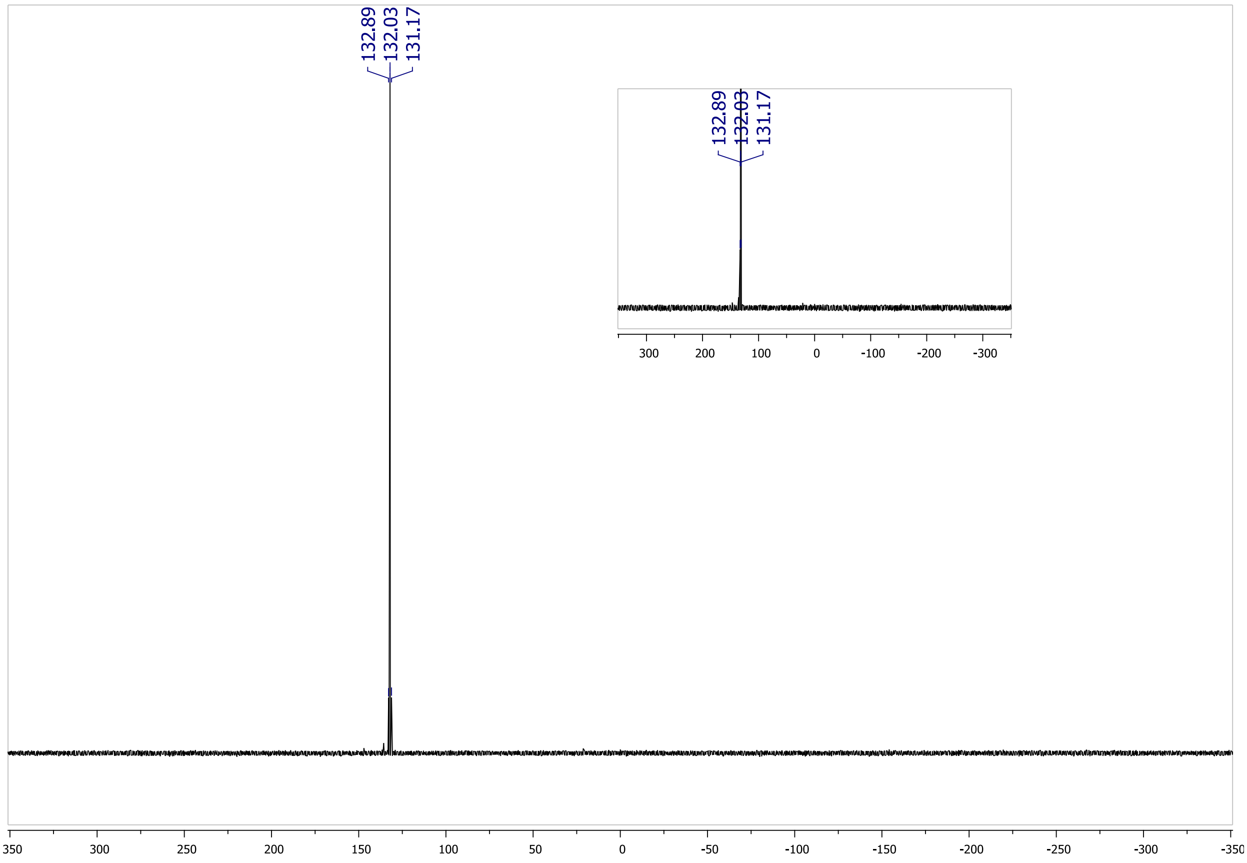

APT - ^{13}C NMR of complex **3b**

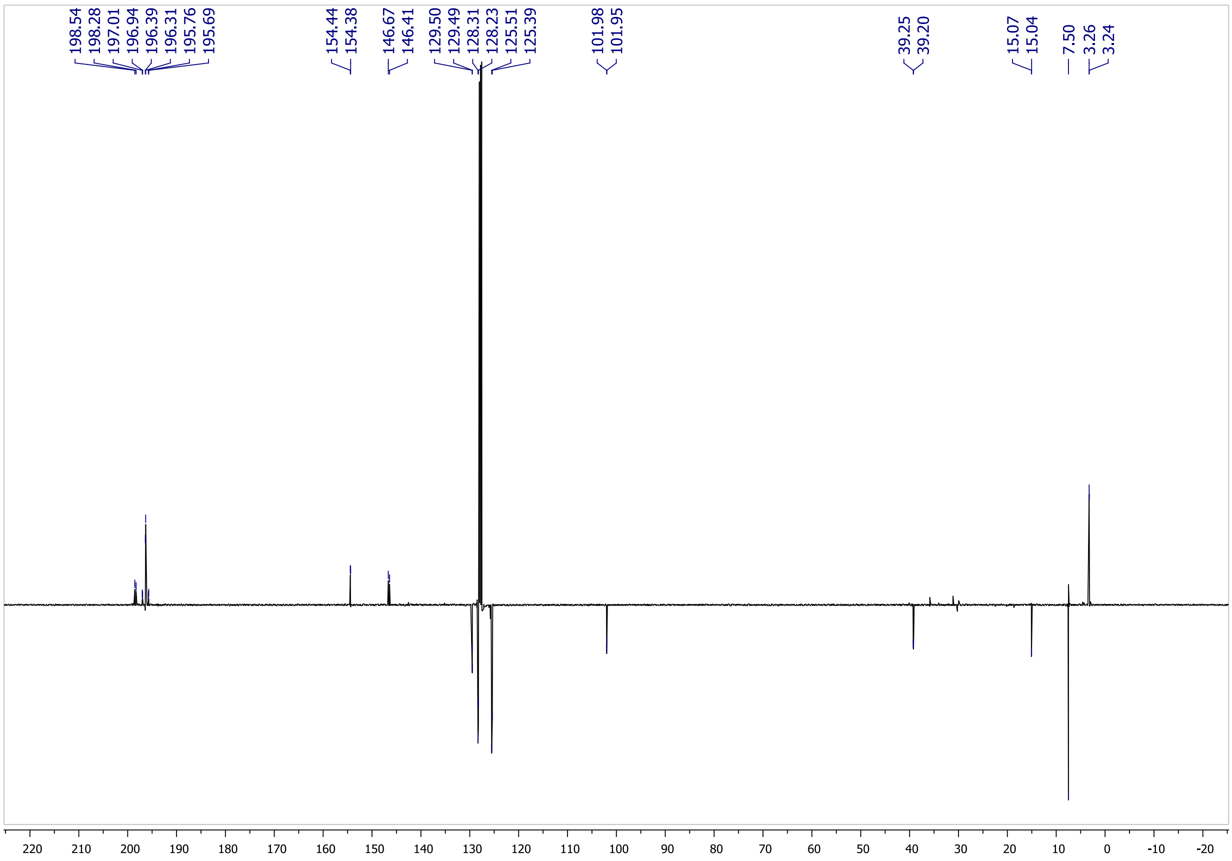

^{1}H NMR of complex **3c**

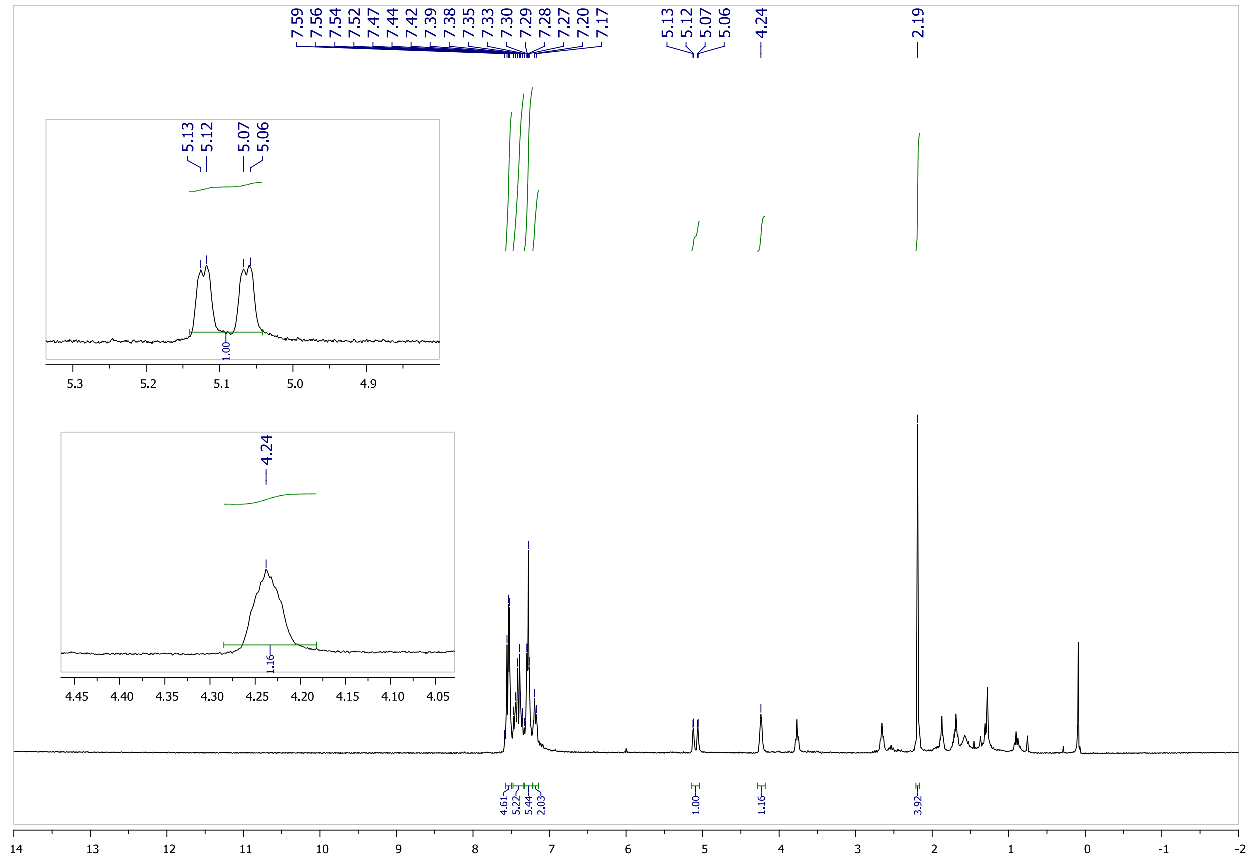

^{31}P NMR of complex **3c**

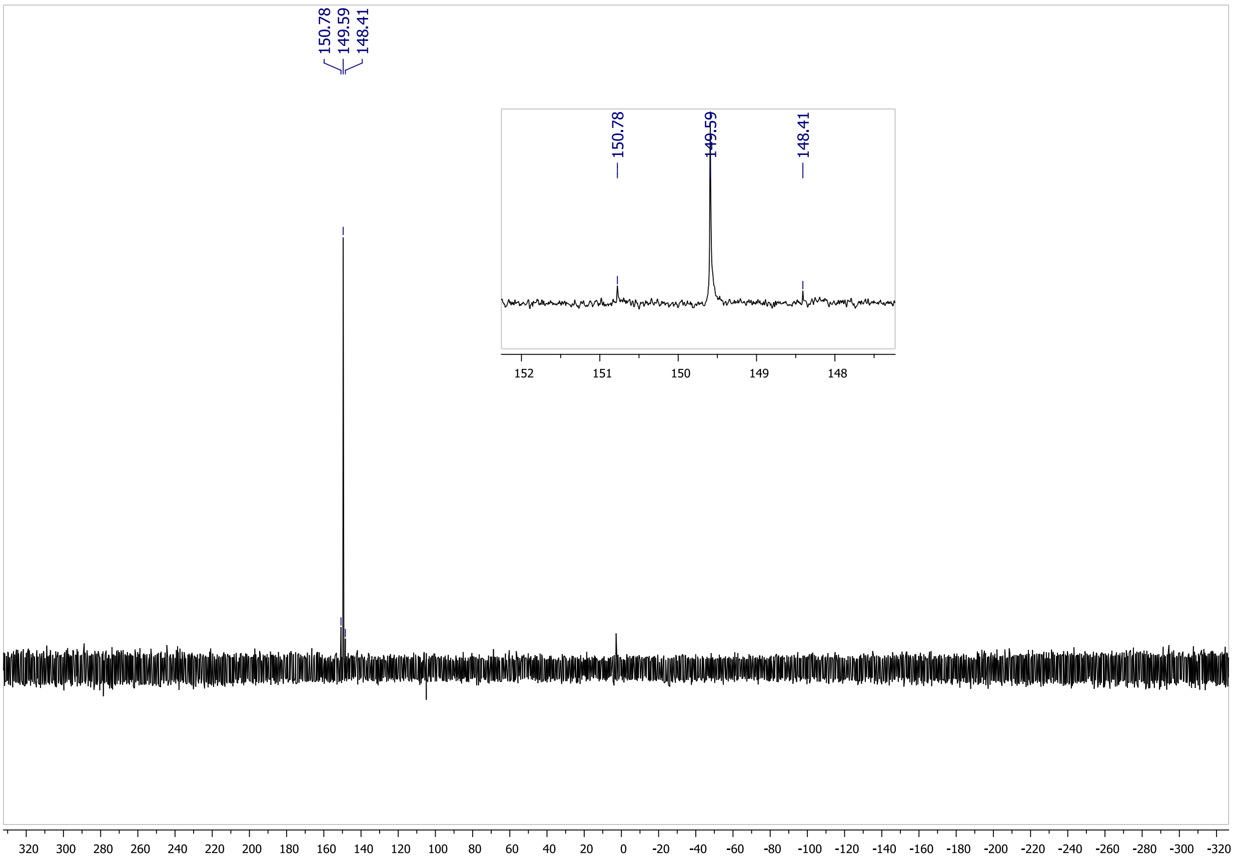

DEPT-135 ^{13}C NMR of complex **3c**

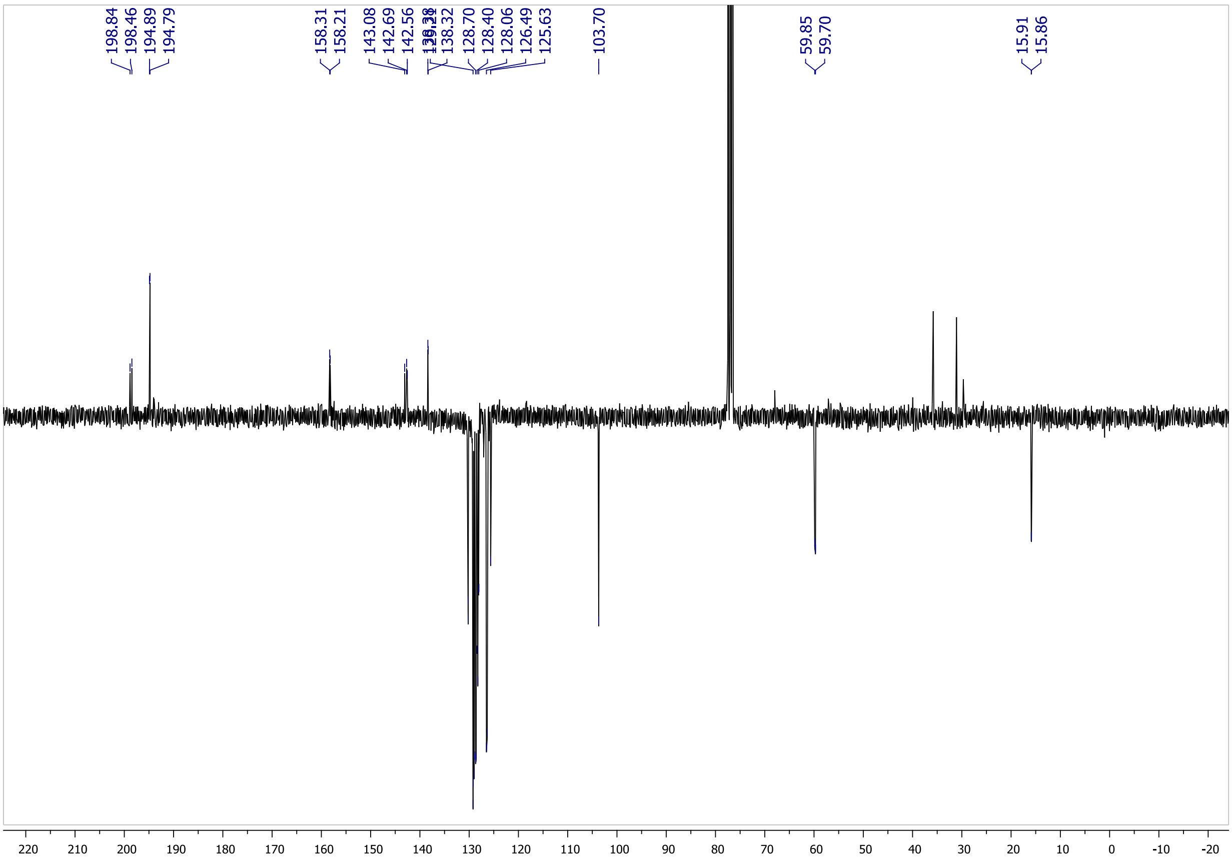

^{1}H NMR of complex **3d**

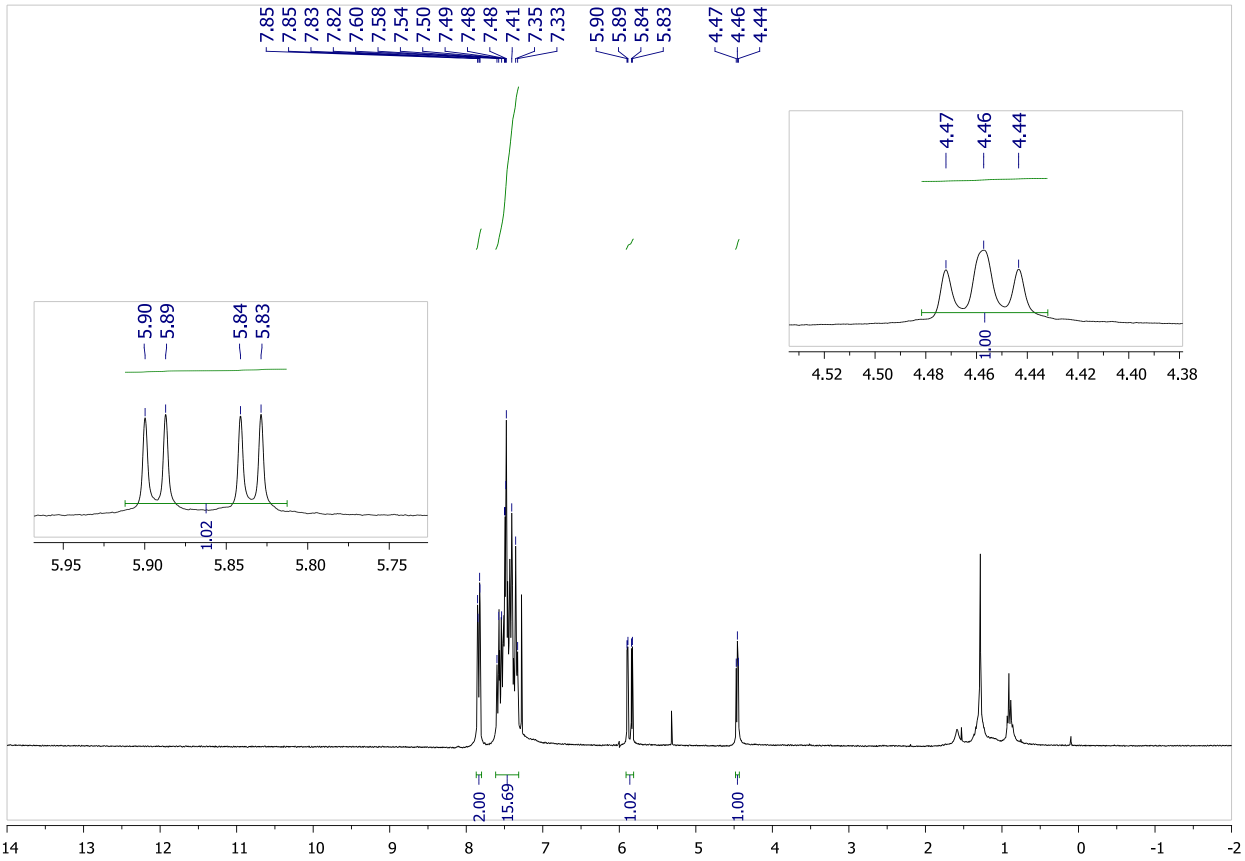

^{31}P NMR of complex **3d**

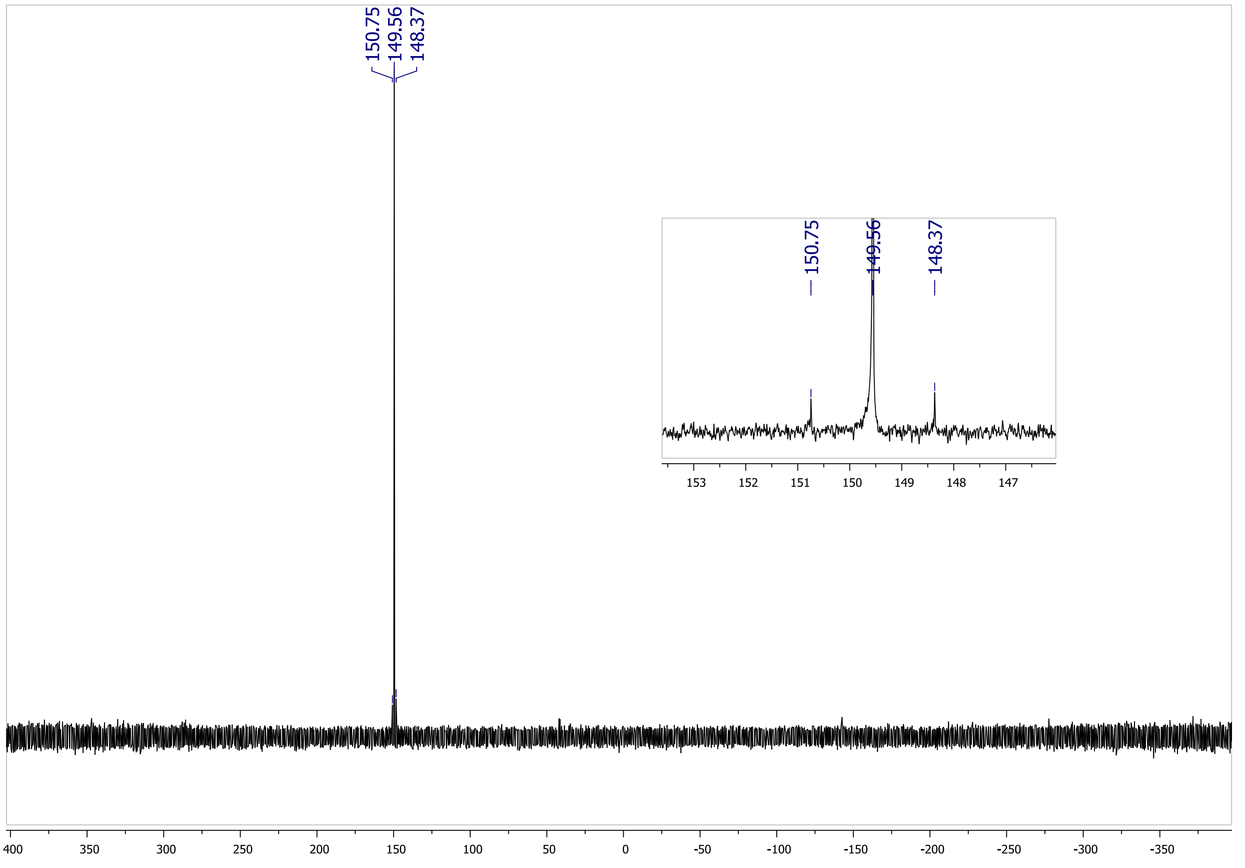

APT - ^{13}C NMR of complex **3d**

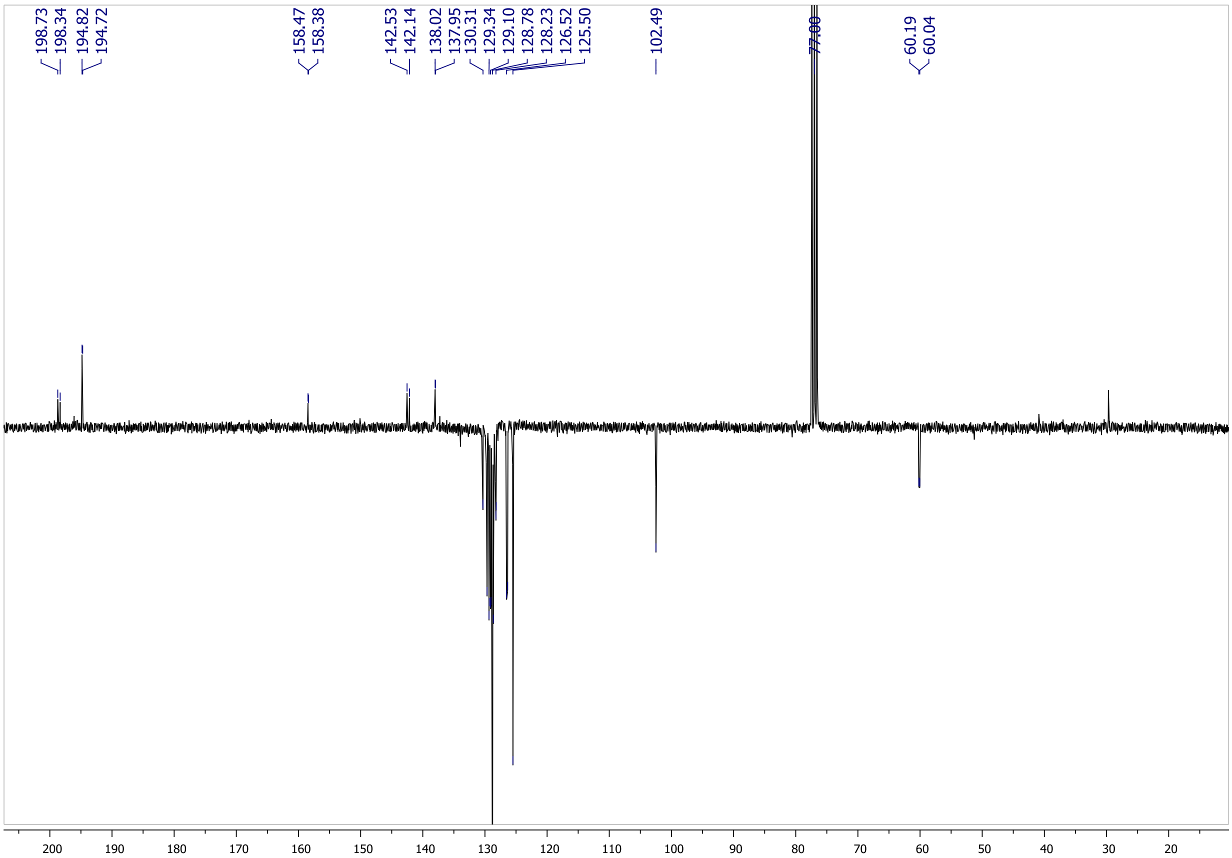

^{1}H NMR of complex **3e**

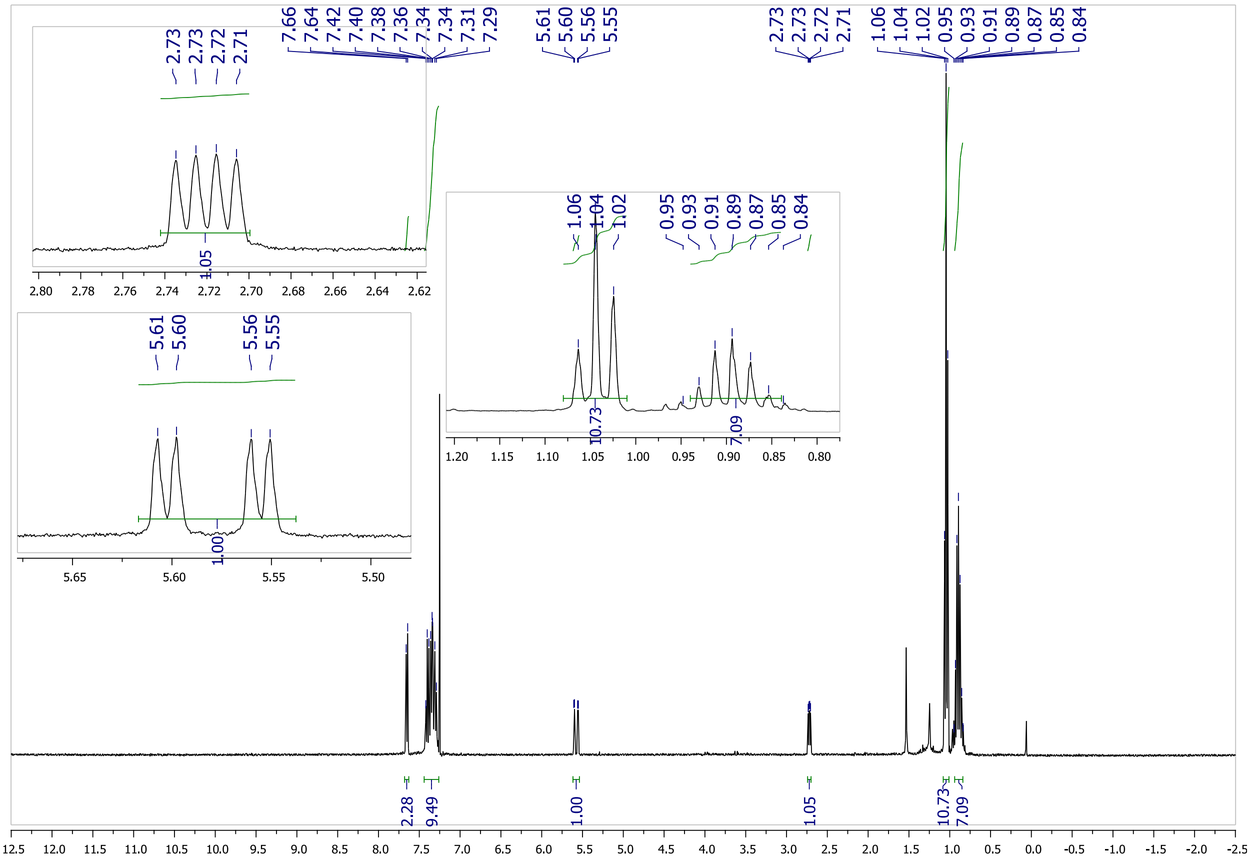

^{31}P NMR of complex **3e**

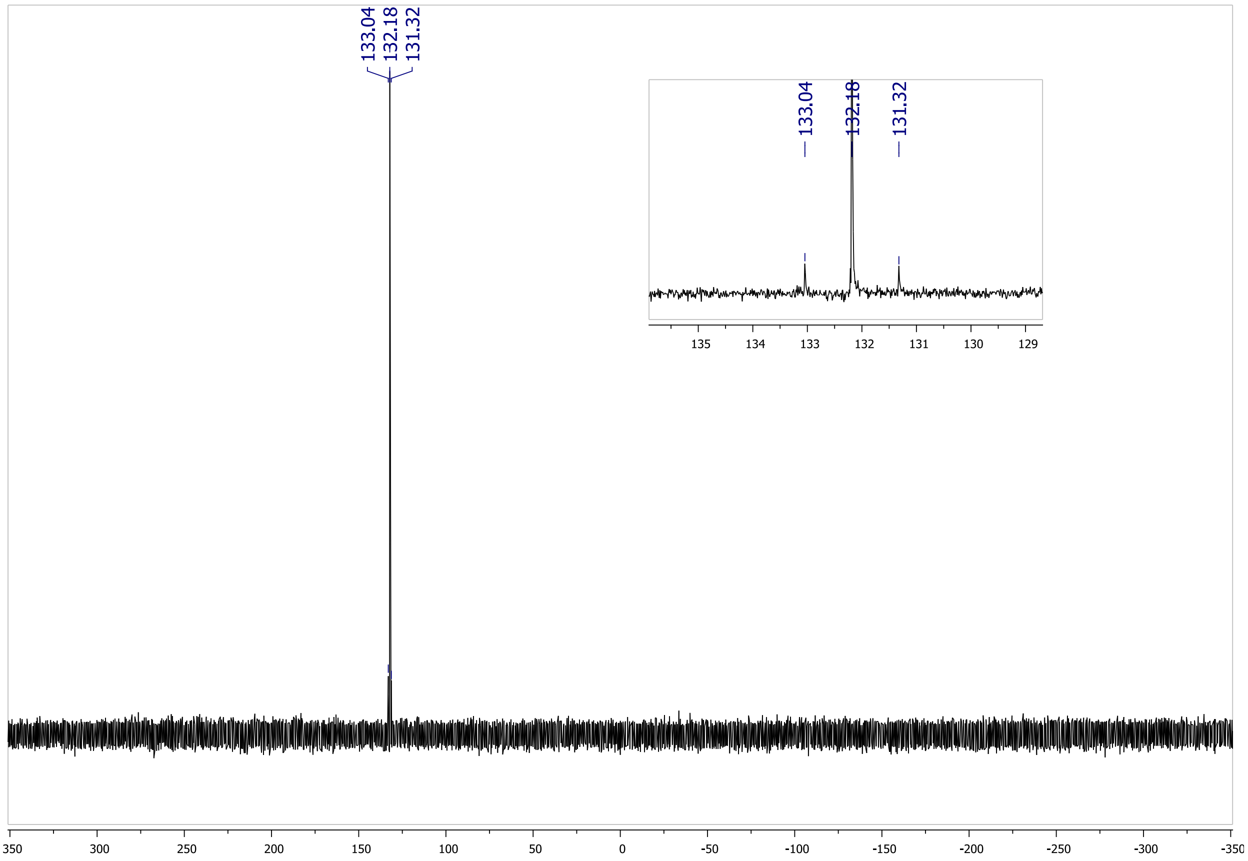

^{13}C NMR of complex **3e**

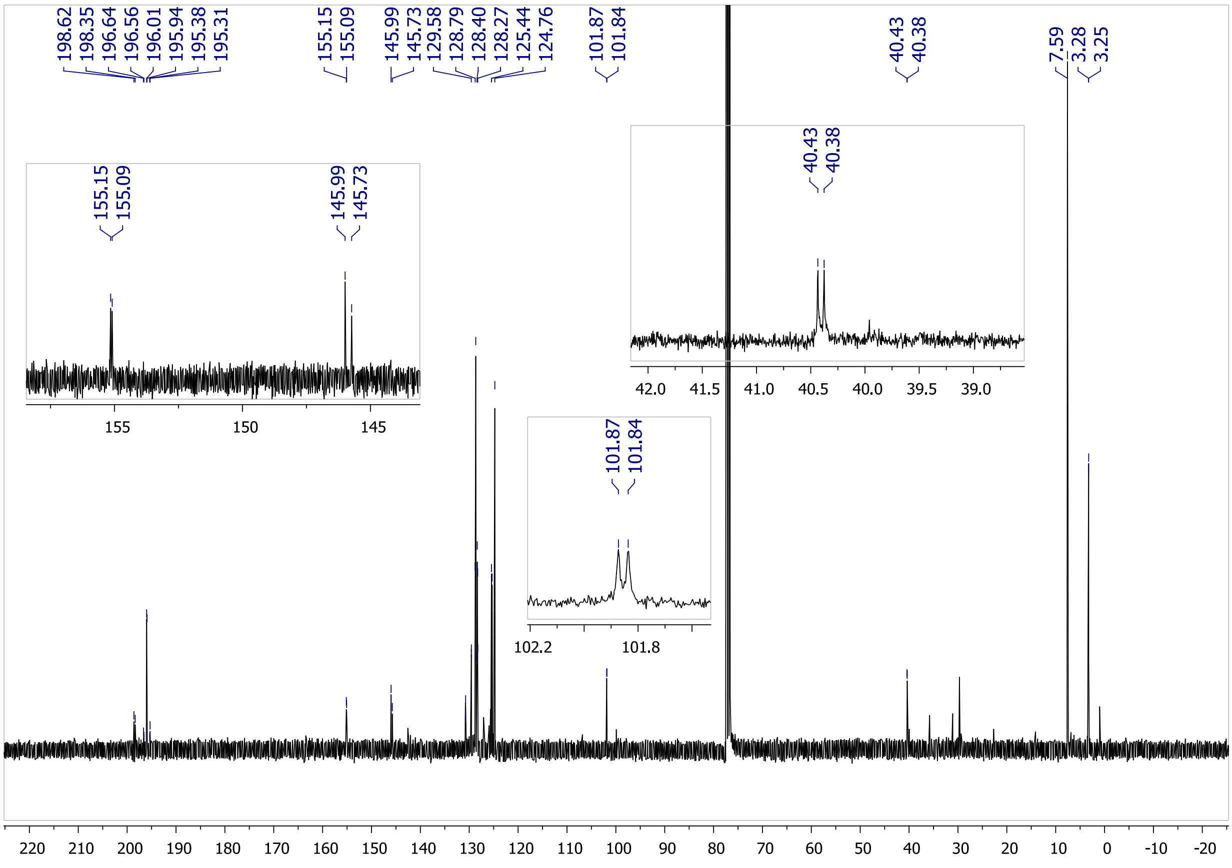

^{1}H NMR of complex **4c**

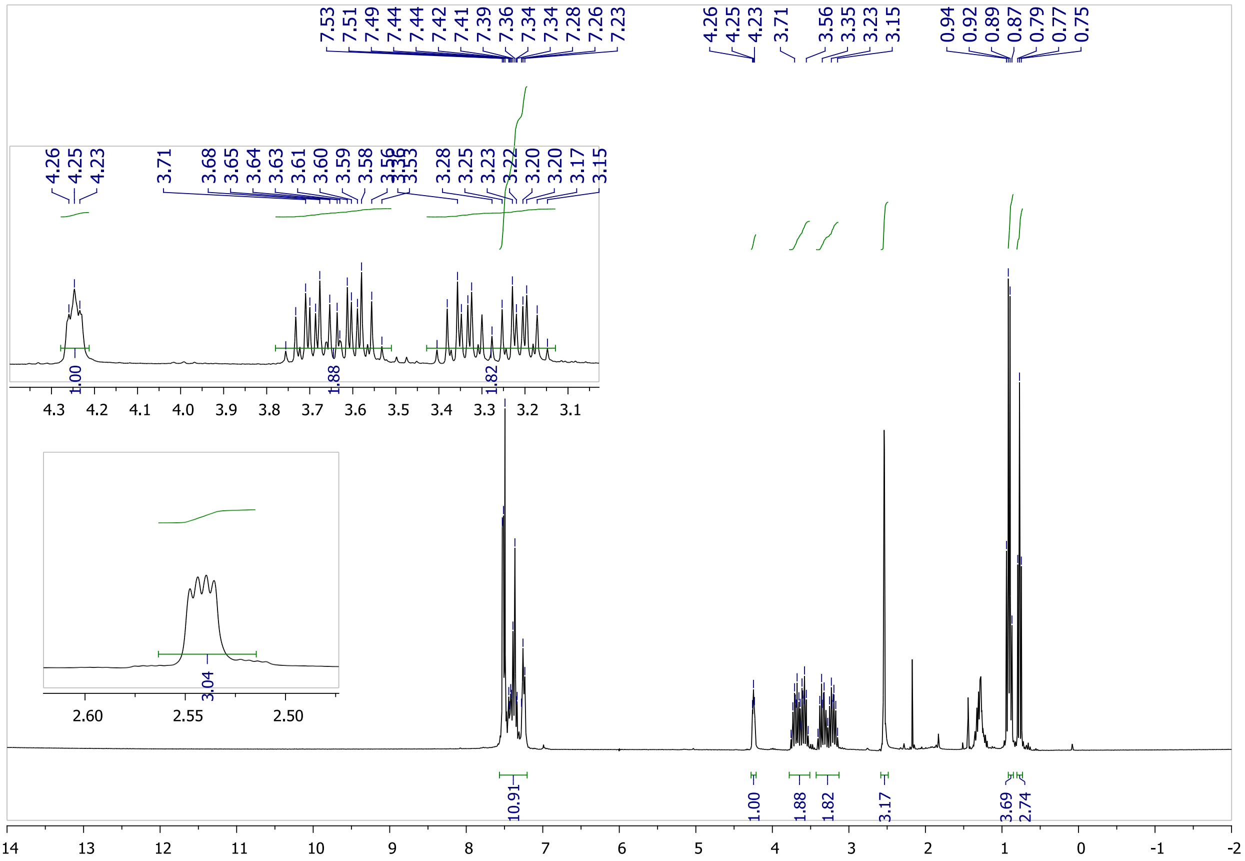

^{31}P NMR of complex **4c**

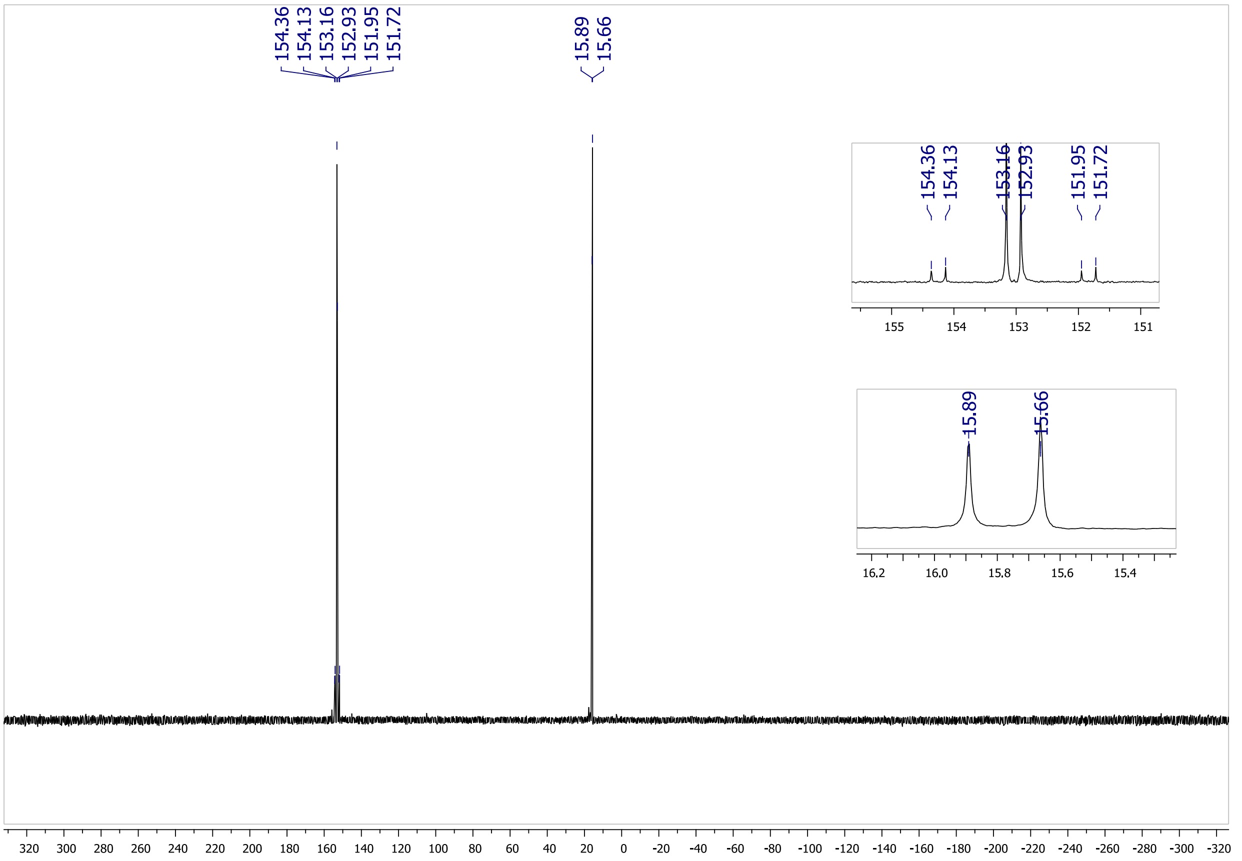

APT - ^{13}C NMR of complex **4c**

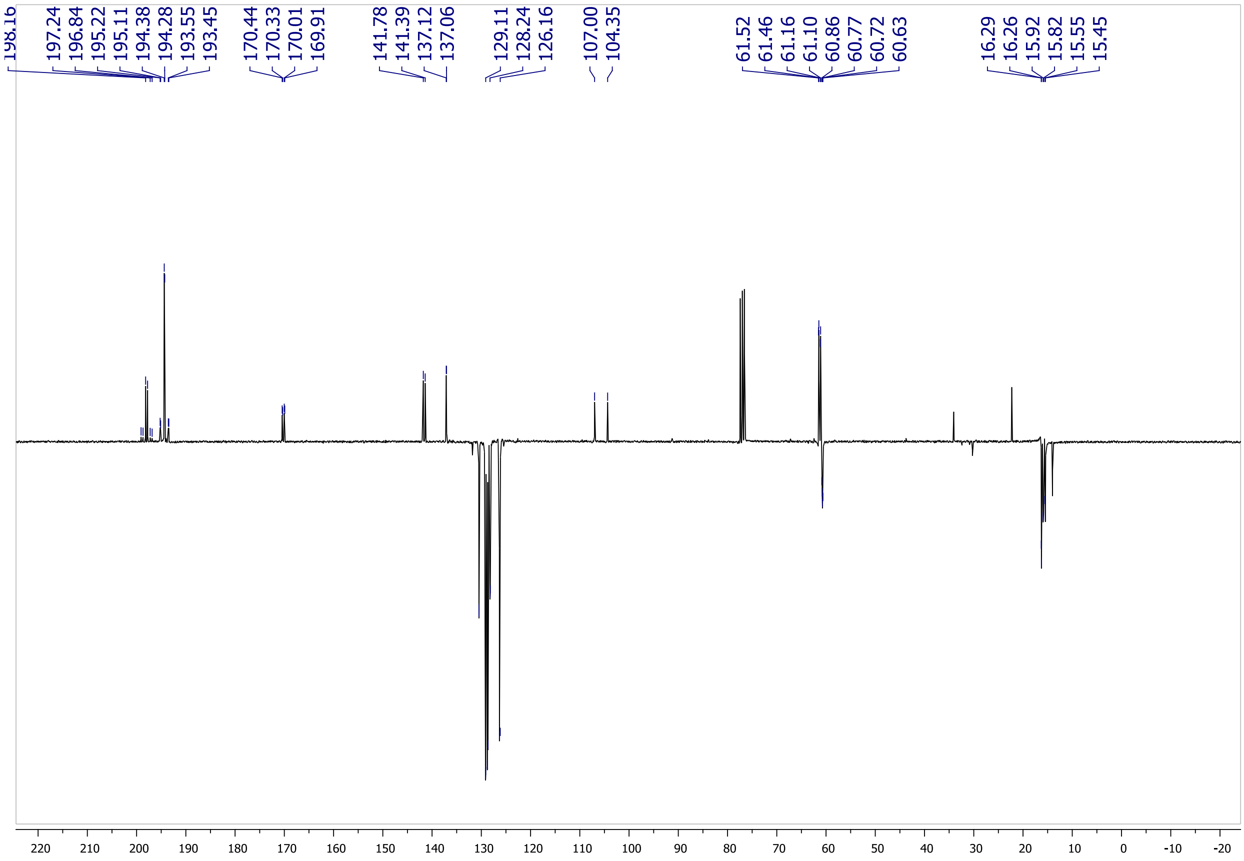

^{1}H NMR of complex **4d**

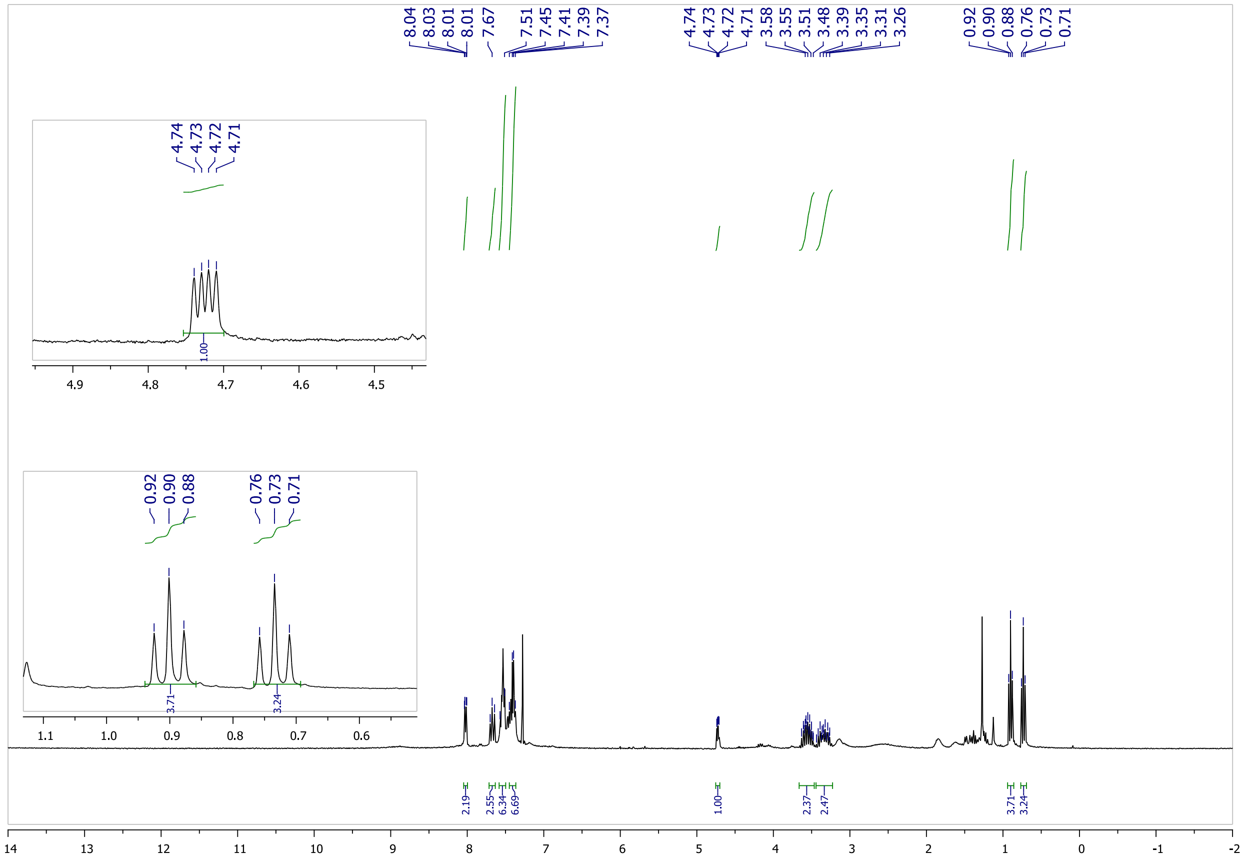

^{31}P NMR of complex **4d**

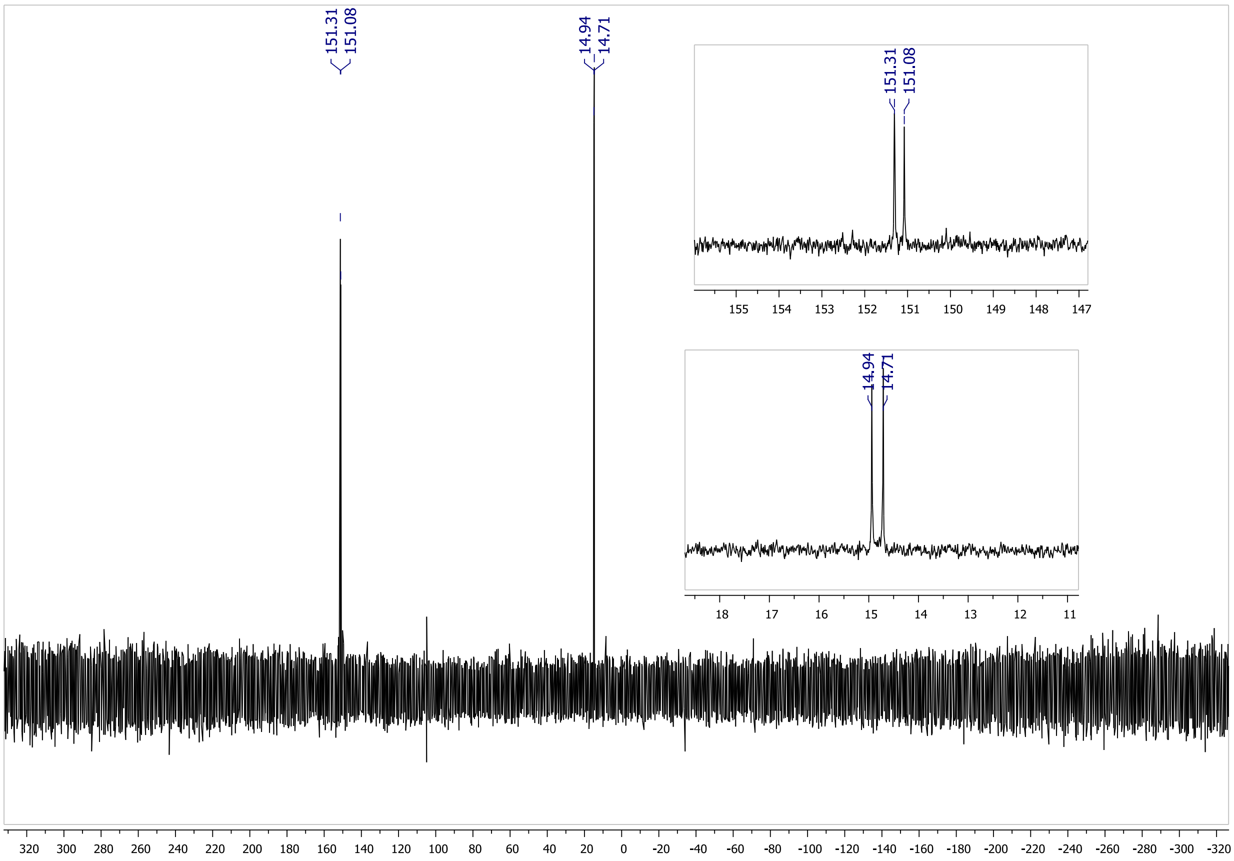

APT - ^{13}C NMR of complex **4d**

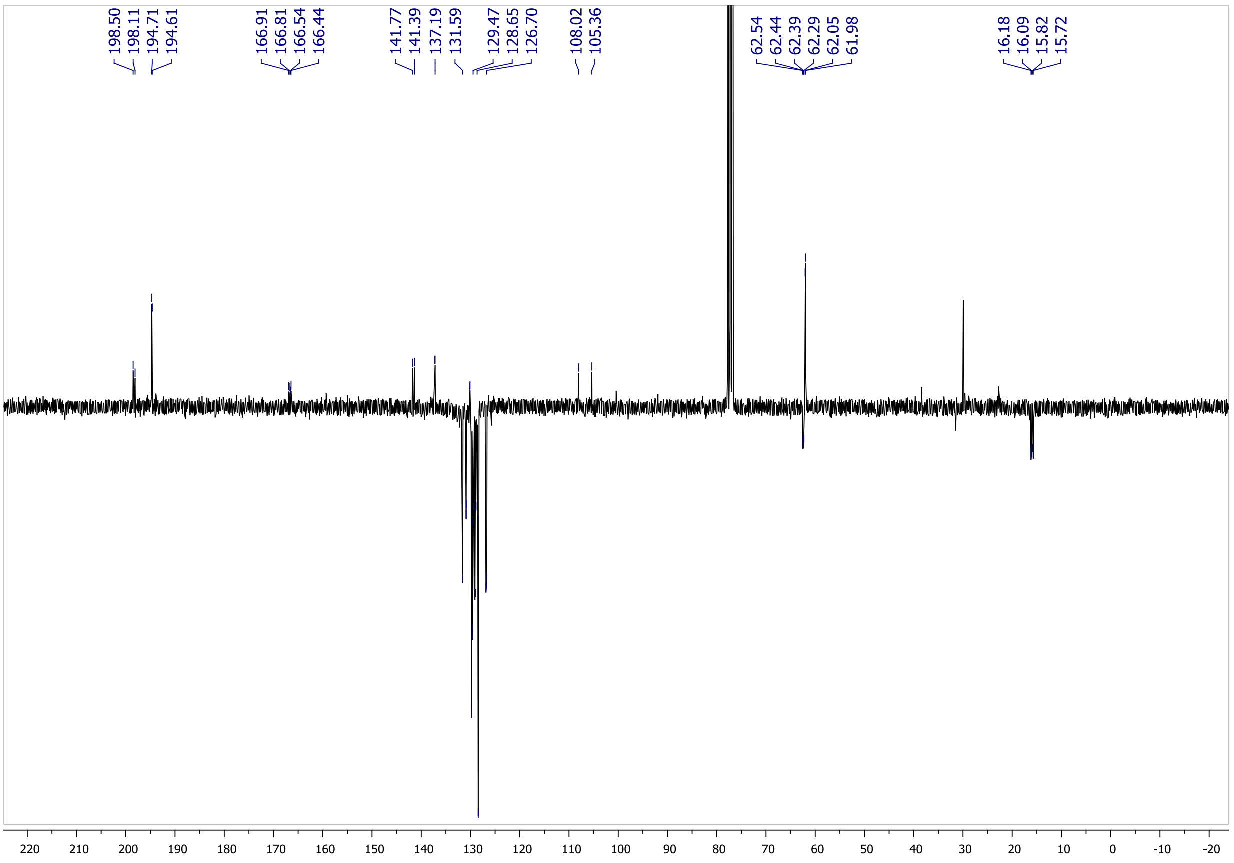

^{1}H NMR of complex **4e**

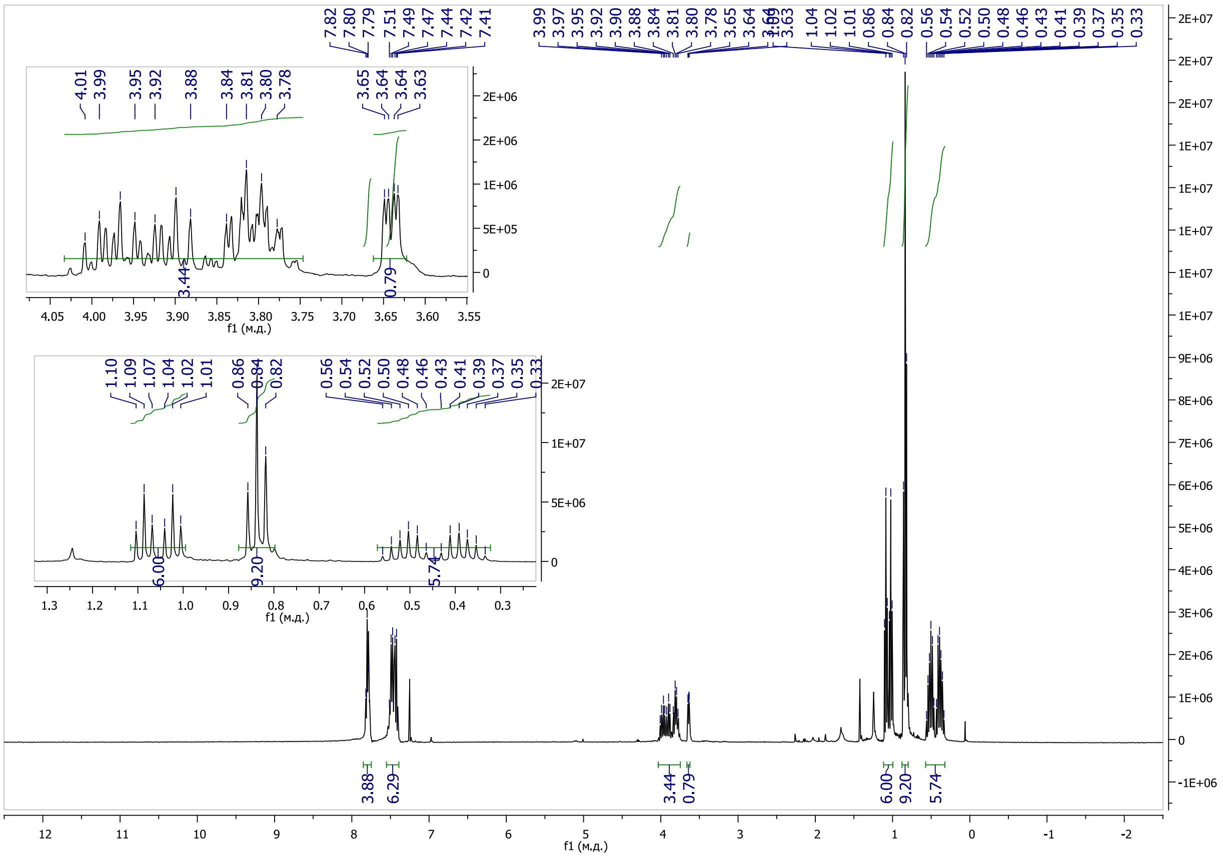

^{31}P NMR of complex **4e**

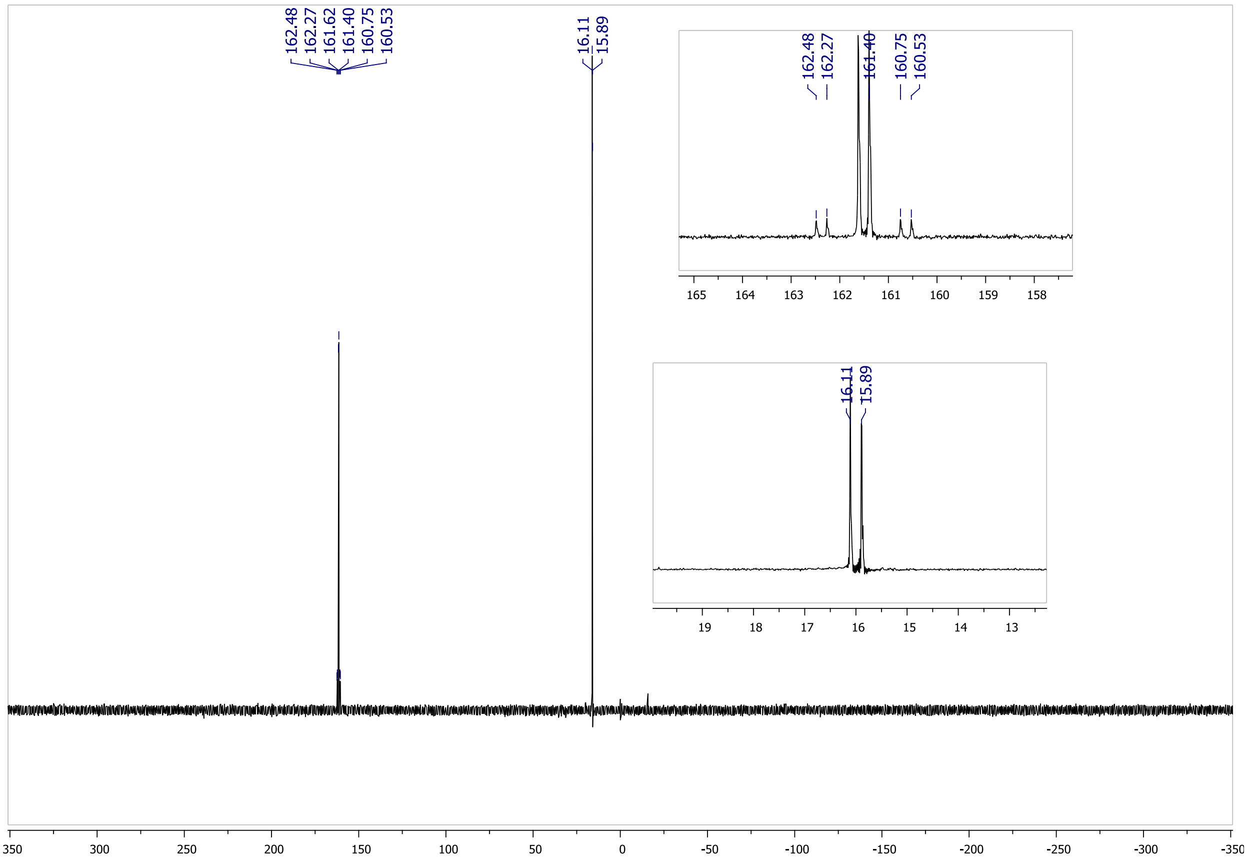

^{13}C NMR of complex **4e**

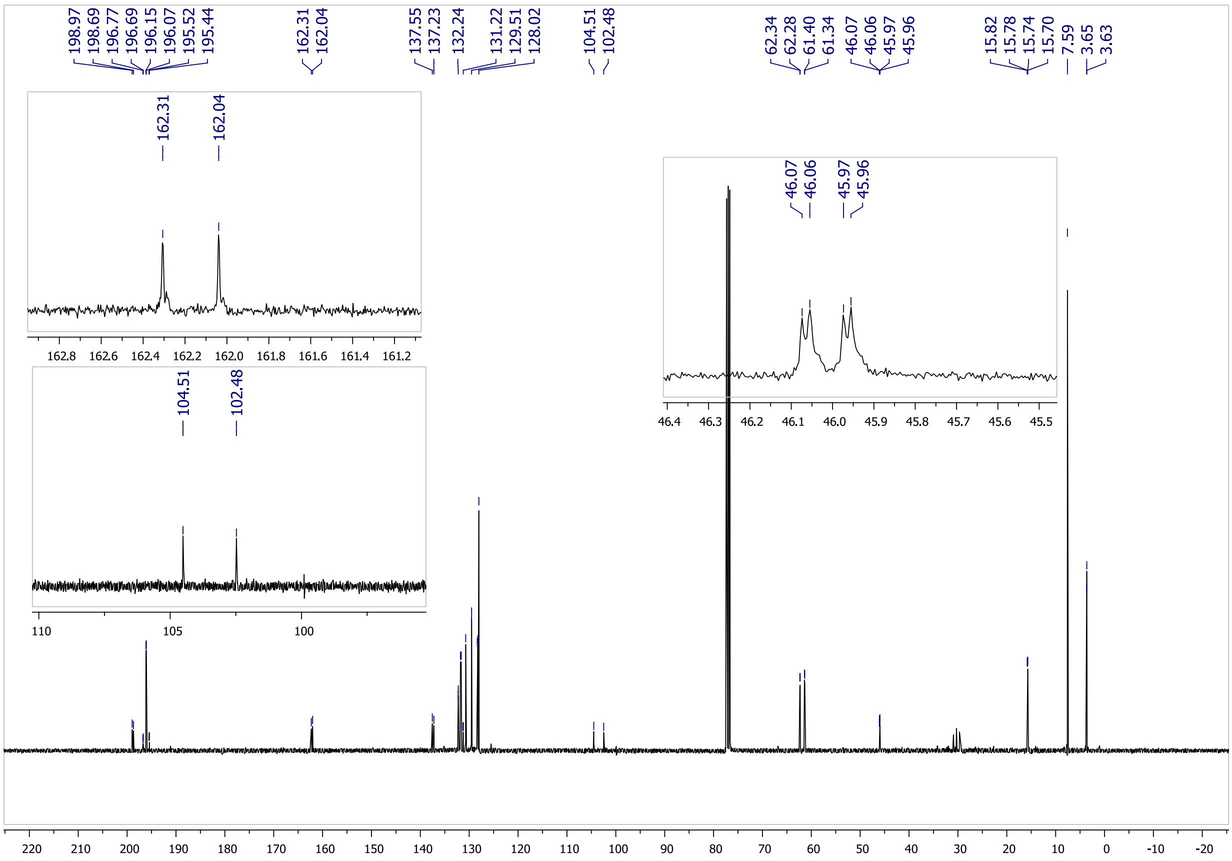

^{1}H NMR of complex **6f**

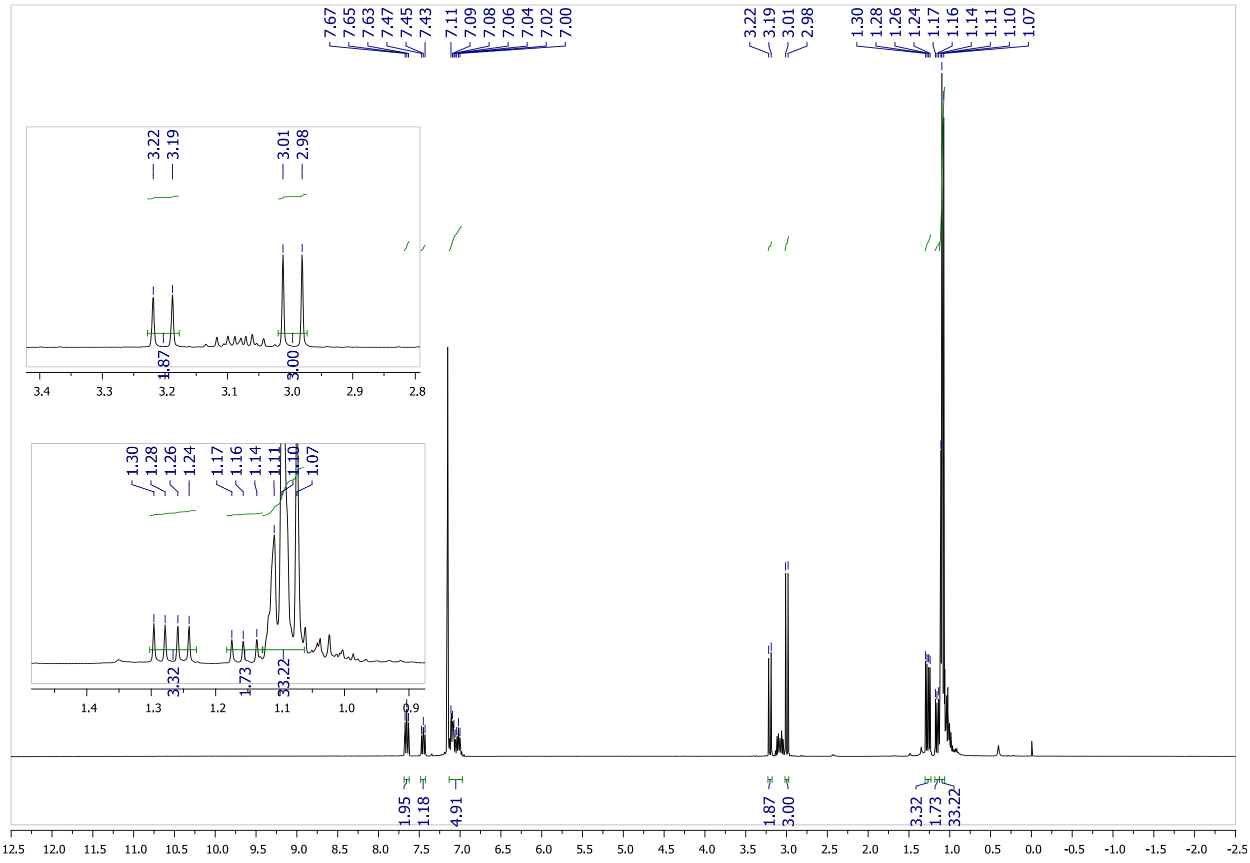

^{31}P NMR of complex **6f**

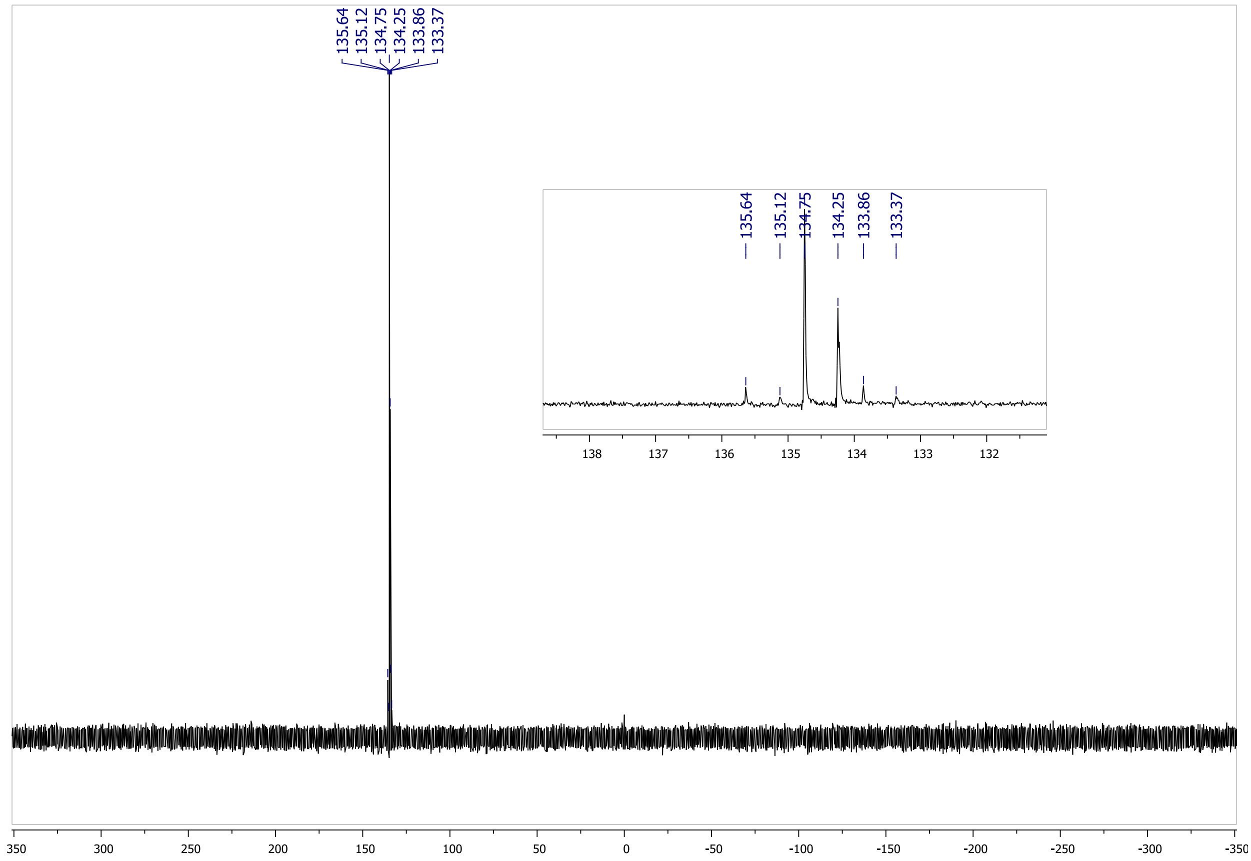

^{13}C NMR of complex **6f**

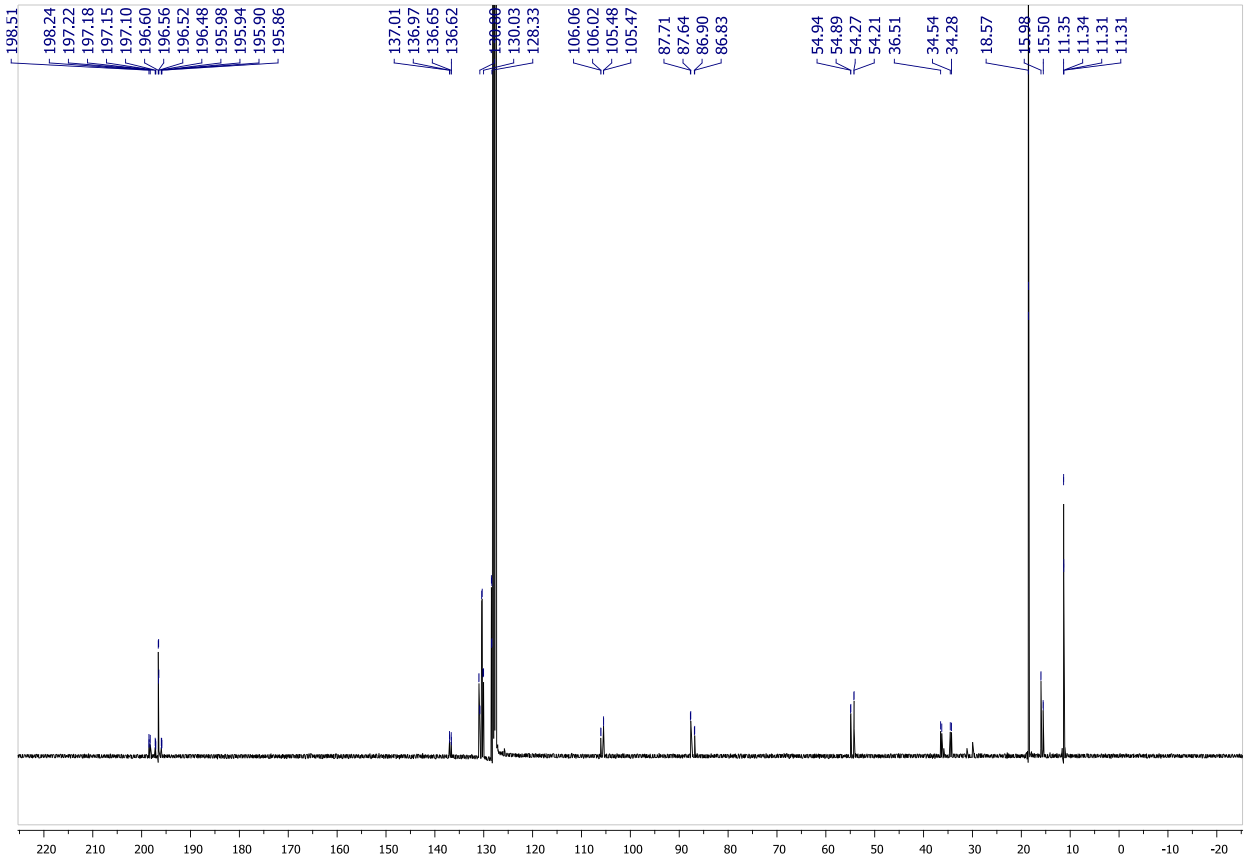

^{1}H NMR of complex **13**

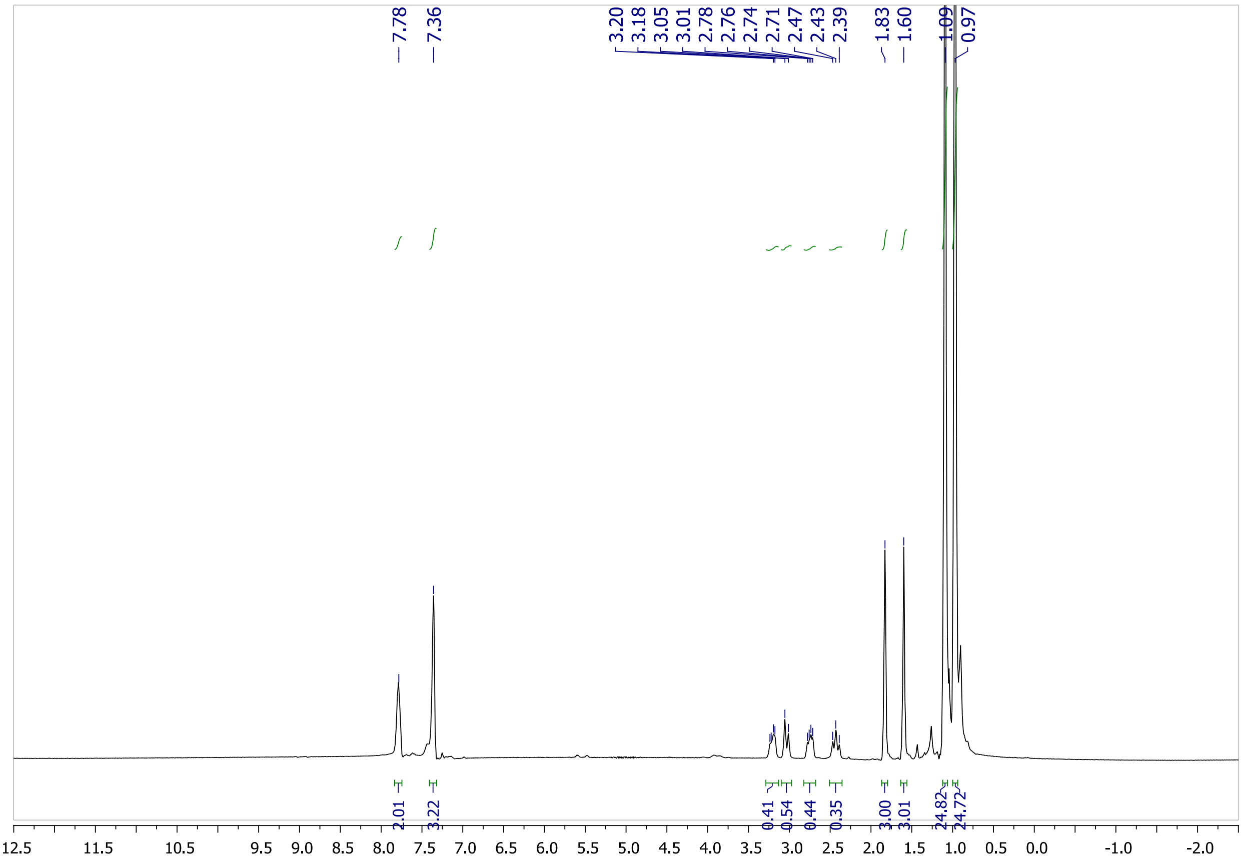

^{31}P NMR of complex **13**

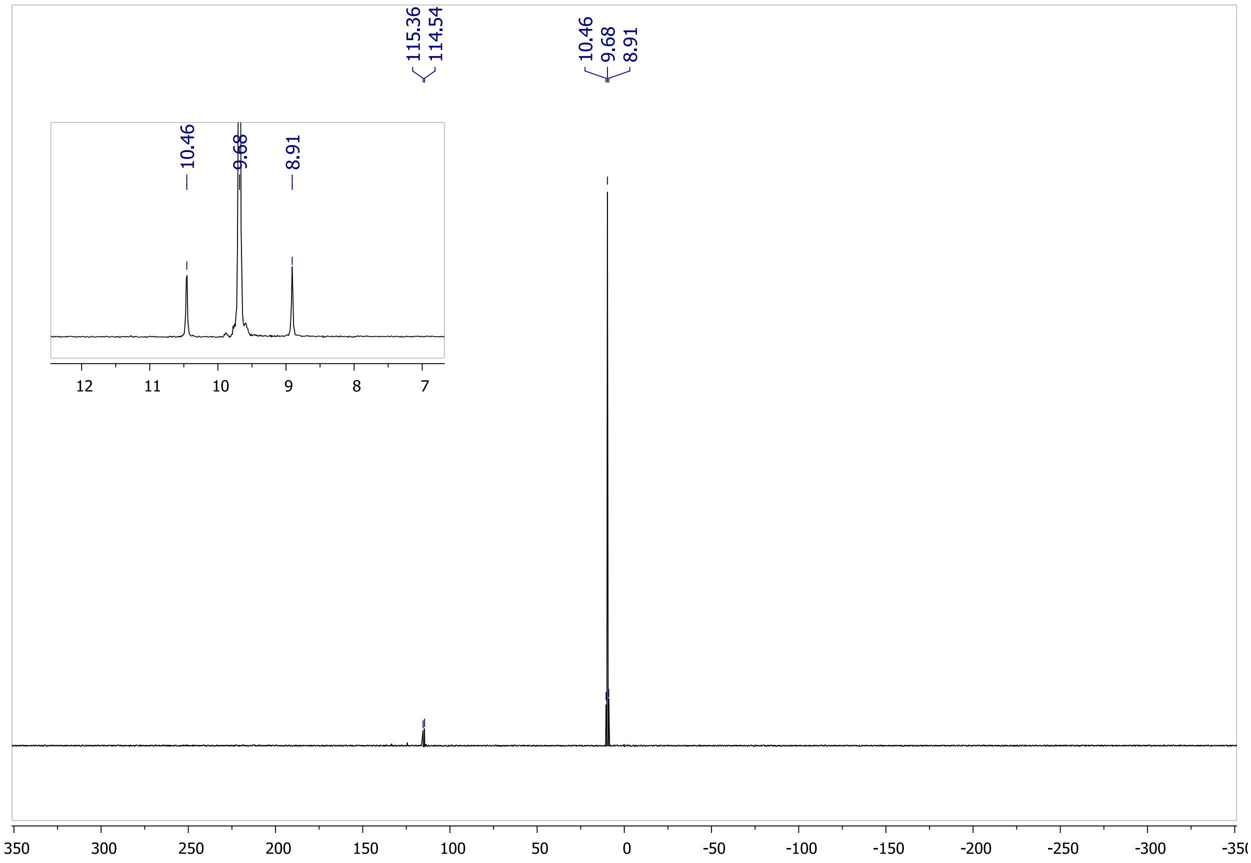

^{13}C NMR of complex **13**

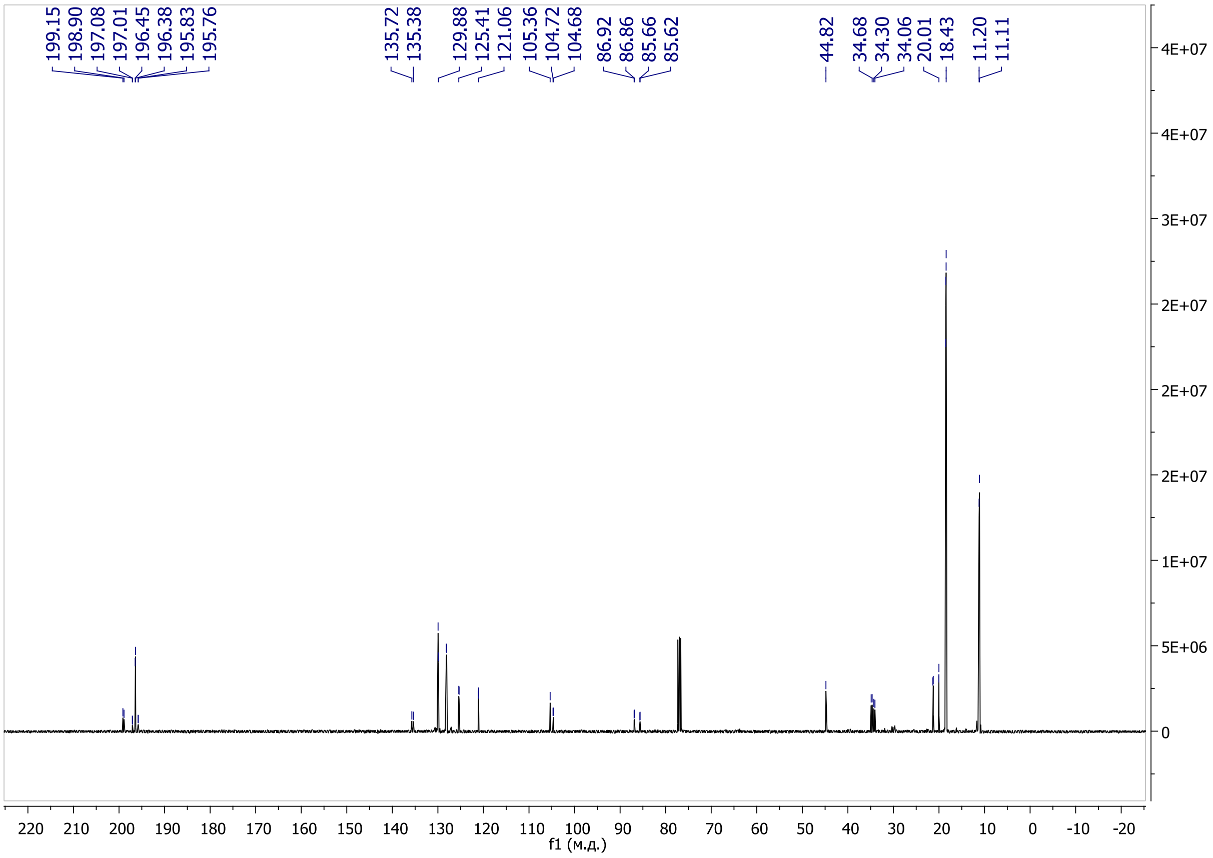

^{1}H NMR of complex **17**

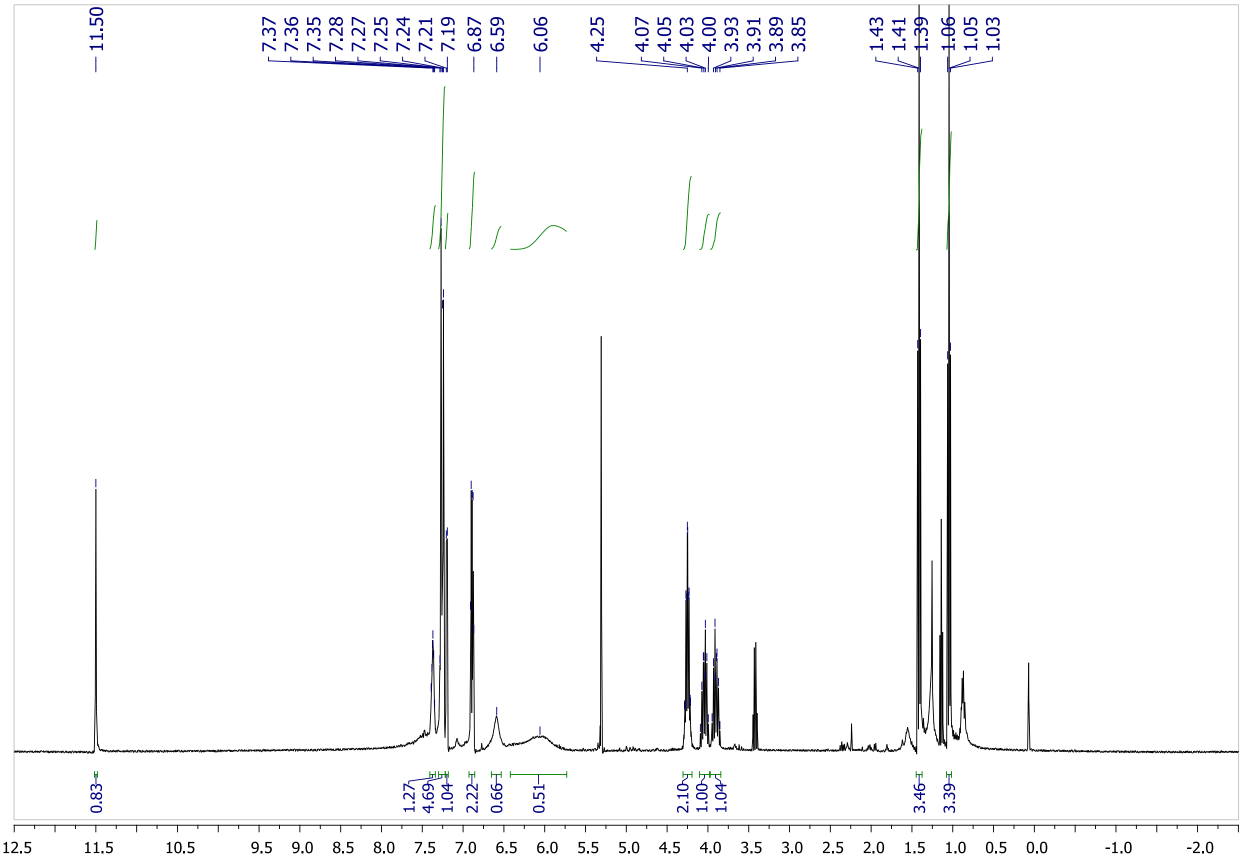

^{31}P NMR of complex **17**

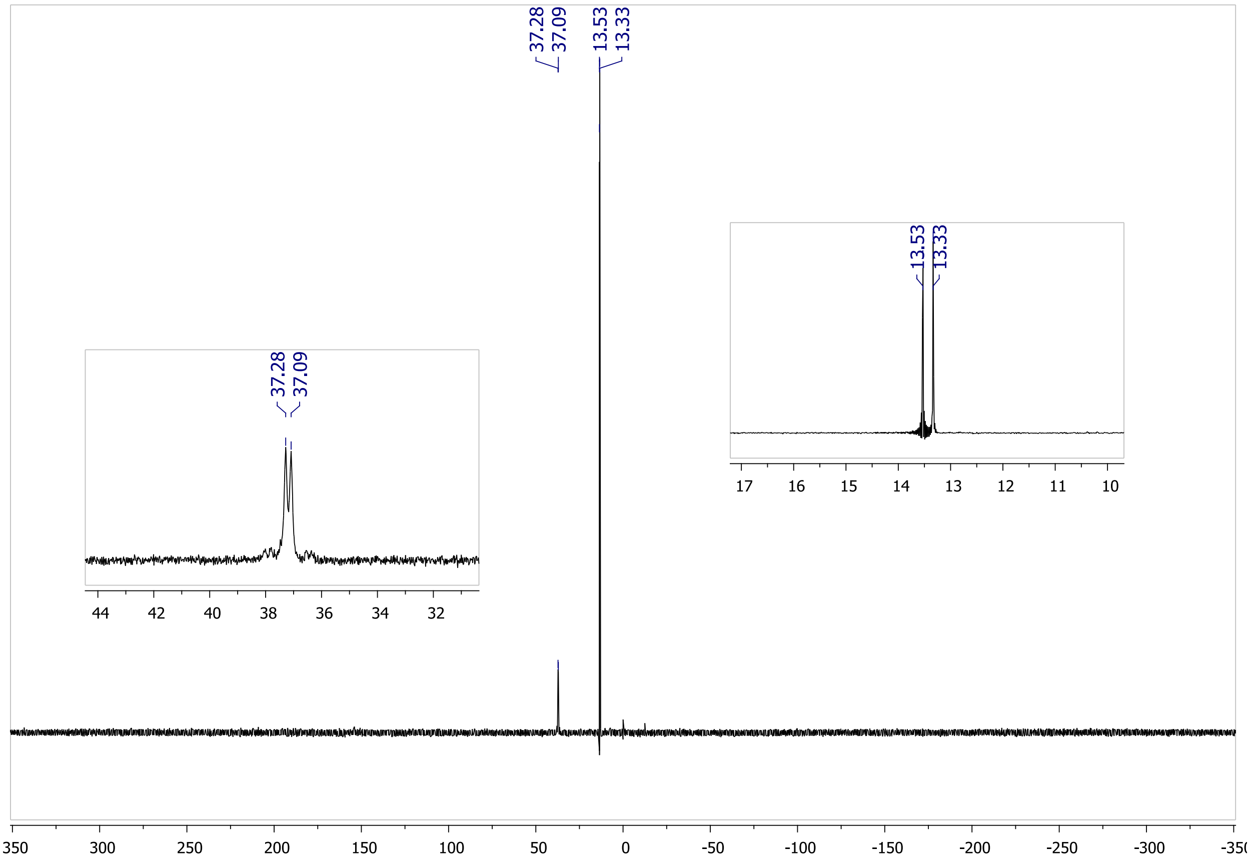

APT - ^{13}C NMR of complex **17**

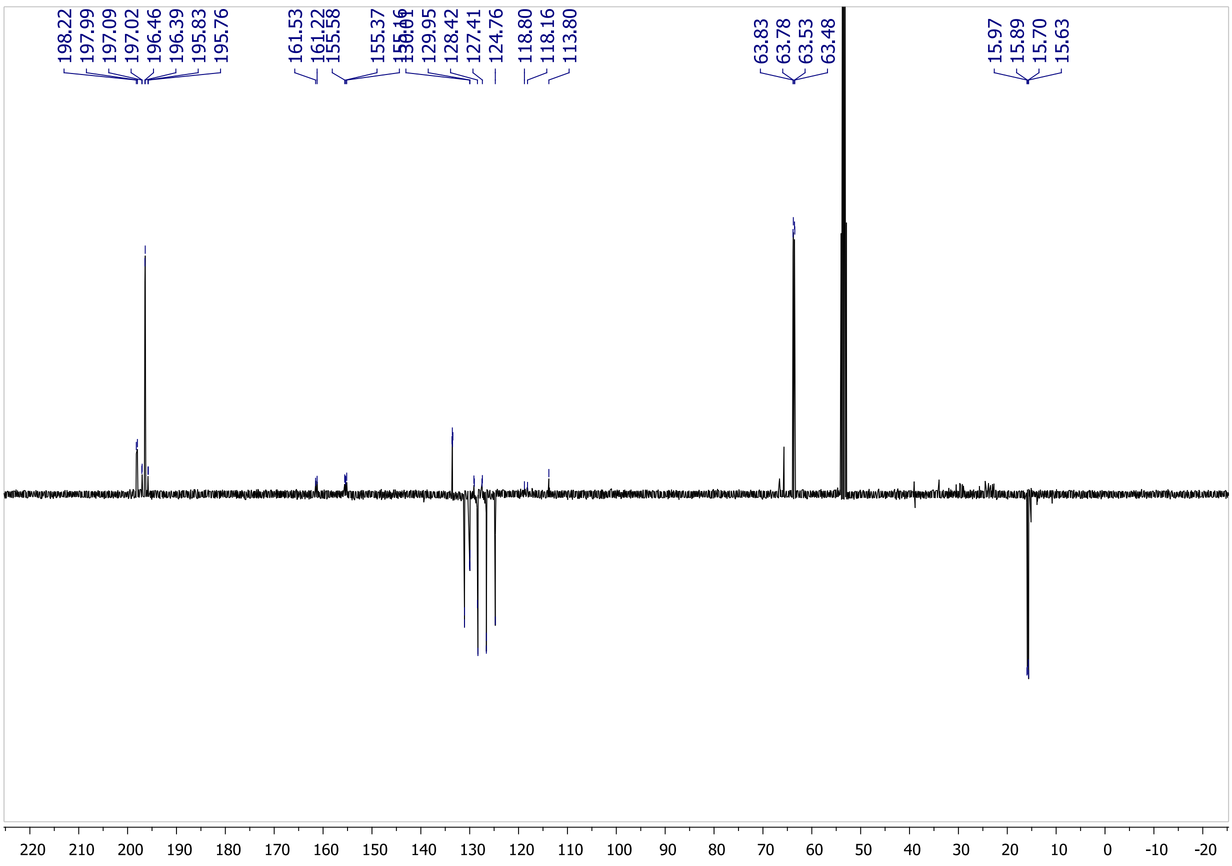

^{1}H NMR of complex **20**

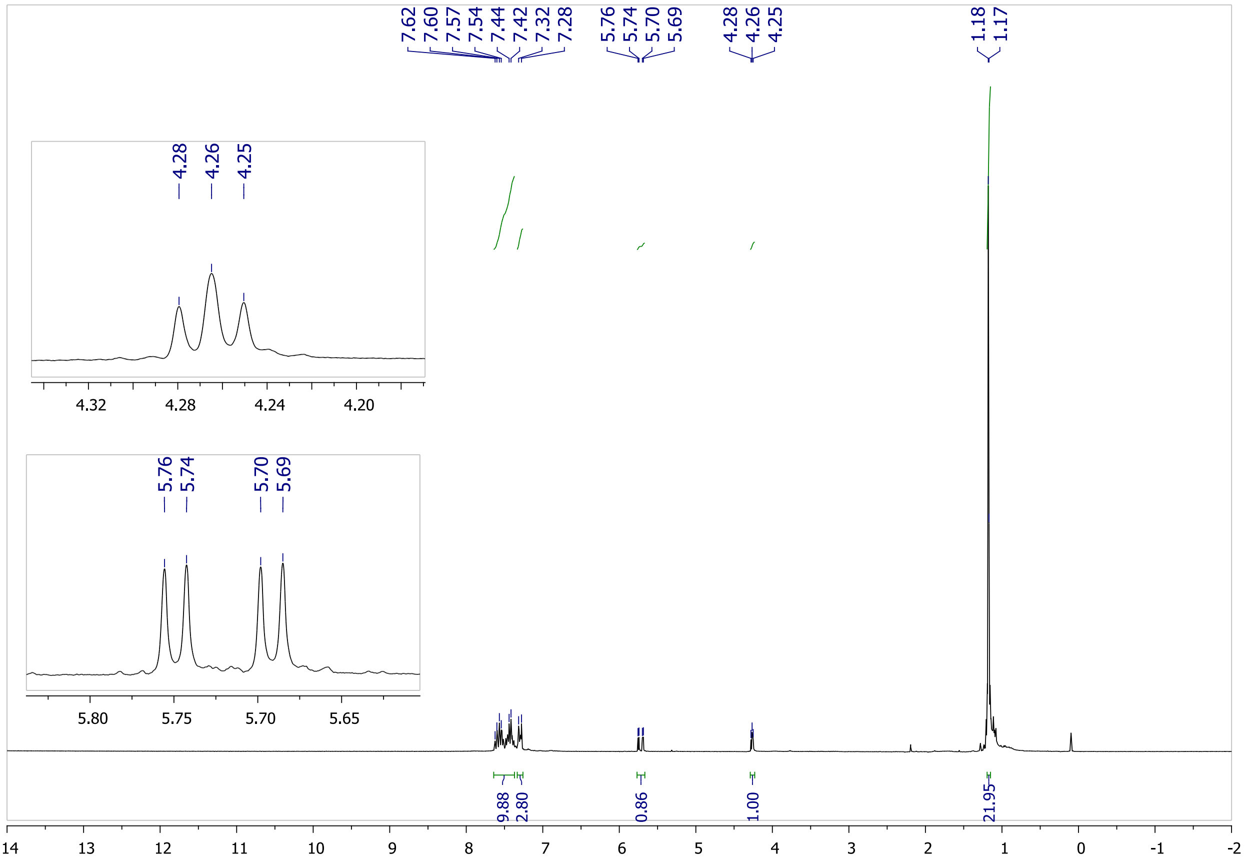

^{31}P NMR of complex **20**

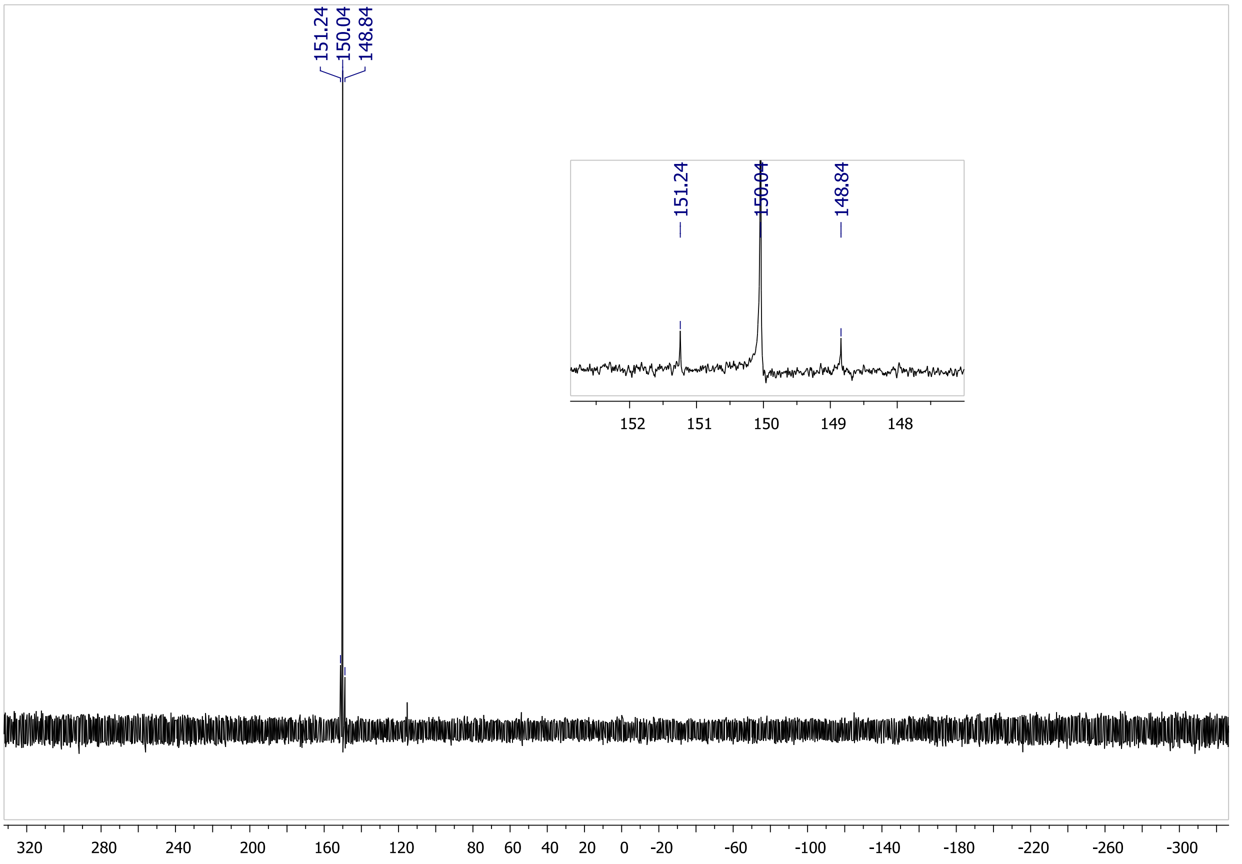

APT - ^{13}C NMR of complex **20**

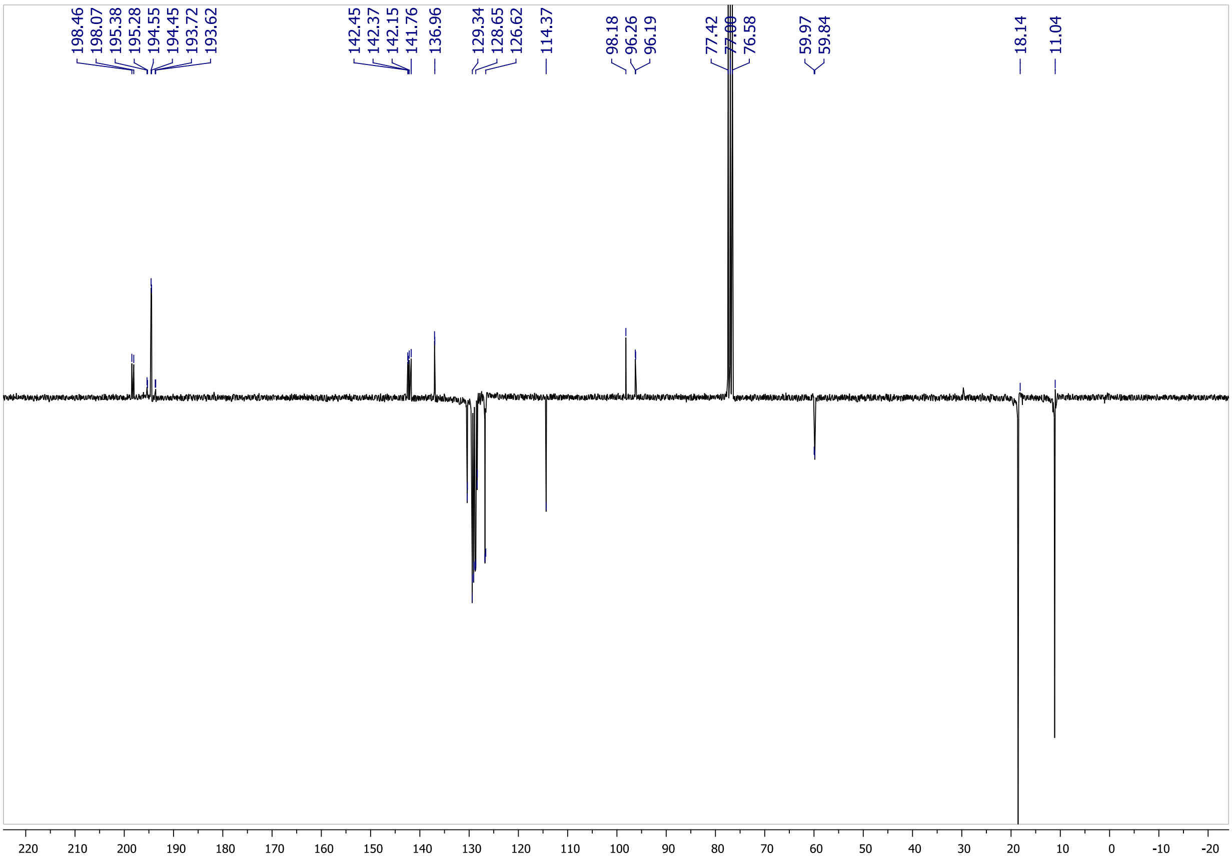

^{1}H NMR of complex **21**

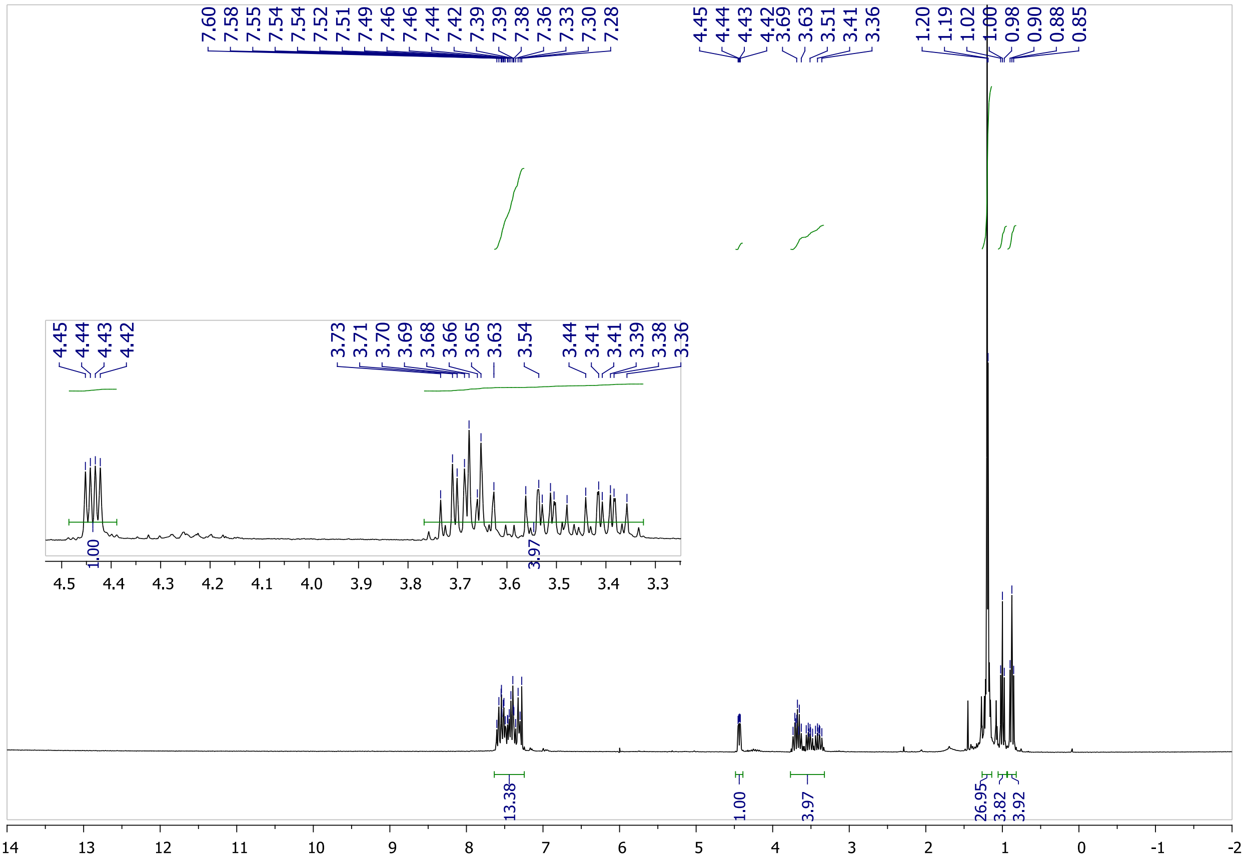

^{31}P NMR of complex **21**

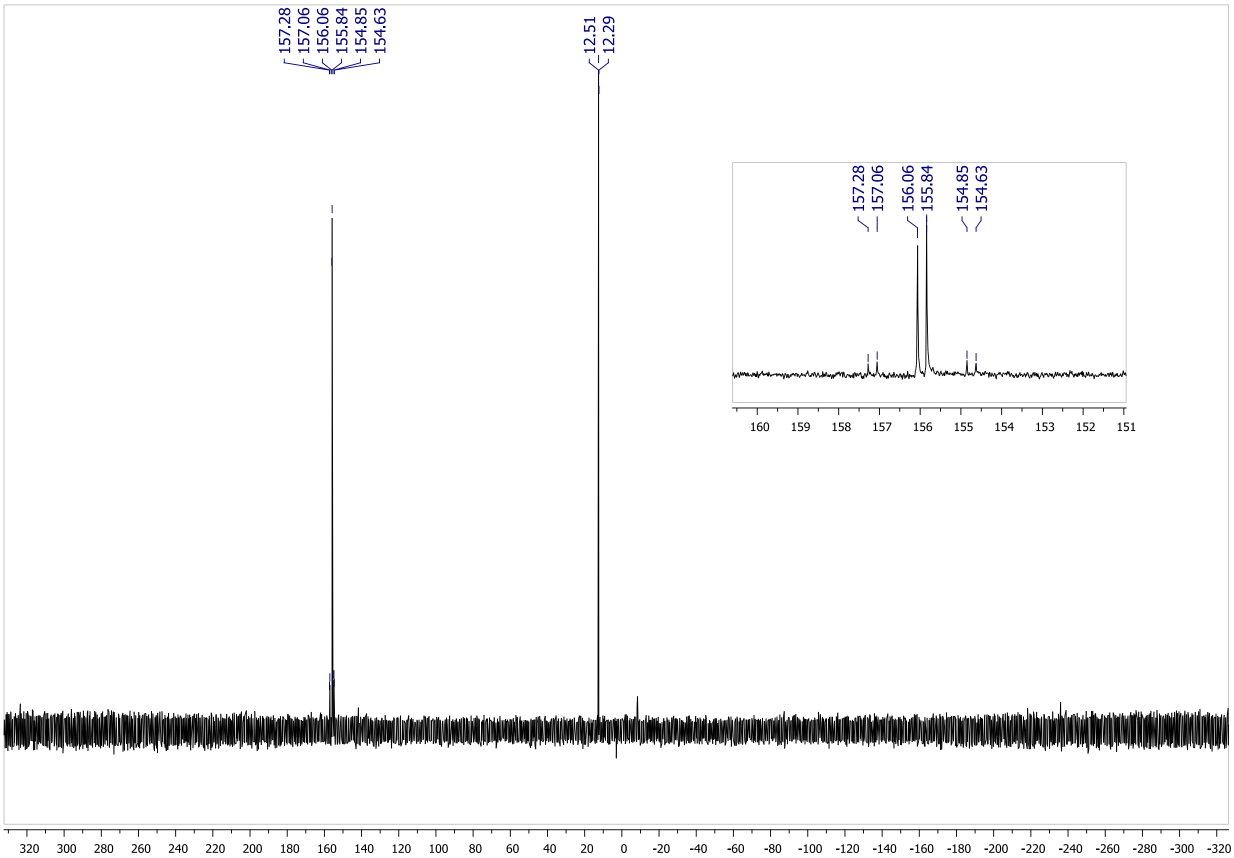

^{13}C NMR of complex **21**

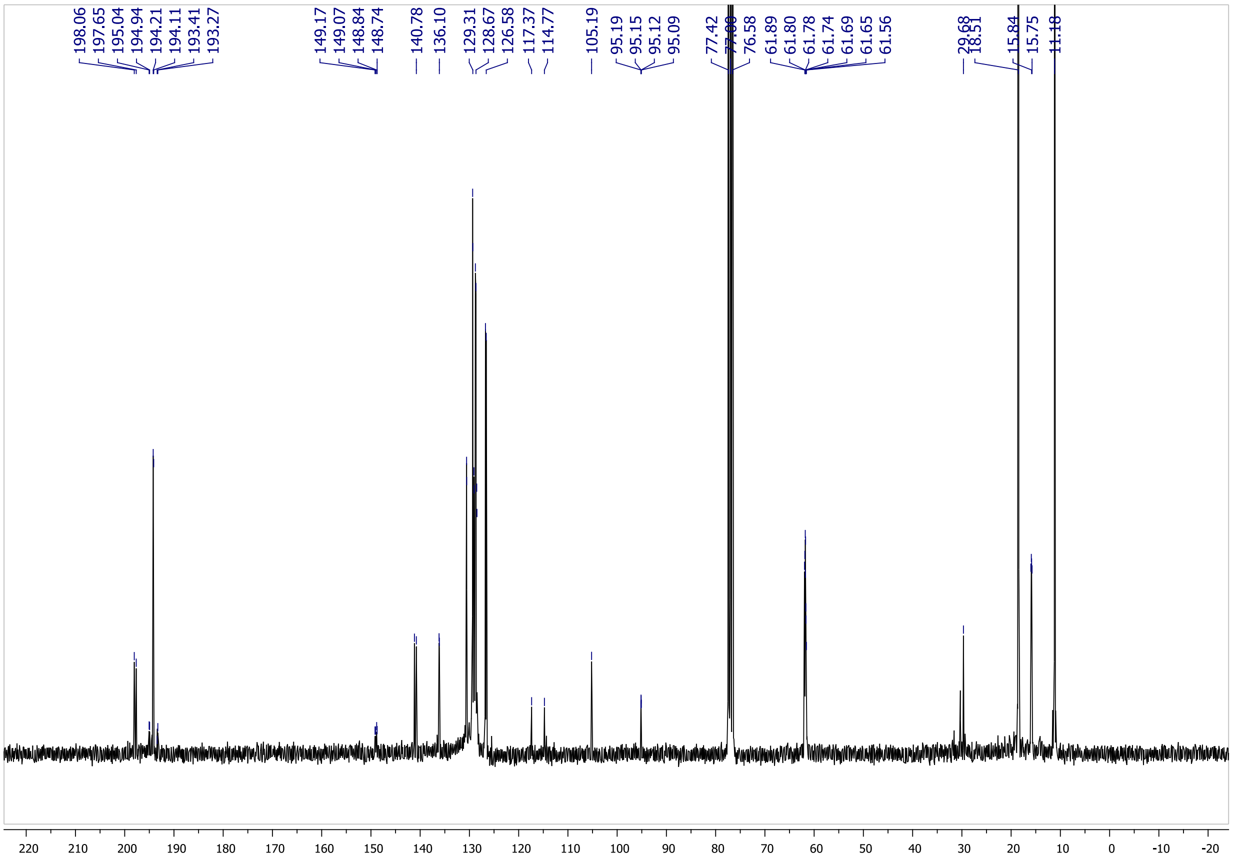

^{1}H NMR of complex **22**

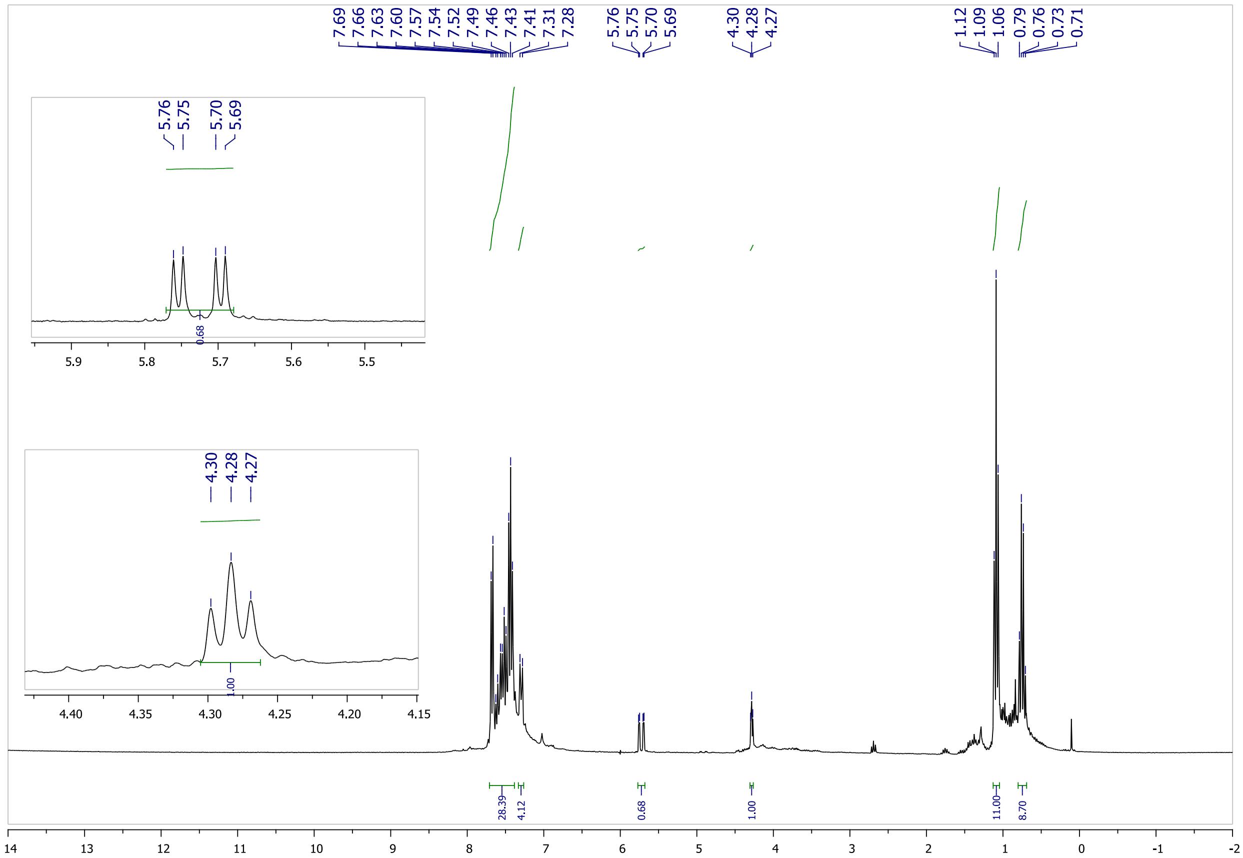

^{31}P NMR of complex **22**

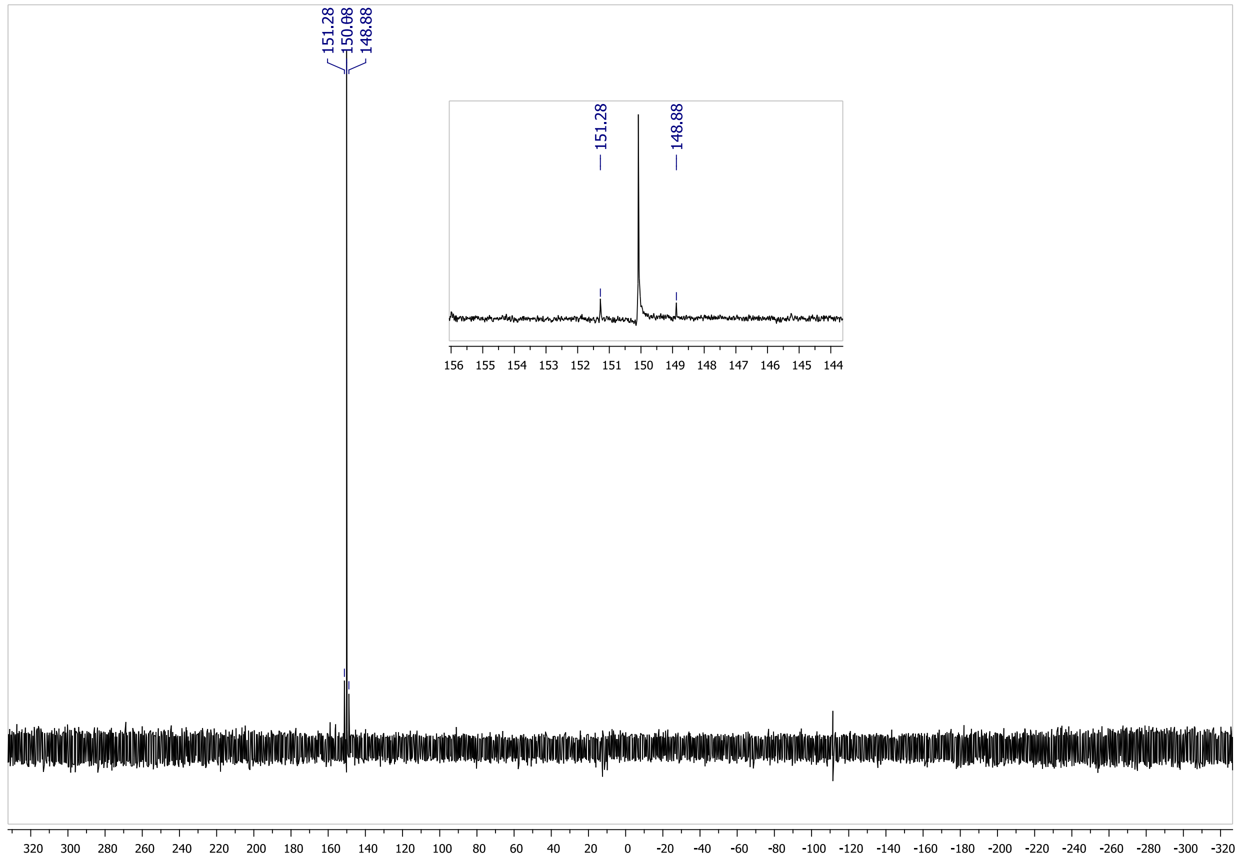

APT - ^{13}C NMR of complex **22**

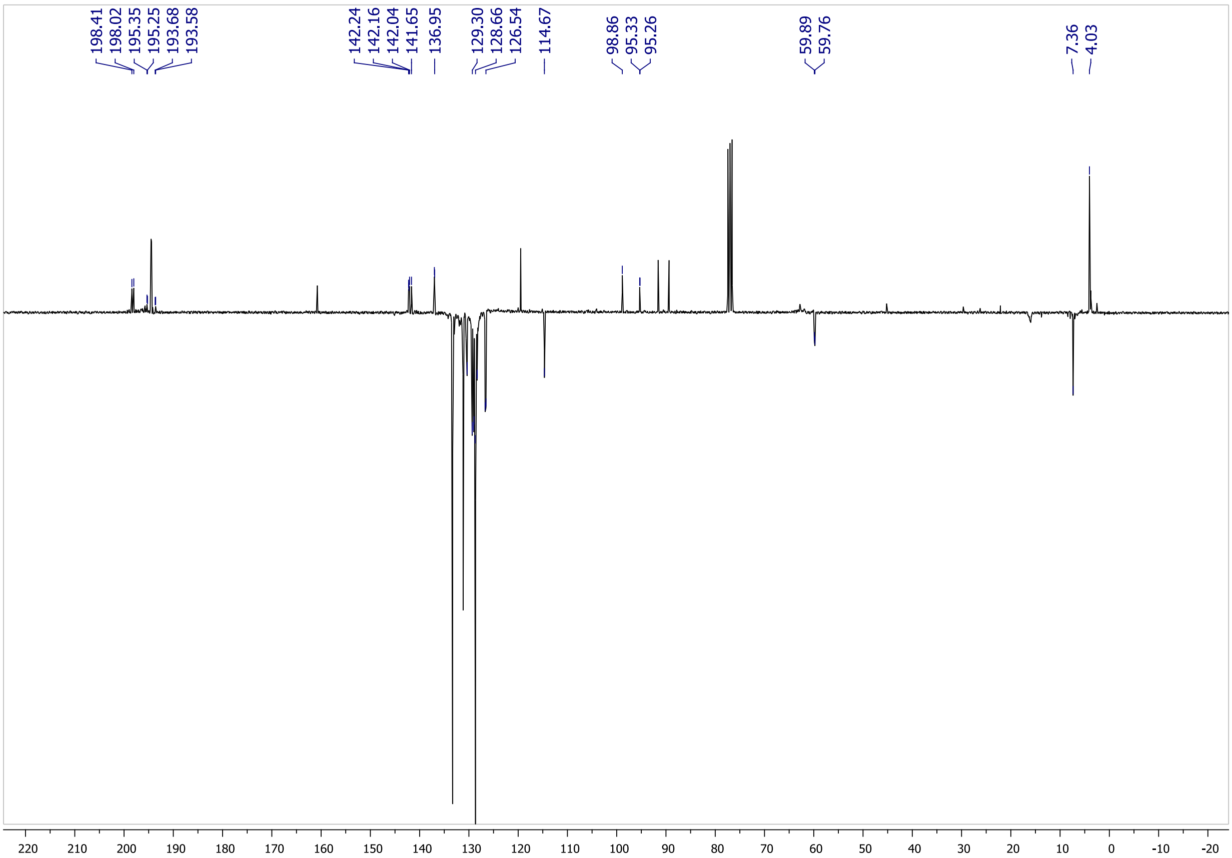

^{1}H NMR of complex **23**

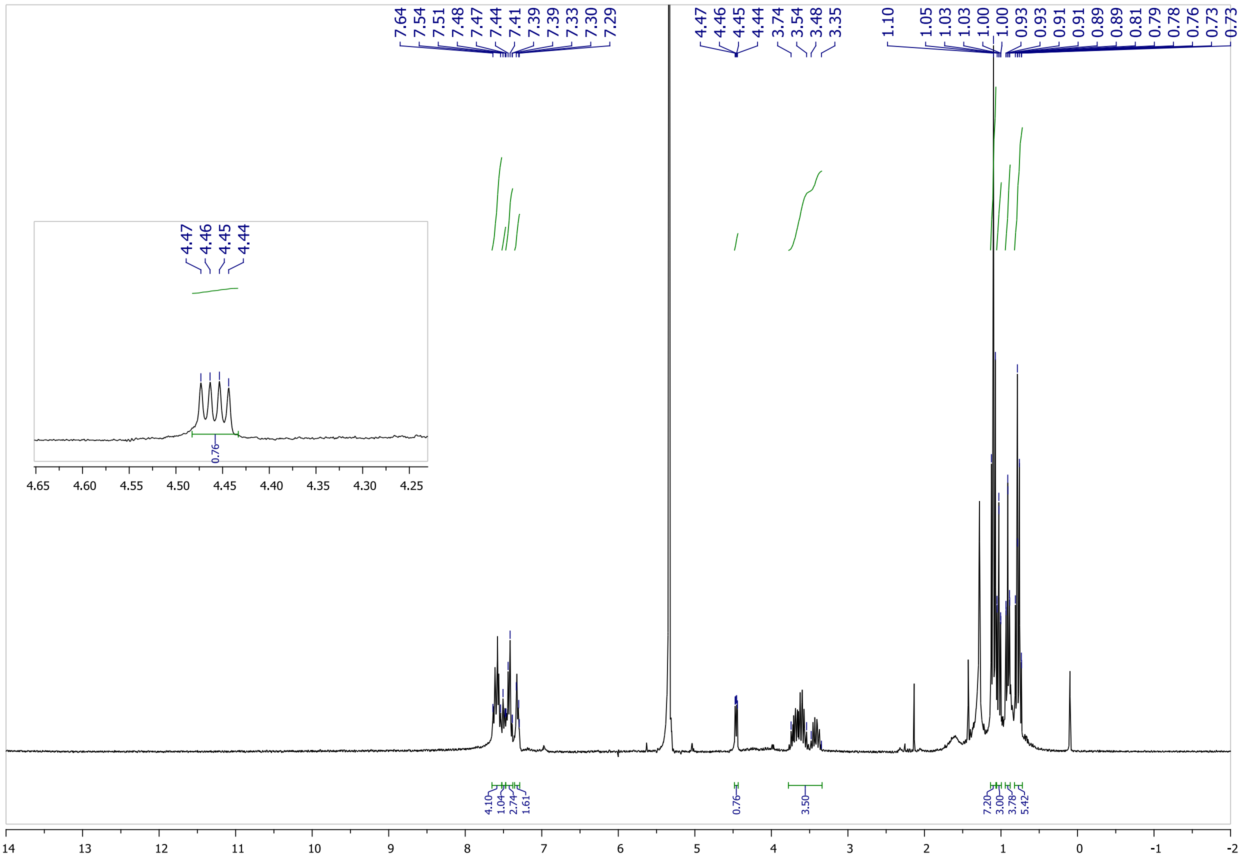

^{31}P NMR of complex **23**

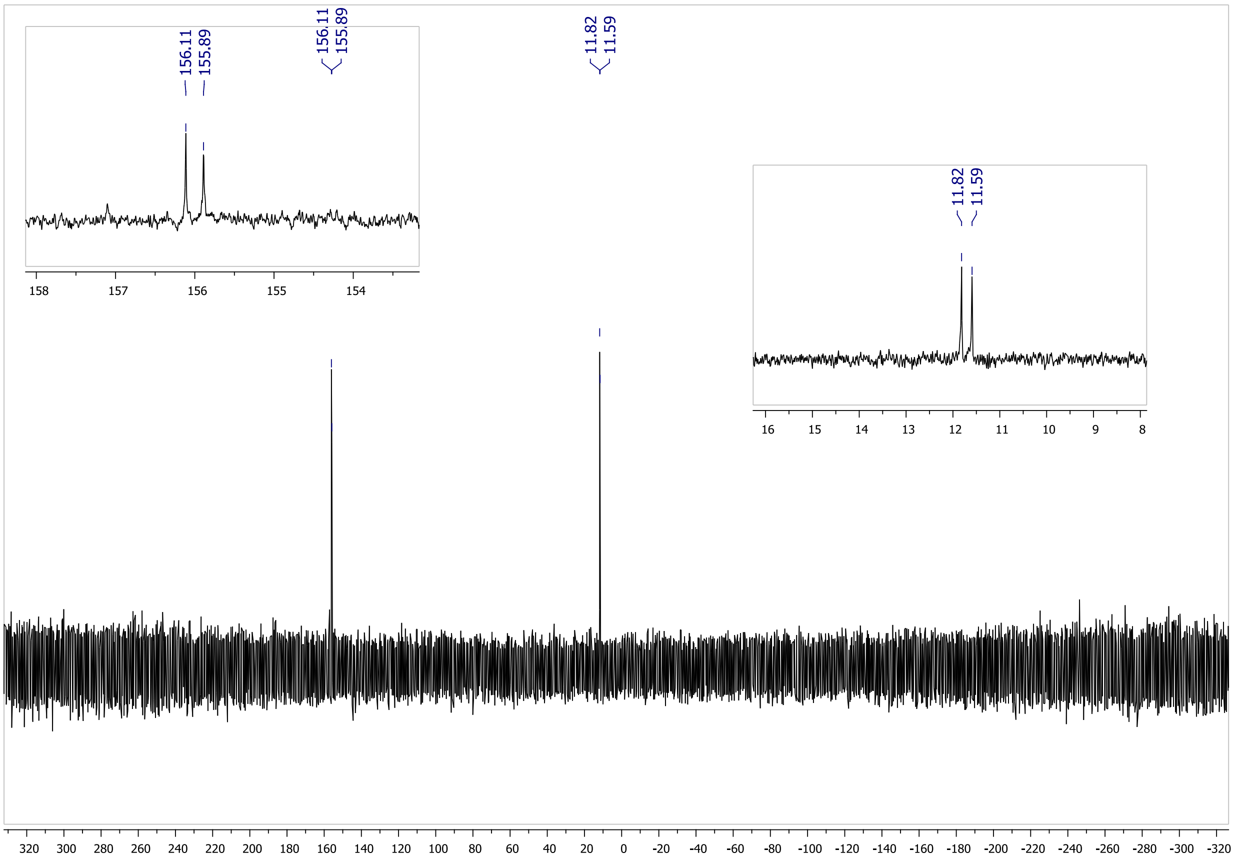

APT - ^{13}C NMR of complex **23**

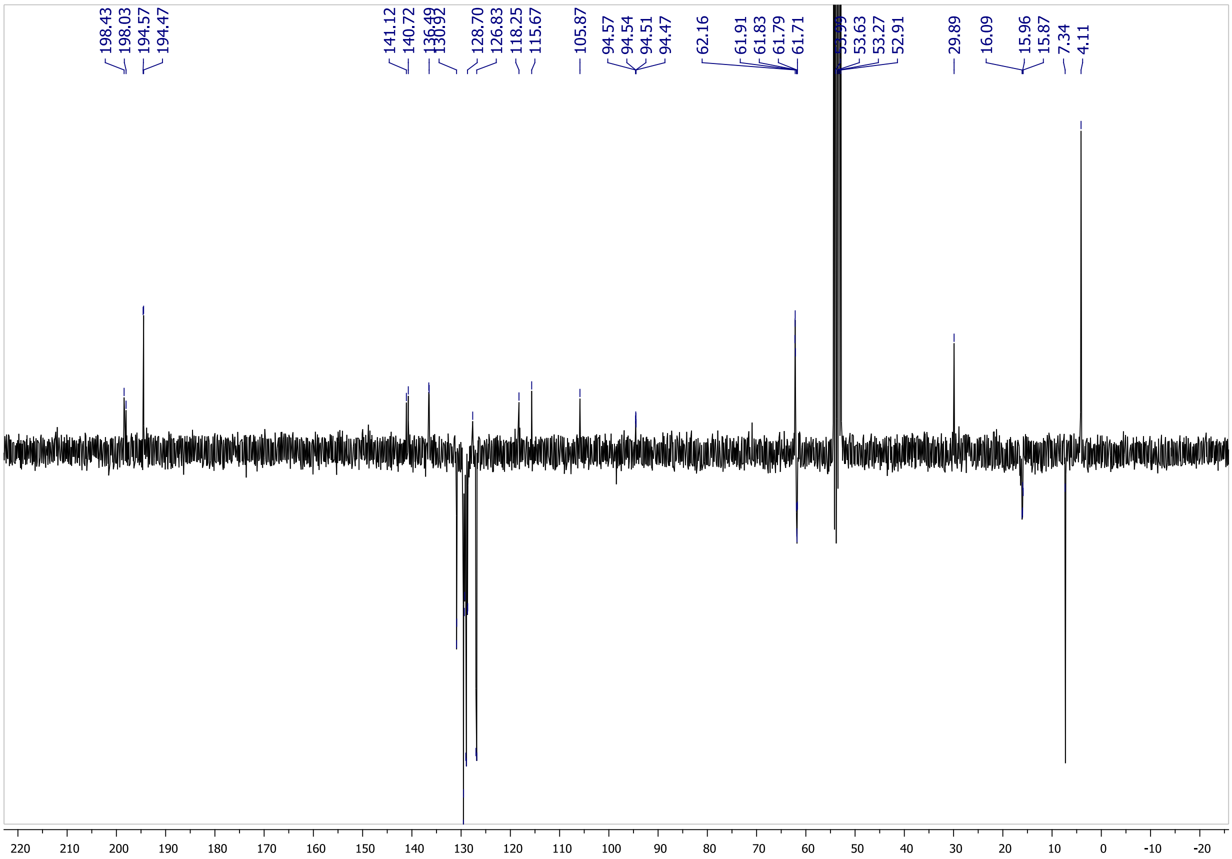

^{31}P NMR of complex **24**

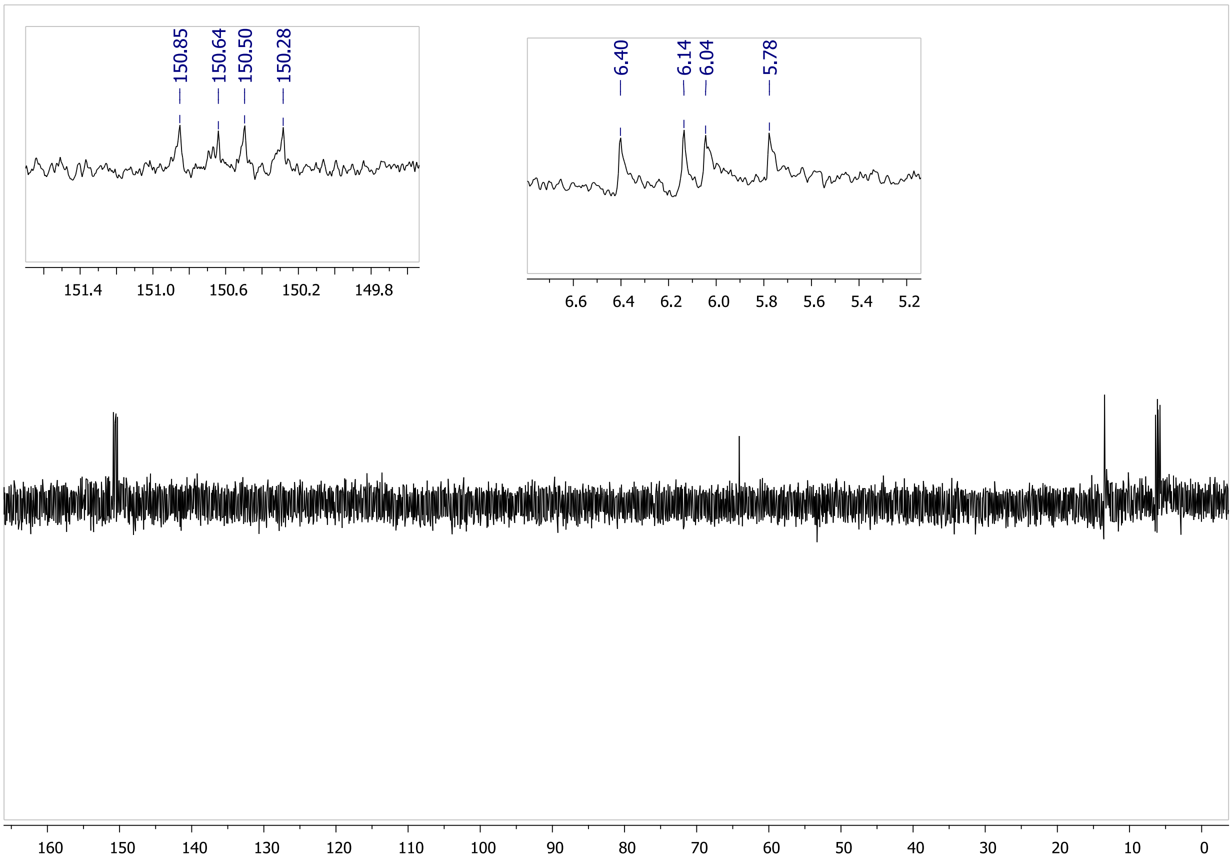

^{1}H NMR of complex **25**

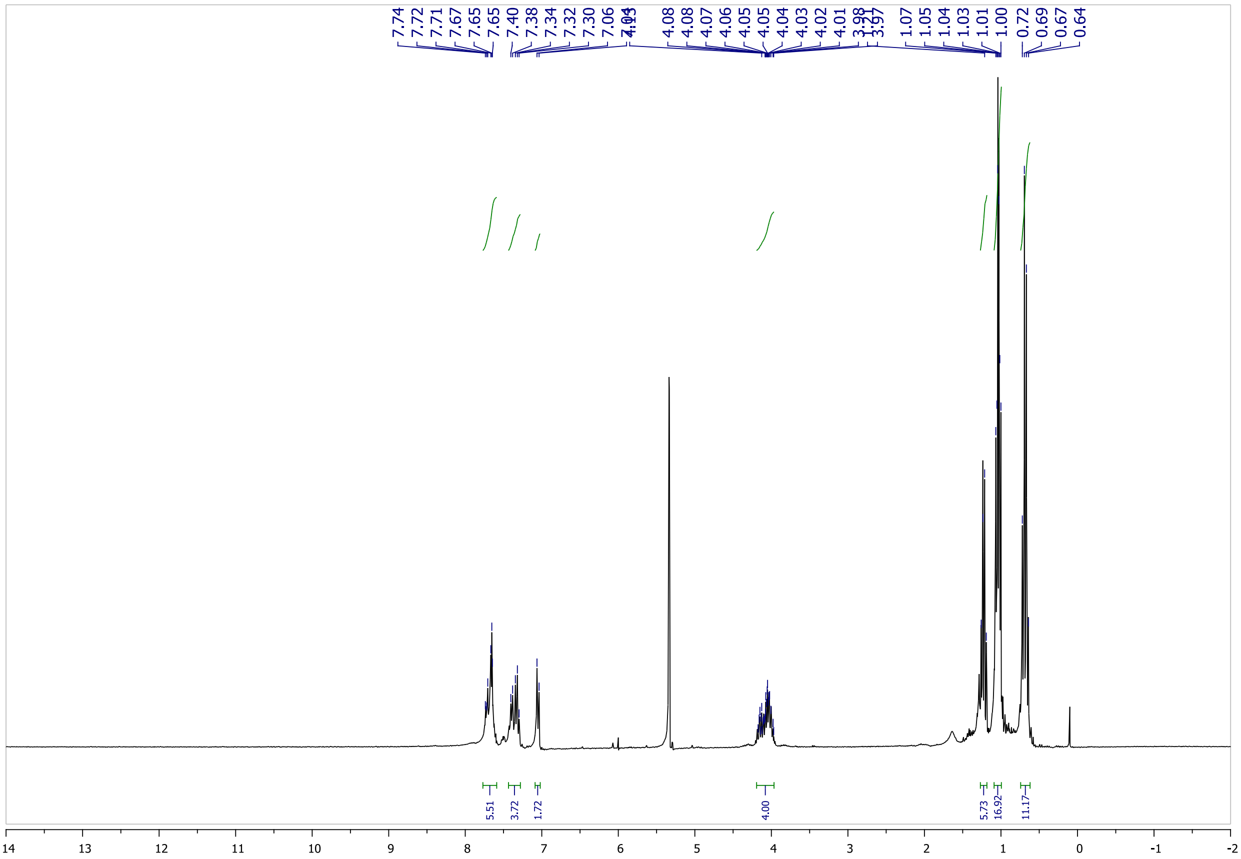

^{31}P NMR of complex **25**

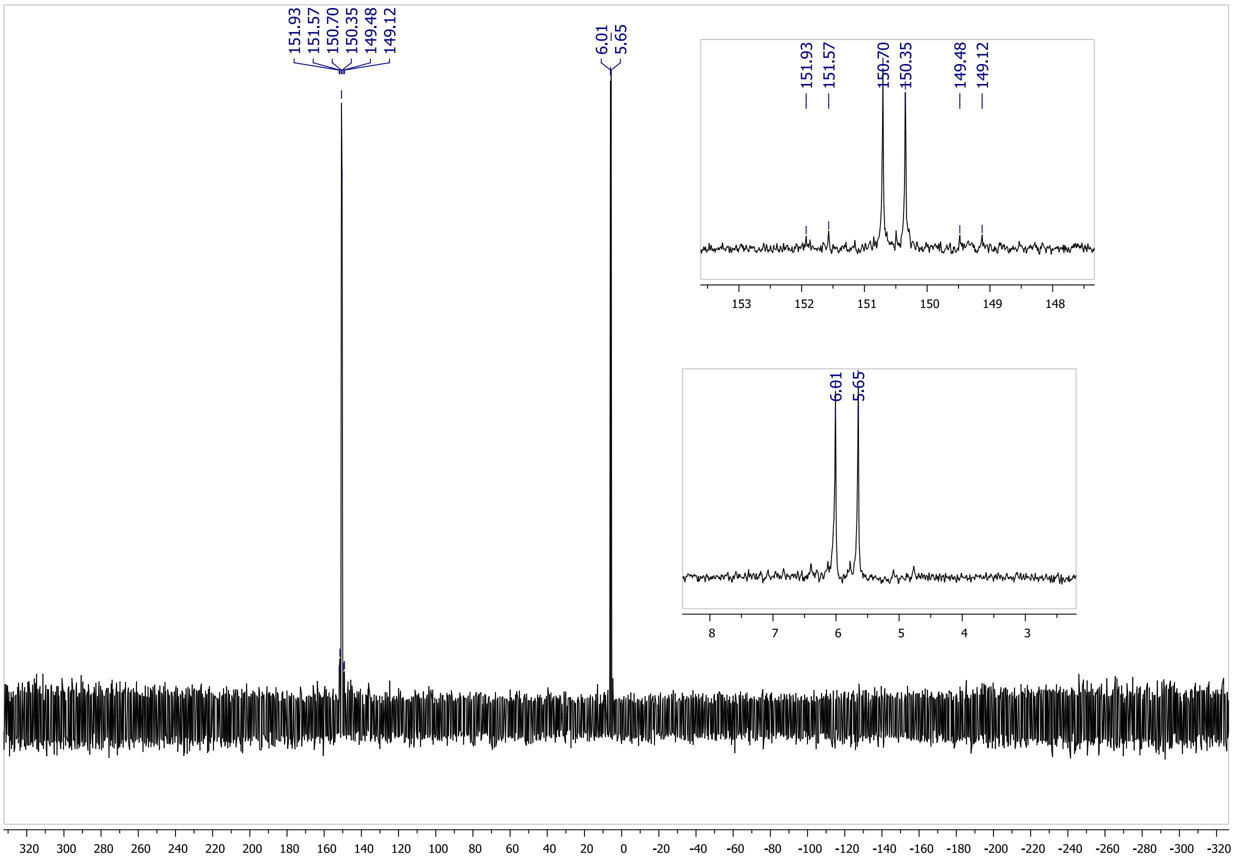

APT-^{13}C NMR of complex **25**

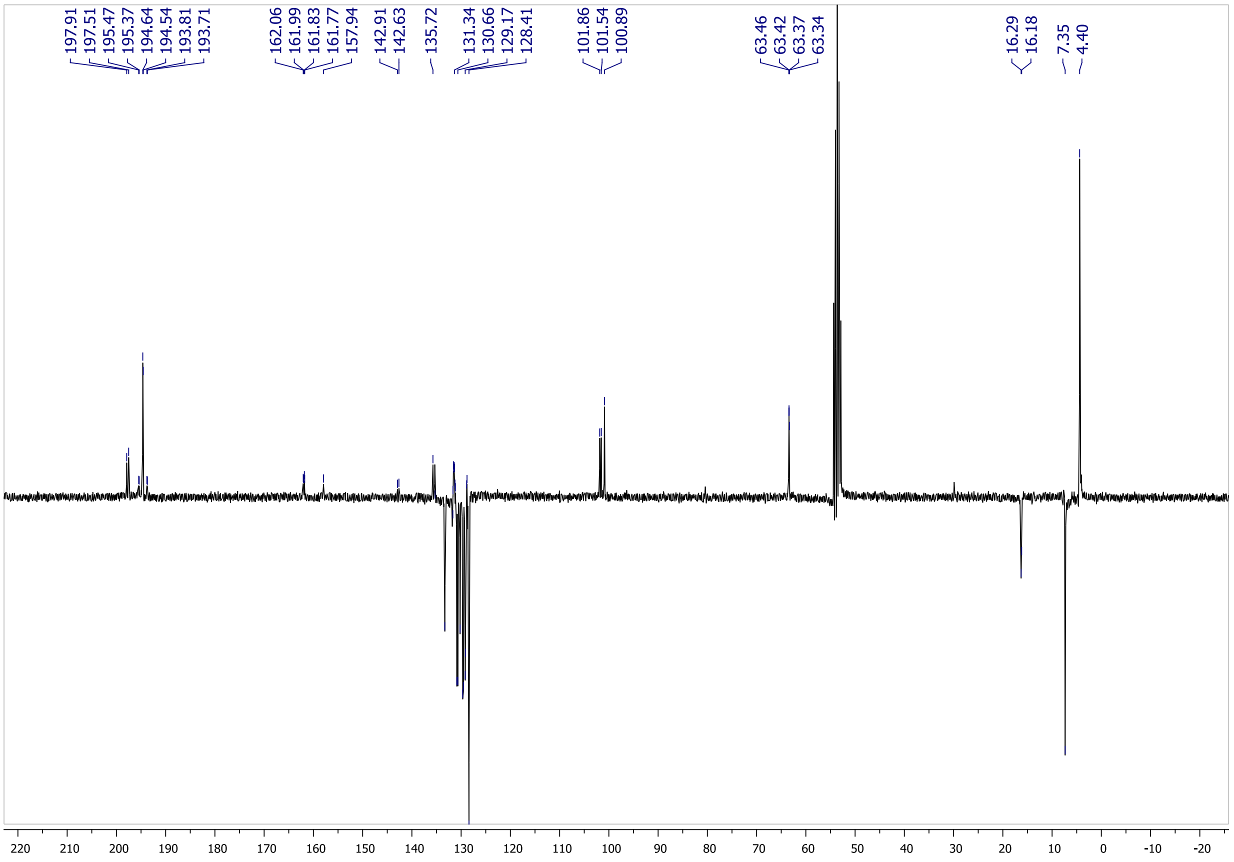

CV data for compounds 15 and 25

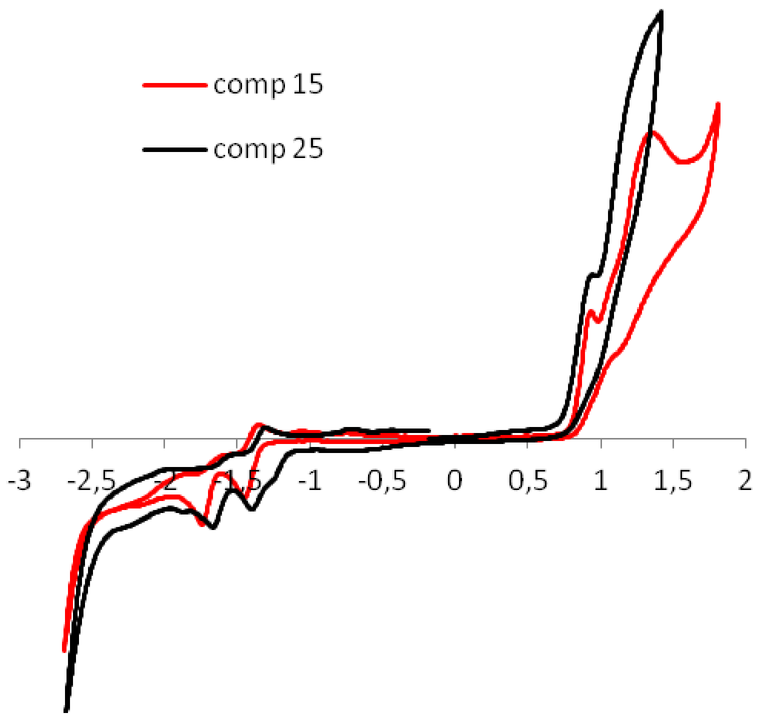

CV data for compounds 16 and 17

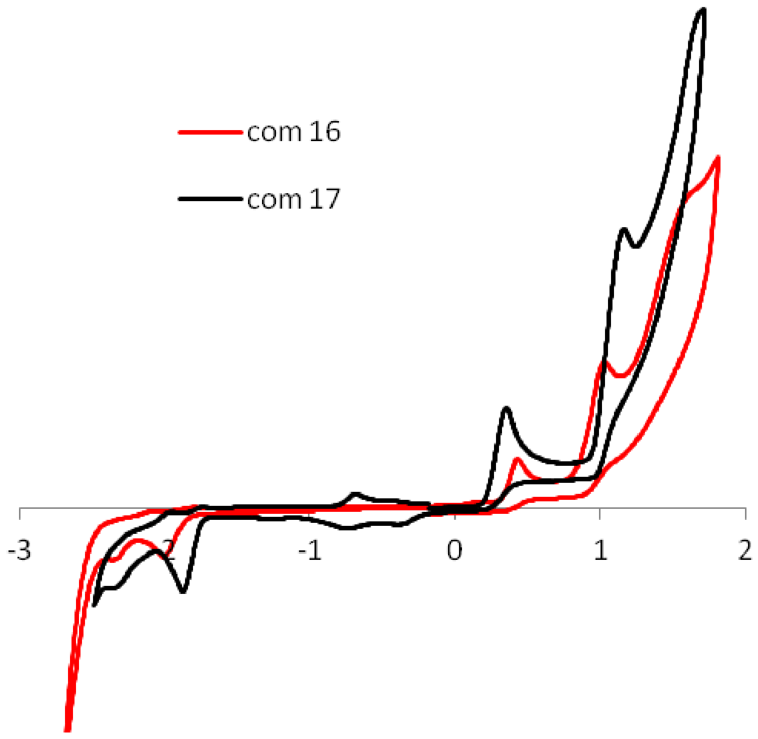

UV/Vis data for compounds 17

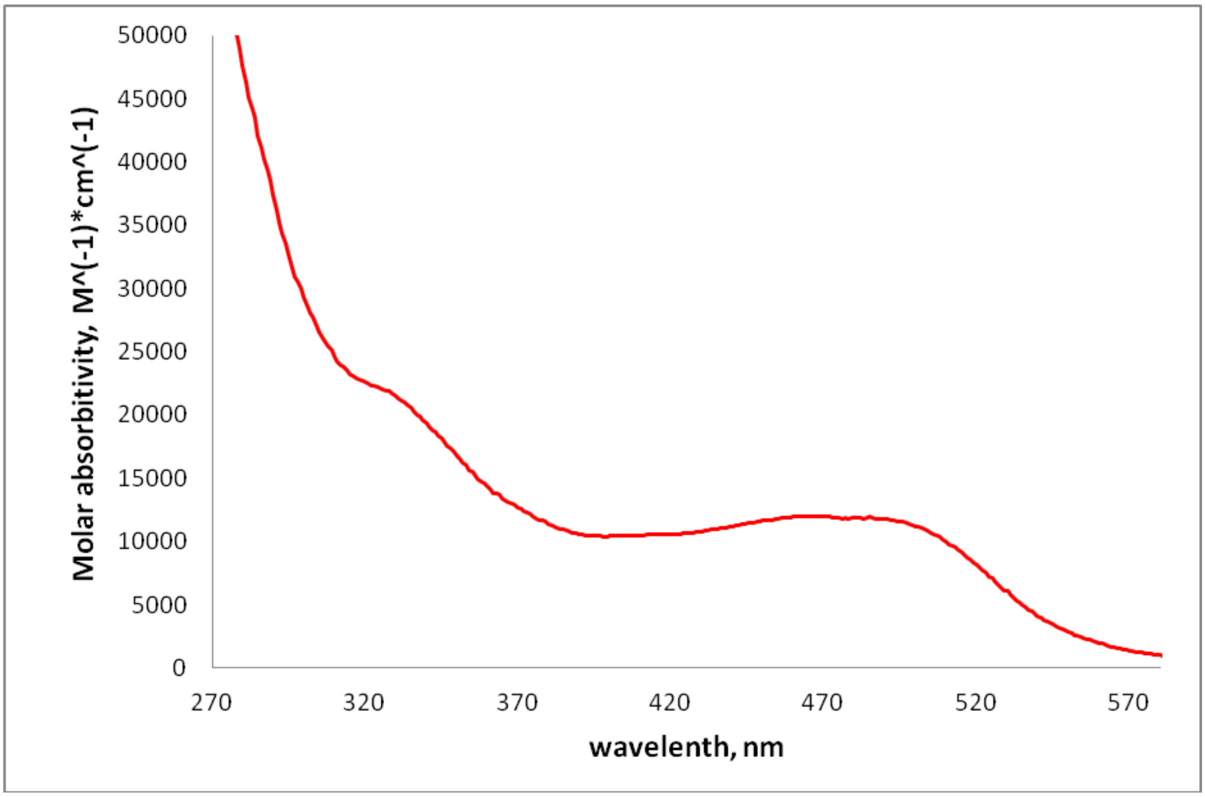

UV/Vis data for compounds 25

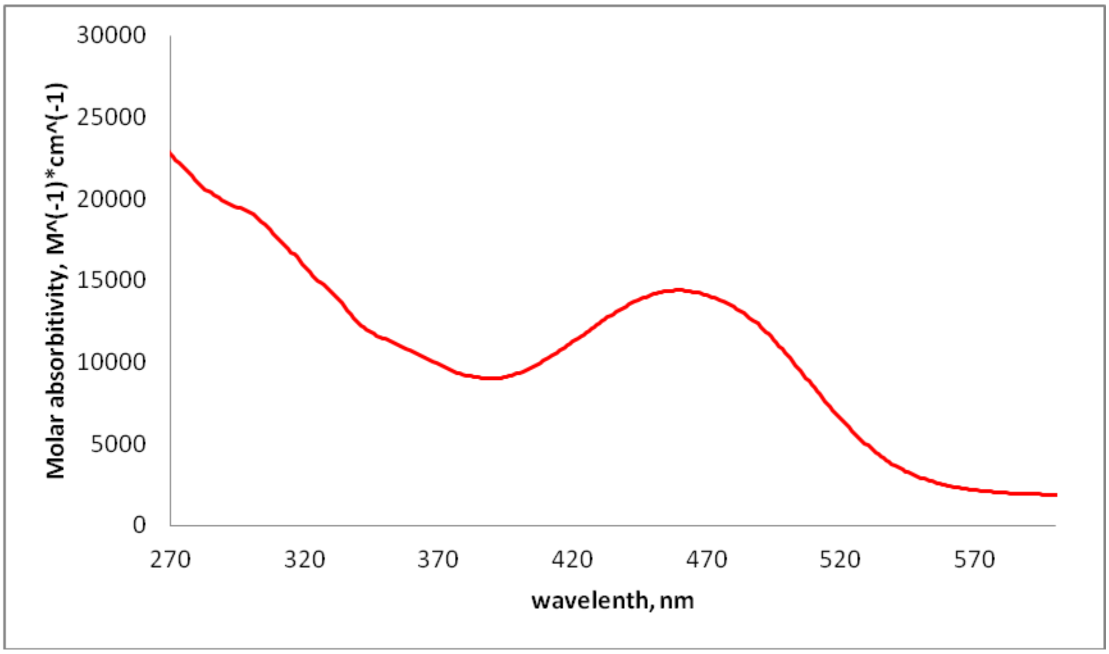

Computational Section

All density functional theory calculations were performed with the Gaussian03 suite of programs.[14] All structures were optimized at the B3LYP level of theory[15] using the 6-31G(d) basis set[16] for the main group elements and the LANL2DZ ECP approximation[17] for chromium.

Geometries are listed as xyz coordinates, electronic energies are given in hartree; for transition states the magnitude of the imaginary frequency is given in cm^{-1}.

C_A - P h

Energy: -2370.89068398
Charge -1, multiplicity 1

C	-0.64587700	0.96479300	0.36503800
C	-2.04461800	0.79447300	0.08835000
C	-3.06351200	1.64610900	0.67546500
C	-2.68511800	2.41940800	1.87214900
C	-2.49488700	3.04840200	2.88953800
H	-2.29508400	3.59185300	3.78423900
O	-4.24900100	1.72501000	0.31529300
P	-2.56870400	-0.44016400	-1.09574600
O	-1.47159700	-1.06710900	-1.89501500
O	-3.50145200	-1.48015200	-0.26485700
O	-3.73288500	0.19325200	-2.05793900
C	-3.44171200	1.34019000	-2.84499900
H	-2.47472600	1.24533500	-3.35503100
H	-4.23373000	1.42096000	-3.59638500
H	-3.45547900	2.23775900	-2.21685200
C	-4.13510600	-2.54875800	-0.96859700
H	-4.47735700	-3.26217100	-0.21413700
H	-4.99188900	-2.17469500	-1.54014600
H	-3.43152500	-3.04641400	-1.64384600
P	0.50078300	-0.32351600	0.64531000
Cr	2.79918400	-0.53970600	-0.32354800
C	2.10082200	-1.60540600	-1.74105600
C	3.02477300	-2.08040100	0.75582300
C	2.49489100	1.00645300	-1.39355500
C	3.49736400	0.52710500	1.07750600
C	4.49187800	-0.73866400	-1.01855800
O	1.76056500	-2.26480300	-2.62563400
O	5.56471300	-0.86647900	-1.45344500
O	3.20134100	-3.01670200	1.41210800
O	2.35383100	1.92485000	-2.07897800
O	3.96868100	1.15827200	1.92532600
C	-0.44854900	-1.84406600	1.06502500
C	-1.19070100	-1.86146100	2.26053800
C	-0.36299900	-3.02876400	0.31590800
C	-1.83064500	-3.02293600	2.69083900
H	-1.26981500	-0.95225400	2.85102700
C	-0.99682500	-4.19404500	0.75104800
H	0.16814300	-3.03329500	-0.62815900
C	-1.73140100	-4.19681100	1.93880300
H	-2.40345900	-3.01250700	3.61602500
H	-0.92198800	-5.09982700	0.15229700
H	-2.22272300	-5.10659000	2.27692500
C	-0.07486800	2.33796300	0.41454900
C	-0.46903800	3.29431000	-0.54149300
C	0.89853900	2.71600600	1.35650400
C	0.09416600	4.56790100	-0.56108200
H	-1.20931600	3.01480800	-1.28164000
C	1.47276800	3.98627700	1.32795100
H	1.18091300	2.00895000	2.12969700
C	1.07401600	4.91920600	0.36964000
H	-0.22305300	5.28425300	-1.31417700
H	2.22796100	4.24701200	2.06408600
H	1.51780800	5.91214000	0.35089100

C_B – Ph

Energy: -2370.89307593
Charge -1, multiplicity 1

C	-0.68168100	-0.46348100	-0.23095900
C	-1.84329400	0.12799200	0.36532200
C	-1.67371800	1.07163800	1.45977800
C	-2.83940300	1.43973700	2.27183300
C	-3.69155200	1.81672200	3.04519400
H	-4.47490200	2.13021400	3.69619300
O	-0.58847100	1.55141700	1.83089000
P	-3.44672600	-0.41975100	-0.19065600
O	-3.48811500	-1.05916800	-1.53825500
O	-4.06057800	-1.32339700	1.02149000
O	-4.48319300	0.84993300	-0.08857500
C	-4.44888400	1.82188800	-1.13450500
H	-4.47472700	1.33748200	-2.11721500
H	-5.33487100	2.45303300	-1.01104500
H	-3.54986600	2.44537300	-1.05669300
C	-5.35853700	-1.89601500	0.85308900
H	-5.49843300	-2.60588300	1.67368000
H	-6.13241000	-1.12079100	0.90482200
H	-5.43129700	-2.42290000	-0.10539200
P	0.79508700	0.40668200	-0.56009300
Cr	3.05696700	-0.27160600	0.28844700
C	2.85333600	0.88892000	1.78574100
C	3.82385300	1.13567600	-0.72011600
C	3.25914900	-1.44380200	-1.19232600
C	2.23553600	-1.64908500	1.31688500
C	4.71209300	-0.76887200	0.92585100
O	2.81345800	1.57467700	2.71424800
O	4.32634400	1.98250400	-1.32850600
O	1.79743000	-2.48319600	1.98405400
O	3.42503400	-2.16002200	-2.08505200
O	5.75909300	-1.08630800	1.32510200
C	-0.65199900	-1.91233800	-0.55535800
C	-1.13721700	-2.85289700	0.37429800
C	-0.10752100	-2.39337500	-1.75900800
C	-1.06670100	-4.21853600	0.11244000
H	-1.55107800	-2.49745300	1.31169600
C	-0.03978300	-3.76126500	-2.02226300
H	0.23312400	-1.67835900	-2.50065000
C	-0.51707100	-4.68054500	-1.08707800
H	-1.42979200	-4.92631600	0.85227700
H	0.37504900	-4.10635000	-2.96531400
H	-0.46669900	-5.74762100	-1.29125200
C	0.36698900	2.17792800	-0.79757900
C	-0.59434400	2.50685900	-1.77311300
C	1.04544200	3.22651800	-0.15795200
C	-0.87443300	3.83696800	-2.08411600
H	-1.12024600	1.70804700	-2.28989300
C	0.77731400	4.55884800	-0.47995700
H	1.77691700	3.00518300	0.61108700
C	-0.18456100	4.87062000	-1.44197500
H	-1.62218900	4.06667500	-2.84028200
H	1.31642900	5.35387500	0.03115000
H	-0.39373400	5.90821800	-1.69256500

C_C - Ph

Energy: -2370.89284024
Charge -1, multiplicity 1

C	-0.82562200	-0.43334200	-0.00828600
C	-1.99504600	0.22718800	0.47294600
C	-1.96497000	1.26995000	1.48774500
C	-0.81276000	1.27691200	2.40564400
C	0.01293900	1.33667500	3.29052800
H	0.78868500	1.36522900	4.02085200
O	-2.87130500	2.07714400	1.73502300
P	-3.56856600	-0.27658700	-0.22448400
O	-3.49023200	-0.89754600	-1.57969400
O	-4.25459100	-1.22471800	0.91696700
O	-4.63435300	0.95439400	-0.16272600
C	-4.40771100	2.09566800	-0.99087900
H	-4.09063100	1.79523000	-1.99765100
H	-5.35997600	2.63075700	-1.06040100
H	-3.65736900	2.74310800	-0.52666200
C	-5.49081200	-1.86593000	0.59599200
H	-5.67856300	-2.60276900	1.38201200
H	-6.30986400	-1.13810200	0.57510100
H	-5.42699100	-2.37065900	-0.37370100
P	0.67403700	0.37848000	-0.41373000
Cr	3.03344400	-0.37419800	0.03279300
C	2.96290800	-1.42647900	-1.55099100
C	2.37570100	-1.83475300	1.06562100
C	3.09720500	0.66764700	1.61207400
C	3.62099200	1.10640000	-0.99568100
C	4.77424200	-0.89921400	0.32256900
O	2.97459400	-2.06124600	-2.51640400
O	2.03212000	-2.72145200	1.71941600
O	5.87620500	-1.22157300	0.51125500
O	4.01667800	1.99240900	-1.62493400
O	3.19789700	1.27966100	2.59015700
C	-0.84481700	-1.89903200	-0.25884900
C	-1.36758100	-2.75862000	0.72624500
C	-0.32256700	-2.47066400	-1.43144400
C	-1.34744900	-4.14067500	0.55028000
H	-1.78182700	-2.32679600	1.63040100
C	-0.30231100	-3.85355800	-1.60628500
H	0.03608000	-1.81620900	-2.21811800
C	-0.81021200	-4.69502700	-0.61434000
H	-1.74589600	-4.78734000	1.32807100
H	0.10037600	-4.27158900	-2.52515100
H	-0.79581000	-5.77406900	-0.75143900
C	0.31321700	2.18104700	-0.56688100
C	-0.56376600	2.60656800	-1.58389900
C	0.97376400	3.15985700	0.19256700
C	-0.77992700	3.96239300	-1.82320000
H	-1.07771000	1.86128300	-2.18732500
C	0.76310100	4.51954600	-0.05236300
H	1.64135100	2.86293400	0.99408100
C	-0.11513000	4.92616900	-1.05759100
H	-1.46400200	4.26823800	-2.61304500
H	1.28049100	5.25996400	0.55472100
H	-0.27950300	5.98417300	-1.24718800

C_A - Si

Energy: -2666.41932764
Charge -1, multiplicity 1

C	-0.97727000	0.42173500	0.21922200
C	-2.01906200	-0.54374600	-0.06403100
C	-3.32684000	-0.57778900	0.54440000
C	-3.45558100	0.08603800	1.85936600
C	-3.64597200	0.53267500	2.97103100
H	-3.81081600	0.93646600	3.94374200
O	-4.31905400	-1.19785100	0.13094500
P	-1.72115600	-1.71927200	-1.38081500
O	-0.32623200	-1.75820000	-1.92331400
O	-2.28214500	-3.14276400	-0.84467300
O	-2.80200600	-1.44542700	-2.59325400
C	-2.47384900	-0.44755500	-3.55688200
H	-1.44518400	-0.56496100	-3.91639900
H	-3.17602700	-0.56840400	-4.38845700
H	-2.59496500	0.56016500	-3.13512900
C	-2.21111200	-4.27318900	-1.71340000
H	-2.47107900	-5.14767300	-1.10961500
H	-2.92461400	-4.17215000	-2.53969700
H	-1.19925700	-4.39418200	-2.11558100
P	0.61890700	-0.18933500	0.54574800
Cr	2.95502200	0.38264200	-0.22271100
C	3.12825700	-1.27804200	-1.13805700
C	3.56487900	-0.41807400	1.38779900
C	2.27724700	1.08517400	-1.85653200
C	2.82857700	2.04840400	0.67172100
C	4.67796700	0.79900000	-0.72587000
O	3.33309000	-2.25032600	-1.72776600
O	5.76395800	1.07272400	-1.04349900
O	3.96298700	-0.88564200	2.36701400
O	1.93408800	1.49084100	-2.88165500
O	2.80143400	3.06738600	1.22147400
C	0.39932400	-1.89211800	1.24361500
C	-0.25475800	-1.99182700	2.48674900
C	0.96067700	-3.05470300	0.69440200
C	-0.34267900	-3.21042400	3.15686100
H	-0.70699600	-1.10563900	2.92299100
C	0.88156500	-4.27411400	1.37253200
H	1.42237000	-3.01818600	-0.28317300
C	0.23414900	-4.35752300	2.60536700
H	-0.85989800	-3.26268500	4.11244000
H	1.32253500	-5.16351000	0.92710600
H	0.17524100	-5.30730700	3.13210100
Si	-1.36084700	2.30488500	0.28190400
C	-0.20677700	3.21084400	-0.94682800
H	0.80260500	2.83821000	-0.74966000
H	-0.44111400	2.88091700	-1.96958800
C	-3.18993200	2.58011500	-0.22556500
H	-3.45689400	1.77379100	-0.92062800
H	-3.82228400	2.41297600	0.65427800
C	-0.99801600	2.96617100	2.04516600
H	0.07813600	3.18554000	2.10641400
H	-1.17607800	2.13704200	2.74019400
C	-3.56274200	3.92804200	-0.86726400
H	-3.01922300	4.09723900	-1.80482100
H	-4.63502100	3.96431600	-1.10379600
H	-3.34746300	4.77729300	-0.20841500
C	-0.16236700	4.74989800	-0.88187400
H	0.64557800	5.14926800	-1.51103100
H	-1.09793400	5.20412700	-1.22563700
H	0.01998500	5.10841800	0.13885100
C	-1.80638400	4.19477300	2.50308400
H	-1.53372300	4.50199800	3.52264500
H	-1.64401500	5.06085600	1.85133400
H	-2.88355500	3.98486200	2.50172000

C_B - Si

Energy: -2666.42286307
Charge -1, multiplicity 1

C	-0.73419000	0.43448800	-0.01065500
C	-1.71248700	-0.48384100	-0.54892000
C	-1.27212000	-1.40853700	-1.58146500
C	-2.26009600	-2.00120600	-2.48383800
C	-2.95747700	-2.55990400	-3.30172200
H	-3.60561800	-3.03828500	-3.99967600
O	-0.08245800	-1.68922600	-1.83608200
P	-3.43195500	-0.42283400	-0.09266200
O	-3.73947100	0.32236300	1.16855400
O	-4.24317200	0.10343600	-1.40435300
O	-4.01759600	-1.95439000	-0.05200200
C	-3.73565000	-2.75363400	1.09961500
H	-4.02765300	-2.23034300	2.01725400
H	-4.32195000	-3.67180200	0.99536500
H	-2.67136800	-3.00542600	1.14869400
C	-5.67093700	0.11430400	-1.37933200
H	-5.99604000	0.65073900	-2.27526400
H	-6.06474000	-0.90816100	-1.40048400
H	-6.04510100	0.63187500	-0.48754500
P	0.83529700	-0.23885900	0.35598000
Cr	3.24275400	0.15318800	-0.38793400
C	3.03910700	-1.28722200	-1.62119000
C	3.74255000	-1.04100300	1.00158800
C	3.50360500	1.59476800	0.81391500
C	2.72387300	1.32371000	-1.79229700
C	5.00719100	0.27844900	-0.88937400
O	3.01813500	-2.13893200	-2.39770700
O	4.10546600	-1.74005600	1.84680000
O	2.48927300	2.01231400	-2.68917500
O	3.74643300	2.45460500	1.54851700
O	6.12718500	0.33422700	-1.20005200
C	0.53941300	-1.95478100	1.00276400
C	1.01564600	-3.14375700	0.42925400
C	-0.10252400	-2.03349200	2.25154900
C	0.85075100	-4.36701100	1.08329200
H	1.48486100	-3.12295800	-0.54443800
C	-0.27054300	-3.25516100	2.90380200
H	-0.47434100	-1.12292800	2.71255200
C	0.20923600	-4.42934100	2.32066200
H	1.22301200	-5.27701800	0.61884600
H	-0.77073700	-3.28546000	3.86861100
H	0.08782600	-5.38282800	2.82888800
Si	-1.08410900	2.27024800	0.44907700
C	-2.62294000	2.90002500	-0.50136900
H	-2.66131700	2.37396900	-1.46479900
H	-3.48581600	2.53010600	0.06243500
C	-1.40584700	2.29421600	2.33562000
H	-2.30450600	1.68209900	2.48133200
H	-0.57626000	1.73995900	2.80029900
C	0.48367900	3.28052800	0.02229100
H	1.29353200	2.84558700	0.61977500
H	0.74032300	3.06931700	-1.02400900
C	0.49957600	4.80914400	0.23693900
H	1.51124900	5.20652800	0.08451800
H	-0.15947000	5.32763600	-0.46758700
H	0.18737600	5.09499300	1.24616700
C	-1.57128400	3.65405000	3.04152200
H	-2.32894000	4.28526900	2.55687200
H	-1.88658100	3.52886300	4.08858800
H	-0.62976300	4.22121200	3.05198300
C	-2.78728900	4.41166500	-0.74549300
H	-2.71650000	4.99698200	0.18020900
H	-2.02956700	4.79914600	-1.43545800
H	-3.76842500	4.63286400	-1.18849200

C_C - Si

Energy: -2666.42319871
Charge -1, multiplicity 1

C	0.80106100	0.34227200	0.19023100
C	1.80491700	-0.55378500	0.68872800
C	1.58928600	-1.51871500	1.75406500
C	0.39315400	-1.36163600	2.60681700
C	-0.47991200	-1.30784300	3.44384600
H	-1.29429000	-1.25632400	4.13192500
O	2.40578100	-2.38280500	2.10090900
P	3.45835700	-0.50088500	0.01800300
O	3.63364300	0.46532700	-1.10932500
O	4.43184100	-0.25518800	1.30495700
O	3.96337000	-1.99920300	-0.41782800
C	3.83771000	-2.40326500	-1.78542100
H	4.10942900	-1.58436300	-2.45956500
H	4.51818200	-3.24883100	-1.92291500
H	2.81408700	-2.73092600	-2.00515300
C	5.84704200	-0.30746000	1.12354100
H	6.29498500	-0.10136300	2.10041600
H	6.15769800	-1.29912600	0.77890700
H	6.17884100	0.45113700	0.40325400
P	-0.76369400	-0.32799200	-0.23656500
Cr	-3.22868600	0.28158800	0.02610500
C	-3.52418500	-1.13996900	1.24340000
C	-3.53392100	-0.88520500	-1.44413000
C	-2.85726300	1.35669800	1.55085300
C	-2.97630800	1.72017800	-1.19433600
C	-5.01893400	0.70276600	0.12458600
O	-3.79421100	-1.97497600	1.99827000
O	-6.14825500	0.97594400	0.18760200
O	-3.74452700	-1.57828600	-2.34385400
O	-2.70922300	1.98822700	2.50630600
O	-2.87704700	2.59130600	-1.94805400
C	-0.52200800	-2.14535100	-0.55749800
C	0.10765000	-2.50263800	-1.76486500
C	-1.05103400	-3.17290200	0.24053500
C	0.21541800	-3.83766000	-2.15904000
H	0.51703400	-1.72119100	-2.40234400
C	-0.94793300	-4.51046200	-0.15495300
H	-1.52301000	-2.93686600	1.18743500
C	-0.31537600	-4.84884500	-1.35101400
H	0.70885200	-4.08762900	-3.09761300
H	-1.36044000	-5.28765100	0.48693100
H	-0.23412600	-5.89014500	-1.65746700
Si	1.18882400	2.19250100	-0.21820200
C	-0.26109000	3.25240600	0.45003000
H	-1.18846900	2.71283800	0.24918200
H	-0.17954300	3.29186000	1.54642800
C	2.78534600	2.75485400	0.68117100
H	2.88227100	2.14017600	1.58666500
H	3.62828600	2.46889900	0.04627000
C	1.27616600	2.38248400	-2.12152200
H	0.24525400	2.46429200	-2.49595800
H	1.68187000	1.44432800	-2.51248000
C	2.89939100	4.24006400	1.07097000
H	2.10492000	4.54991800	1.76026700
H	3.85905900	4.44069000	1.56865800
H	2.84995300	4.89697100	0.19314000
C	-0.41325600	4.67746300	-0.12062100
H	-1.28701100	5.18439100	0.31387700
H	0.45930800	5.30629000	0.08428000
H	-0.56046800	4.65819300	-1.20657200
C	2.12684300	3.55215300	-2.65213400
H	2.11518200	3.58977300	-3.75105400
H	1.76793600	4.52258900	-2.29043200
H	3.17163800	3.44572600	-2.34063100

D-Ph

Energy: -2370.90292476
Charge -2, multiplicity 1

C	-0.83636400	0.44289200	0.21968900
C	-1.85937900	-0.22798900	-0.53428100
C	-1.39701100	-1.24543400	-1.35950700
C	-2.02427100	-2.10314900	-2.27190400
C	-2.48973000	-2.89160400	-3.07448200
H	-2.91621200	-3.50390300	-3.83357900
O	0.00723200	-1.38564700	-1.30771800
P	-3.61558600	0.06757900	-0.28508400
O	-4.03511900	0.42081300	1.09960000
O	-3.99679400	1.15305700	-1.43650500
O	-4.42585200	-1.22197100	-0.87177000
C	-4.54844100	-2.37387900	-0.02832000
H	-4.92753000	-2.08812000	0.95856200
H	-5.25352900	-3.04894100	-0.52162600
H	-3.58146400	-2.87760900	0.07433000
C	-5.32065100	1.69712100	-1.43289300
H	-5.33490900	2.48822500	-2.18737600
H	-6.05352500	0.92625300	-1.69776500
H	-5.56610400	2.11451400	-0.45049000
P	0.66215500	-0.48593900	-0.04765300
Cr	2.80850200	0.44725400	-0.79741200
C	3.20983900	-1.27170600	-1.47372700
C	3.64084200	0.02639600	0.84747900
C	2.43459900	2.19605300	-0.12571500
C	1.87954300	0.87089500	-2.40733600
C	4.40187700	1.07850700	-1.49190500
O	3.50695800	-2.31032700	-1.89210600
O	4.17750300	-0.21563700	1.84462200
O	1.33659400	1.12597300	-3.39309100
O	2.32710900	3.28113900	0.25280200
O	5.40959300	1.46020400	-1.92990000
C	-0.90270300	1.66616000	1.00211300
C	-1.77482400	2.73628000	0.67957800
C	-0.05268300	1.86241000	2.11985500
C	-1.80267900	3.90572900	1.43121200
H	-2.41256600	2.64958500	-0.19364200
C	-0.06760600	3.04574700	2.85585200
H	0.61354300	1.06001000	2.42930800
C	-0.94682900	4.07914600	2.52520200
H	-2.48787900	4.70300300	1.14661600
H	0.60738200	3.15377800	3.70304300
H	-0.96428700	5.00072200	3.10279500
C	0.73837800	-1.78301200	1.31264900
C	-0.26609700	-1.81519900	2.29182600
C	1.76761100	-2.73392200	1.37419000
C	-0.23665200	-2.76912600	3.31150900
H	-1.07003900	-1.08453300	2.24849100
C	1.79595300	-3.69080000	2.38931500
H	2.55142100	-2.73732600	0.62348100
C	0.79557300	-3.70712600	3.36443000
H	-1.02102000	-2.77680200	4.06510300
H	2.60326500	-4.41892900	2.42303100
H	0.82331800	-4.44581100	4.16223200

D-Si

Energy: -2666.45011373
Charge -1, multiplicity 1

C	-0.80263400	0.25978300	-0.04688900
C	-1.69301900	-0.82474500	-0.37880200
C	-1.10603800	-2.05752900	-0.62126100
C	-1.62271100	-3.29053700	-1.03584200
C	-2.01381900	-4.39615600	-1.36251600
H	-2.37561600	-5.32479500	-1.73606300
O	0.30020500	-2.01140300	-0.50973700
P	-3.47690300	-0.67406800	-0.55561600
O	-4.10532200	0.58143200	-0.05089100
O	-3.72997000	-0.97651500	-2.12839100
O	-4.15007800	-2.01249500	0.09289000
C	-4.11255800	-2.16429500	1.51503300
H	-4.57789600	-1.30566600	2.01391700
H	-4.67124700	-3.07522400	1.74624500
H	-3.07806400	-2.27566800	1.86187000
C	-5.07300500	-0.96408900	-2.62242000
H	-5.00405300	-0.94639100	-3.71322600
H	-5.60478600	-1.86753500	-2.30323700
H	-5.61108000	-0.07601600	-2.27291500
P	0.79910800	-0.53381100	0.11277000
Cr	2.97395900	-0.02769200	-0.97650100
C	2.46086500	-1.24521400	-2.35116900
C	3.69736800	-1.47645900	0.01071800
C	3.50692100	1.16343100	0.38985500
C	2.15665900	1.36456100	-1.97185000
C	4.58532900	0.25856300	-1.83583800
O	2.20893800	-1.96790800	-3.21385700
O	4.16833200	-2.35009100	0.60336600
O	1.68614900	2.20328200	-2.61385700
O	3.88961800	1.87404800	1.22135700
O	5.59952200	0.44262400	-2.37492400
C	1.00374400	-1.01674800	1.91484700
C	0.75598600	-2.32905800	2.34404500
C	1.37919100	-0.05801500	2.86732700
C	0.86674300	-2.66715800	3.69600800
H	0.48602400	-3.08468400	1.61376900
C	1.49710800	-0.39665300	4.21619900
H	1.58483900	0.96159700	2.55802500
C	1.23645600	-1.70334100	4.63605800
H	0.66608300	-3.68853500	4.01377500
H	1.79500900	0.35978500	4.93866000
H	1.32535100	-1.96868500	5.68741400
Si	-1.21598500	2.05138700	0.29847900
C	-2.05300200	2.82311000	-1.24819700
H	-1.25174400	3.08449500	-1.95723600
H	-2.62815000	2.02283000	-1.72560700
C	-2.28121900	2.24026500	1.88839200
H	-3.27935500	1.84976900	1.66820400
H	-1.83839300	1.54832100	2.62081500
C	0.40718400	3.02066700	0.66792700
H	0.64029900	2.90308200	1.73707600
H	1.24221800	2.55830400	0.13343800
C	0.38915300	4.52213500	0.32280300
H	1.35066600	5.00020400	0.55695400
H	0.19685300	4.68225800	-0.74448400
H	-0.38627400	5.06275000	0.87873600
C	-2.38918100	3.63711000	2.52848700
H	-2.87494900	4.35738100	1.85977400
H	-2.98155100	3.61053600	3.45468200
H	-1.40583800	4.04789100	2.79047200
C	-2.98528800	4.02571000	-1.03209500
H	-3.80824900	3.76306700	-0.35580900
H	-2.45762800	4.88516600	-0.60101000
H	-3.43733000	4.36333200	-1.97681000

G-Ph

Energy: -2370.88560385
Charge -1, multiplicity 1

C	-0.98932400	0.13787700	0.03968300
C	-1.91222500	-0.80227600	-0.27802400
C	-1.41218800	-2.23351600	-0.38563800
C	0.06154000	-2.32475900	-0.29309500
C	0.76964000	-3.48840100	-0.41330700
H	1.85611900	-3.27108900	-0.30864600
O	-2.19828700	-3.16985200	-0.50409600
P	-3.69651700	-0.47342300	-0.38833700
O	-4.43304700	-0.45154400	0.90871000
O	-3.69055500	0.91712900	-1.22847900
O	-4.35638900	-1.44643400	-1.50159700
C	-5.07789900	-2.62966900	-1.11224000
H	-5.66380500	-2.43714000	-0.20855800
H	-5.74629000	-2.86751900	-1.94521300
H	-4.36734200	-3.43842800	-0.93808900
C	-4.93403800	1.59742900	-1.41865500
H	-4.69601900	2.55463700	-1.89138900
H	-5.59385400	1.01539500	-2.07407200
H	-5.43136500	1.77260800	-0.45805700
P	0.70443700	-0.66140700	0.13828700
Cr	2.74023700	0.15342600	-1.03875900
C	1.76373300	1.06046300	-2.37548200
C	2.68248000	-1.40065400	-2.15882000
C	3.72859000	-0.80091100	0.28329400
C	2.76567600	1.72455300	0.01192500
C	4.33666900	0.65451800	-1.82060200
O	1.21672000	1.63279300	-3.22204200
O	2.70736400	-2.26867700	-2.91786800
O	5.34060800	0.98123600	-2.30801800
O	2.80484300	2.68315200	0.66130500
O	4.38333800	-1.35858200	1.05466800
C	-1.24222200	1.56840600	0.34354100
C	-0.92640200	2.57592100	-0.58384900
C	-1.80625500	1.93808100	1.57792900
C	-1.17642700	3.91585200	-0.28804000
H	-0.51769000	2.30520000	-1.55081400
C	-2.04024100	3.28294100	1.87503200
H	-2.06500700	1.16752800	2.29711400
C	-1.72858800	4.27660300	0.94343400
H	-0.94431600	4.67814800	-1.02829400
H	-2.47574100	3.55066600	2.83482600
H	-1.91526100	5.32333100	1.17205100
C	0.91625700	-0.71579900	1.99217100
C	1.10299600	0.46282000	2.73544000
C	0.91639300	-1.94559600	2.66904000
C	1.27408000	0.41235300	4.11959700
H	1.10844600	1.42777600	2.24087200
C	1.09884200	-1.99262300	4.05263000
H	0.76285200	-2.86154600	2.10801400
C	1.27677500	-0.81643900	4.78314000
H	1.40772100	1.33560300	4.67684600
H	1.09909500	-2.95545600	4.55797000
H	1.41404400	-0.85630700	5.86110600

G-Si

Energy: -2666.40939292
Charge -1, multiplicity 1

C	-0.95247100	0.29124600	-0.18763800
C	-1.85774600	-0.59599000	-0.73077700
C	-1.33959500	-1.60302300	-1.73482500
C	0.12391200	-1.43847200	-1.97767800
C	0.80271000	-1.82606600	-3.05592900
H	1.88557900	-1.64196600	-3.05239800
O	-2.06448200	-2.41890300	-2.29053500
P	-3.59704100	-0.64174200	-0.25302000
O	-3.86253000	-0.01187600	1.07583100
O	-4.42241300	-0.02943500	-1.50904900
O	-4.11003900	-2.17779300	-0.32018500
C	-3.52488500	-3.16220300	0.53901500
H	-3.32865200	-2.75508100	1.53670000
H	-4.24704400	-3.97940600	0.61759300
H	-2.59931700	-3.53536700	0.09256400
C	-5.85398900	-0.04706700	-1.47236900
H	-6.19441400	0.54359700	-2.32629700
H	-6.22463200	-1.07298800	-1.56270300
H	-6.22671800	0.40020200	-0.54429100
P	0.71579200	-0.52268400	-0.41195900
Cr	3.09943400	0.27715100	-0.34937800
C	3.63255100	-1.31699600	-1.26802300
C	3.41723200	-0.66157800	1.26666000
C	2.75368600	1.18633700	-1.98922100
C	2.72575600	1.76890400	0.73877900
C	4.86205300	0.81943500	-0.41715800
O	4.11078700	-2.26582100	-1.72333000
O	5.96784900	1.17612100	-0.46588300
O	3.67141200	-1.20287200	2.25580900
O	2.58970300	1.74766000	-2.98428300
O	2.59741300	2.65345200	1.47809900
C	0.54984400	-1.87980500	0.85859400
C	0.18718300	-1.60975900	2.18765600
C	0.85281000	-3.20381600	0.50309900
C	0.11446000	-2.63594900	3.13116400
H	-0.04709300	-0.59392900	2.48980100
C	0.78847500	-4.22843500	1.45044100
H	1.11700400	-3.43166100	-0.52542100
C	0.41668400	-3.94986500	2.76697300
H	-0.17761700	-2.40539500	4.15316200
H	1.02343100	-5.24788100	1.15306600
H	0.36254600	-4.74877600	3.50284900
Si	-1.27594100	2.08706700	0.41838400
C	-0.08097000	3.15312700	-0.63199200
H	0.82228000	2.57343000	-0.83877400
H	-0.57123800	3.27518700	-1.60933100
C	-3.03944100	2.72080900	0.03306500
H	-3.37826600	2.23847200	-0.89225700
H	-3.71269800	2.34934900	0.81099000
C	-0.94398100	2.13952800	2.29679200
H	0.11820000	1.93086500	2.48029500
H	-1.50525200	1.29268000	2.71520800
C	-3.19510100	4.24536900	-0.12440400
H	-2.60274500	4.63593700	-0.95985000
H	-4.24291400	4.51195100	-0.32051800
H	-2.88822700	4.79013300	0.77630600
C	0.33248300	4.53452800	-0.08873700
H	0.98115800	5.05778600	-0.80439100
H	-0.53022300	5.18204800	0.10115800
H	0.89299000	4.44112800	0.84640000
C	-1.35103400	3.42448800	3.04143300
H	-1.15637100	3.33846400	4.11897700
H	-0.79936100	4.30010200	2.68115200
H	-2.42026200	3.63420300	2.92108800

G’ - Ph

Energy: -1526.13757451
Charge -1, multiplicity 1

C	-0.94675800	-0.32094000	0.07225900
C	0.91831700	1.46765700	-0.36967600
C	1.44694300	0.26841700	-0.02706300
C	0.44474700	-0.81972500	0.24051000
H	1.52461000	2.35849200	-0.51368700
P	-0.87318500	1.39941400	-0.67618600
C	-1.50626100	2.54857100	0.66990200
H	-1.09772900	2.29518100	1.65287400
H	-1.25647200	3.58876700	0.42716700
H	-2.59573500	2.45257000	0.70663700
C	-2.04361000	-1.03747400	0.38183600
C	-3.39843600	-0.67316400	0.15320100
C	-4.04529300	-0.92094500	-1.09522500
C	-4.22368100	-0.13901000	1.18909000
C	-5.39699300	-0.65618400	-1.28085600
H	-3.45474500	-1.33197400	-1.91044800
C	-5.57010300	0.13487600	0.98105300
H	-3.77500800	0.04899100	2.16201800
C	-6.18376600	-0.12220100	-0.25217200
H	-5.84528500	-0.85906400	-2.25367100
H	-6.15627600	0.55329400	1.79954100
H	-7.24088500	0.08274900	-0.40459100
O	0.78811600	-1.96455100	0.54348100
P	3.19876100	-0.02444000	0.23914200
O	3.66676000	-1.24068600	-0.73637800
O	3.83676100	1.23623500	-0.58160700
O	3.69084800	-0.15874100	1.63949500
C	3.75275500	-2.58038200	-0.21445200
H	4.23101800	-3.17834300	-0.99550200
H	4.36373000	-2.59530700	0.69353200
H	2.75113700	-2.95289700	0.00672400
C	5.24923500	1.42843400	-0.49578400
H	5.77936400	0.63298200	-1.03273100
H	5.46679200	2.39125200	-0.96619600
H	5.57939000	1.44305100	0.54832500

G’ - Si

Energy: -1703.75279554
Charge -1, multiplicity 1

C	-2.50945700	0.78112000	-0.24825900
C	-1.18373200	0.92271800	-0.40696400
C	1.26430000	0.57317300	-0.30863600
C	1.24373000	1.92616600	-0.19662000
P	-0.39393400	2.61975400	-0.57123800
C	-0.90712000	3.45558300	1.02488700
H	-0.58309300	4.50154800	1.06082300
H	-2.00212700	3.41383800	1.01773200
H	-0.54943700	2.92348500	1.91191200
C	-0.07864500	-0.07979500	-0.46881600
O	-0.20089700	-1.29668700	-0.64207400
P	2.75744500	-0.42104300	-0.25120800
H	2.13797300	2.50814900	0.01255700
O	3.77260900	0.60547800	0.51663500
O	2.57405700	-1.53876500	0.91756800
O	3.29702900	-0.97379000	-1.52627300
C	2.18169100	-2.88059900	0.57320400
H	1.13736500	-2.88776400	0.25663800
H	2.81649400	-3.26816800	-0.22965100
H	2.31972700	-3.47772000	1.47909800
C	5.13716200	0.20586100	0.64516900
H	5.68608600	1.06992400	1.02985700
H	5.22926700	-0.62779500	1.35150100
H	5.54978100	-0.09351200	-0.32411100
Si	-3.60181400	-0.64278000	0.08601200
C	-4.61902300	-0.96410200	-1.50248800
H	-5.08461900	-0.04043900	-1.86482700
H	-3.97959400	-1.35250500	-2.30478000
H	-5.41372200	-1.70154100	-1.32328300
C	-4.84666400	-0.10272400	1.43909000
H	-5.38761700	0.79981300	1.13192000
H	-5.58177000	-0.89136000	1.65399500
H	-4.33084100	0.12958600	2.38063500
C	-3.00257300	-2.37015900	0.67608600
H	-2.30545700	-2.80099900	-0.04885700
H	-2.46365700	-2.29322300	1.62993600
H	-3.84242400	-3.06640700	0.82466900

K’ - Ph

Energy: -3052.31177760
Charge -2, multiplicity 1

C	0.71108900	1.24789500	0.15815700
C	-0.64600500	1.41213500	0.32989400
C	1.42679600	-0.02183300	0.07052700
C	2.80511200	-0.22063800	0.37449000
C	3.26175000	-1.57028000	-0.10746400
C	2.31847200	-2.33247200	-0.71368100
H	2.49458300	-3.32929700	-1.10619500
C	-1.58270200	0.28961100	0.64114900
C	-3.48686400	-1.16895300	0.52609100
C	-3.22941600	-1.32784800	1.84629800
P	0.67848800	-1.57480100	-0.51032100
C	0.13270100	-1.27049900	-2.27490700
H	-0.09680400	-2.23061500	-2.75359200
H	-0.78986800	-0.67893300	-2.23831500
H	0.90138900	-0.74501600	-2.85200400
P	4.94849100	-2.09423600	0.18388200
O	3.61796900	0.53788300	0.98730000
O	5.07219700	-3.34737300	-0.87878200
O	5.97291700	-1.06428200	-0.56842000
O	5.39903000	-2.46244700	1.56040200
C	6.25706100	-4.12816600	-0.81771700
H	6.08438200	-5.02582400	-1.42130100
H	7.11166500	-3.57579000	-1.23105700
H	6.48638000	-4.41716000	0.21444600
C	6.48674600	0.07214200	0.14852500
H	7.03885800	-0.25896100	1.03617200
H	7.16923100	0.58109700	-0.54086500
H	5.65710900	0.71871100	0.44582000
C	-1.28910000	2.76652800	0.29927900
C	-0.88647600	3.81312600	1.15168600
C	-2.39509800	3.01160600	-0.54351300
C	-1.53397900	5.04987300	1.14885200
H	-0.05548400	3.64610100	1.82961200
C	-3.03950400	4.24780500	-0.54698800
H	-2.72878400	2.20422000	-1.19068300
C	-2.61434800	5.28027000	0.29558900
H	-1.19386900	5.83465800	1.82413300
H	-3.88507200	4.40732100	-1.21595200
H	-3.12103700	6.24483000	0.29134800
C	1.54128000	2.45722900	-0.21590000
C	2.44992700	3.07324800	0.65862700
C	1.41184600	2.99068700	-1.50797000
C	3.17944100	4.19542600	0.26078200
H	2.59645400	2.64866000	1.64340900
C	2.14492900	4.10824800	-1.91176100
H	0.71443300	2.52034600	-2.19581100
C	3.03230800	4.72062200	-1.02491900
H	3.87556200	4.65685900	0.96021200
H	2.01655700	4.50295000	-2.91880900
H	3.60502900	5.59488200	-1.33268100
P	-1.93643900	-0.16020900	2.36061800
C	-0.60171700	-1.33331200	2.97538100
H	-0.40688500	-2.13871500	2.26388000
H	0.32221200	-0.76248600	3.10978300
H	-0.90057700	-1.74626700	3.94831600
C	-2.57773900	-0.22168700	-0.21523400
O	-2.77619400	0.01940300	-1.45704000
P	-4.78999500	-2.03759300	-0.34639200
H	-3.77193500	-1.99557900	2.50742900
O	-5.72076500	-2.55258000	0.91180300
O	-5.84125400	-0.95338300	-0.97461500
O	-4.44632300	-3.13542300	-1.29794000
C	-5.64234000	-0.49207600	-2.32401600
H	-4.65162300	-0.03715500	-2.40689500
H	-5.73611300	-1.32861600	-3.02657200
H	-6.43671600	0.23806300	-2.51333200
C	-6.77732700	-3.45028200	0.60053000
H	-7.16404600	-3.83519400	1.55034700
H	-7.58542800	-2.93291400	0.06635500
H	-6.41946400	-4.28243700	-0.01640300

K’- Si

Energy: -3407.53214971
Charge -2, multiplicity 1

C	0.70944900	1.47685500	-0.07303800
C	-0.64005000	1.61366900	0.21278700
C	1.41148000	0.19465600	-0.13477000
C	2.74322000	0.00439600	0.32066500
C	3.25154300	-1.35351200	-0.08785700
C	2.36960300	-2.12500100	-0.77797200
H	2.58619800	-3.12487500	-1.14097400
C	-1.49392900	0.44473100	0.58740100
C	-3.22543100	-1.21404100	0.41362200
C	-2.76224700	-1.56863600	1.63469200
P	0.71554800	-1.38054800	-0.70735700
C	0.21730300	-1.11920200	-2.49350600
H	-0.01173100	-2.08546400	-2.95936800
H	-0.70165100	-0.52168500	-2.47864200
H	0.98764000	-0.60072900	-3.07492700
P	4.88245800	-1.91466200	0.38358400
O	3.46424300	0.81246300	0.98838100
O	5.14264700	-3.10396000	-0.72672500
O	6.00423600	-0.85086100	-0.15294500
O	5.14621400	-2.36445600	1.78439500
C	6.29819400	-3.91009400	-0.54590200
H	6.21433400	-4.75434100	-1.23882100
H	7.21054500	-3.34405600	-0.77737400
H	6.36292700	-4.28284100	0.48281500
C	6.44153600	0.19075700	0.73806800
H	7.13295600	0.81304700	0.15970400
H	5.57417700	0.76938600	1.06853300
H	6.96708400	-0.24221500	1.59780600

P	-1.26785200	-0.60980300	2.04768000
C	-1.94623200	0.35163500	3.52902100
H	-2.07231900	-0.33378700	4.37797000
H	-1.20830300	1.10729100	3.81829200
H	-2.89978700	0.84674100	3.31785200
C	-2.51863200	-0.05244000	-0.24463000
O	-2.86617600	0.35370900	-1.40495400
P	-4.56942700	-2.06242500	-0.41407800
H	-3.17649600	-2.36368800	2.24620700
O	-5.30873300	-2.79912000	0.86121700
O	-5.74747900	-0.98566500	-0.76934100
O	-4.28507900	-3.00301900	-1.53866500
C	-5.73993000	-0.35479500	-2.06408700
H	-4.78704800	0.16385300	-2.20477300
H	-5.88984800	-1.10486900	-2.84972000
H	-6.57985900	0.34831200	-2.06134100
C	-6.33540900	-3.73240700	0.55446100
H	-6.57486800	-4.26505600	1.48121400
H	-7.23587000	-3.21841300	0.19208300
H	-6.00398000	-4.44757900	-0.20689400
Si	1.76121500	2.87478800	-0.85147400
Si	-1.65327600	3.19698800	0.48541300
C	3.52012200	2.46475100	-1.45299100
H	4.22235300	2.36760900	-0.62537700
H	3.53975600	1.52277100	-2.01143600
H	3.84813100	3.26796100	-2.13086500
C	0.93216900	3.33306400	-2.52235600
H	-0.15763600	3.34069200	-2.48680400
H	1.27950800	4.30480600	-2.90373400
H	1.22604200	2.57048200	-3.25623400
C	2.00954200	4.42878000	0.23656600
H	2.61856200	5.17229300	-0.29783400
H	1.08505700	4.91800000	0.55462700
H	2.56030800	4.13046000	1.13709400
C	-1.58470400	4.66518300	-0.75402600
H	-0.59087400	5.08106000	-0.93718400
H	-2.00890200	4.37175700	-1.72187500
H	-2.21644200	5.47054500	-0.35075300
C	-1.21723400	3.97373700	2.18865200
H	-0.15766700	4.23685500	2.27587900
H	-1.81129500	4.88266600	2.36653200
H	-1.44500900	3.26746400	2.99553800
C	-3.52358300	2.85591700	0.57378400
H	-3.82948200	2.26773700	-0.29619100
H	-3.79823700	2.28868500	1.46897000
H	-4.07998500	3.80507400	0.58501700

Keton

Energy: -266.78698814
Charge +0, multiplicity 1

C	0.00000000	0.51879400	0.00000000
C	1.22374600	-0.26198100	0.00000000
C	-1.22374700	-0.26197900	0.00000000
C	-2.24365500	-0.91164100	0.00000000
H	-3.15002900	-1.47536700	0.00000000
C	2.24365400	-0.91164300	0.00000000
H	3.15003700	-1.47535600	0.00000000
O	0.00000100	1.74017800	0.00000000

Allene'$_{R=TMS}$
Energy: -1057.44807153
Charge -1, multiplicity 1

C	0.361672	-0.207302	0.210069
Si	0.061876	0.139568	2.062100
C	1.541676	0.756317	2.846150
C	2.527178	1.265582	3.413711
C	3.631482	1.810128	4.023960
O	3.794236	1.651616	5.440854
P	5.312988	2.205382	5.942847
C	5.594379	3.064590	4.382158
C	4.622128	2.649549	3.490227
C	-1.434521	1.328519	2.138678
C	-0.519031	-1.486540	2.870781
H	1.132263	-0.975348	0.074939
H	-0.554332	-0.552775	-0.288950
H	0.703473	0.696705	-0.308890
H	-2.324229	0.904428	1.650256
H	-1.697954	1.555217	3.179162
H	-1.202488	2.281384	1.646960
H	-1.453151	-1.851075	2.421161
H	0.239258	-2.270897	2.762780
H	-0.696562	-1.347414	3.944175
H	4.576462	2.953225	2.445475
H	6.388231	3.769161	4.161998
C	6.325707	0.614251	5.690832
H	6.080945	-0.128377	6.462588
H	6.134141	0.186813	4.700233
H	7.394574	0.857552	5.775331

Allene'$_{R=Ph}$
Energy: -879.824497635
Charge -1, multiplicity 1

C	0.129268	0.090952	-0.094609
C	0.136207	-0.132964	1.290061
C	1.374214	-0.293459	1.928898
C	2.565597	-0.233964	1.219705
C	2.581518	-0.007575	-0.191476
C	1.309099	0.153302	-0.822223
C	3.773127	0.052423	-0.913642
C	4.838431	0.104386	-1.546663
C	6.019225	0.148324	-2.236821
H	3.514840	-0.360612	1.733983
H	1.282504	0.327516	-1.894602
H	1.408321	-0.468354	3.004139
H	-0.819224	0.219327	-0.615793
H	-0.792965	-0.180428	1.852824
O	6.003894	0.445486	-3.635414
P	7.536995	0.255585	-4.327866
C	8.329577	0.156733	-2.705953
C	7.339073	0.049209	-1.753104
C	7.447877	-1.578423	-4.809147
H	8.455029	-1.921267	-5.085411
H	7.081850	-2.183020	-3.971988
H	6.788599	-1.717364	-5.676259
H	9.394619	0.171331	-2.504610
H	7.520633	-0.077330	-0.687522

D’$_{R=TMS}$
Energy: -1324.25459267
Charge -1, multiplicity 1

C	-0.635692	0.245174	-0.161322
Si	0.097457	0.227052	1.599765
C	1.885922	-0.024518	1.577143
C	3.106510	-0.217913	1.551912
C	4.468311	-0.468817	1.577165
C	5.145572	-1.699317	1.477428
C	6.521901	-1.605208	1.435989
P	6.968936	0.143200	1.307495
C	7.005728	0.388888	-0.571294
C	-0.753756	-1.169336	2.561403
C	-0.318821	1.895944	2.404159
O	1.269800	-1.519954	5.769338
C	2.051526	-0.591510	5.508619
C	1.705917	0.785632	5.850031
C	1.401488	1.911963	6.179124
C	3.336781	-0.749971	4.914810
C	4.397928	-0.849152	4.319778
O	5.371502	0.624208	1.631629
H	5.401986	-0.962278	3.968549
H	1.140676	2.910901	6.443055
H	-0.433383	-0.699105	-0.681482
H	-1.724891	0.391330	-0.143230
H	-0.198285	1.051908	-0.762136
H	-0.358004	-1.247049	3.580414
H	-1.838705	-1.007725	2.623396
H	-0.588695	-2.136653	2.070782
H	0.160636	2.723176	1.866893
H	-1.402253	2.076869	2.404522
H	0.033593	1.921878	3.441183
H	4.587629	-2.632057	1.497786
H	7.205718	-2.446046	1.432379
H	6.128971	-0.067066	-1.044074
H	7.039750	1.458386	-0.815884
H	7.914212	-0.083625	-0.970094

D’$_{R=Ph}$
Energy: -1146.62573236
Charge -1, multiplicity 1

C	-0.002453	-0.287581	0.149448
C	-0.093290	0.142742	1.479740
C	1.018463	0.779134	2.051778
C	2.184096	0.984351	1.327293
C	2.298271	0.554071	-0.028298
C	1.157564	-0.093827	-0.592257
C	3.465874	0.755455	-0.767034
C	4.508326	0.892242	-1.419807
C	5.655106	1.028323	-2.158725
O	6.765943	1.723643	-1.602875
P	8.133496	1.640135	-2.606266
C	8.983841	0.116846	-1.873365
C	5.897815	0.653319	-3.495150
C	7.171269	0.911181	-3.960972
C	4.630445	-4.509360	-1.534457
C	3.348546	-4.002635	-1.089183
C	2.291626	-3.524449	-0.740953
C	5.436150	-3.513230	-2.199354
C	6.062491	-2.613496	-2.717097
O	4.997606	-5.670018	-1.342690
H	1.408551	-3.015889	-0.417912
H	3.035271	1.479596	1.786507
H	1.209605	-0.425986	-1.625302
H	0.971397	1.122031	3.084101
H	-0.856263	-0.775647	-0.320522
H	-1.003192	-0.011930	2.054126
H	7.499515	0.783292	-4.986725
H	5.102674	0.206778	-4.087987
H	9.466214	0.374204	-0.921805
H	8.272867	-0.698781	-1.702885
H	9.761309	-0.222855	-2.571511
H	6.586265	-1.805005	-3.188626

TS(D’-E’)$_{R=TMS}$
Energy: -1324.24590586
Imaginary frequency: 168 cm^{-1}
Charge -1, multiplicity 1

C	-0.300372	0.272110	-0.307857
Si	-0.021849	0.062830	1.566075
C	1.802987	0.009060	1.942360
C	2.808852	-0.132078	1.167573
C	3.928886	-0.345033	0.458094
C	4.533577	-1.602135	0.128863
C	5.689612	-1.515685	-0.581491
P	6.108206	0.243990	-0.809741
C	7.258227	0.500125	0.659469
C	-0.813734	-1.566251	2.124527
C	-0.833435	1.518401	2.463805
O	0.949589	-0.282404	4.534475
C	2.042529	0.039645	3.970842
C	2.484823	1.449719	4.056741
C	2.793293	2.612700	4.200146
C	3.178788	-0.900529	4.052876
C	4.085025	-1.696503	4.164475
O	4.631369	0.730744	-0.146256
H	4.887724	-2.391835	4.246178
H	3.072236	3.637573	4.279622
H	0.175921	-0.538648	-0.872087
H	-1.370701	0.272688	-0.556904
H	0.128490	1.215574	-0.666464
H	-0.554598	-1.722860	3.176319
H	-1.907276	-1.545806	2.021764
H	-0.434159	-2.414660	1.541391
H	-0.416451	2.476478	2.131423
H	-1.918671	1.540404	2.294524
H	-0.638842	1.410575	3.535083
H	4.062392	-2.527692	0.448041
H	6.268554	-2.360336	-0.938767
H	7.465213	1.568739	0.792803
H	6.816670	0.097822	1.577199
H	8.209931	-0.011516	0.460821

TS(D’-E’)$_{R=Ph}$
Energy: -1146.61513823
Imaginary frequency: 163 cm^{-1}
Charge -1, multiplicity 1

C	0.015027	0.034694	0.011559
C	0.022937	0.058043	1.406454
C	1.240795	0.003973	2.113153
C	2.437320	-0.099279	1.375517
C	2.421550	-0.127056	-0.016952
C	1.208189	-0.056402	-0.708482
C	1.254764	0.006553	3.570021
C	1.895027	0.734612	4.408237
C	2.517351	1.404544	5.381481
O	1.963113	2.586319	5.942642
P	2.821809	3.133494	7.288736
C	1.927002	2.236140	8.680318
C	3.806281	1.125380	5.957372
C	4.172405	1.945986	6.972960
C	0.049778	-1.500742	4.263927
O	-0.913225	-1.683365	3.463469
C	1.132841	-2.513685	4.300765
C	1.967826	-3.392006	4.288169
C	-0.246692	-0.959937	5.607485
C	-0.570724	-0.556670	6.702234
H	2.719743	-4.146246	4.268842
H	-0.901640	0.099839	1.968129
H	3.378169	-0.153666	1.916549
H	-0.934883	0.084068	-0.517668
H	3.359148	-0.199891	-0.565678
H	1.194504	-0.074694	-1.796584
H	5.112128	1.892372	7.512136
H	4.400050	0.299632	5.574788
H	0.977802	2.739442	8.899697
H	1.732100	1.193299	8.408986
H	2.548436	2.266743	9.585642
H	-0.840402	-0.168153	7.656832

F’$_{R=TMS}$
Energy: -1324.255649
Charge -1, multiplicity 1

C	0.015670	-0.009529	0.012472
Si	-0.023150	0.026227	1.977289
C	1.876146	-0.012183	2.264444
C	-0.902555	-1.652200	2.273357
O	-0.292128	0.542990	3.821586
C	-1.088711	1.668620	3.628611
C	-0.493489	2.896167	4.219282
C	0.013865	3.889233	4.688610
C	-1.096905	1.723321	2.074626
C	-1.568857	2.628123	1.278143
C	-1.970565	3.556492	0.420309
C	-2.464114	1.502081	4.169468
C	-3.579998	1.337535	4.606694
O	-3.240766	3.505624	-0.202223
H	0.470360	4.756158	5.106394
H	-4.564990	1.182487	4.981196
H	0.510273	0.881122	-0.403463
H	0.556856	-0.889742	-0.368824
H	-0.997517	-0.035586	-0.414483
H	2.115560	-0.382075	3.267452
H	2.400741	-0.622953	1.516996
H	2.281490	1.007120	2.195696
H	-1.990988	-1.504632	2.277678
H	-0.673158	-2.394274	1.496657
H	-0.634966	-2.061384	3.253993
P	-3.592899	4.891359	-1.101696
C	-1.897417	5.534734	-0.863375
C	-1.220252	4.708125	-0.037894
C	-4.505047	5.930657	0.169796
H	-0.194448	4.846226	0.291052
H	-1.490602	6.430956	-1.319728
H	-5.519501	5.539584	0.308305
H	-3.982264	5.933823	1.131750
H	-4.584019	6.961070	-0.202162

F’$_{R=Ph}$
Energy: -1146.61973343
Charge -1, multiplicity 1

C	-3.177450	-2.670640	0.465465
C	-4.334142	-2.258455	-0.200870
C	-4.347242	-1.015498	-0.838288
C	-3.222906	-0.190141	-0.822828
C	-2.054699	-0.595545	-0.151282
C	-2.053843	-1.846778	0.491269
C	-0.844790	0.271201	-0.099889
C	-1.074797	1.885790	-0.284977
C	0.268062	2.566504	-0.113151
C	1.302225	3.195700	-0.061665
C	0.356119	-0.226139	0.037158
C	1.593779	-0.661626	0.195644
O	-1.649072	2.157978	-1.431521
C	-1.885618	2.277995	0.944106
C	-2.597471	2.620162	1.863923
C	2.294269	-0.885701	1.448276
O	2.430298	-0.979567	-0.902404
H	-3.230183	2.917152	2.667989
H	2.210403	3.751804	-0.030410
H	-3.193423	0.778211	-1.316887
H	-1.156278	-2.165684	1.014851
H	-5.242732	-0.685010	-1.361774
H	-3.150922	-3.634419	0.970968
H	-5.213822	-2.899351	-0.222065
P	4.039750	-1.228291	-0.457760
C	3.599838	-1.200572	1.319141
H	4.278278	-1.398032	2.142604
H	1.763983	-0.771310	2.389307
C	4.737464	0.505907	-0.625474
H	5.691676	0.566196	-0.084877
H	4.926950	0.728062	-1.681749
H	4.039880	1.246118	-0.219975

TS(E’-F’)$_{R=TMS}$
Energy: -1324.255565
Imaginary frequency: 35 cm^{-1}
Charge -1, multiplicity 1

C	0.042203	0.109019	-0.033657
Si	0.061650	0.044313	1.929029
O	0.709933	0.050729	3.719725
C	1.764128	0.960897	3.601522
C	3.048197	0.393254	4.084284
C	4.094059	-0.084963	4.459464
C	1.753438	1.166968	2.064268
C	2.626743	1.735254	1.297455
C	3.517203	2.237254	0.451191
O	3.430828	3.567059	-0.028775
P	4.777007	4.030725	-0.937253
C	5.859007	4.811905	0.385467
C	-0.209343	-1.858900	2.078771
C	-1.530307	1.035763	2.317927
C	4.654731	1.555603	-0.130220
C	5.442086	2.327391	-0.910570
C	1.510093	2.237742	4.318303
C	1.279797	3.272766	4.900463
H	5.008710	-0.516175	4.794024
H	1.065800	4.187575	5.402296
H	0.899425	-0.428717	-0.466325
H	-0.869409	-0.348227	-0.448982
H	0.097875	1.142109	-0.404778
H	-0.536619	-2.136767	3.086932
H	-0.938355	-2.232123	1.346160
H	0.736248	-2.388054	1.892398
H	-1.304014	2.107312	2.392924
H	-2.297910	0.912477	1.542014
H	-1.948599	0.728688	3.283442
H	4.814174	0.502043	0.080081
H	6.321703	1.982665	-1.443840
H	5.466014	5.800539	0.649108
H	5.904021	4.187265	1.283531
H	6.873390	4.943123	-0.014764

TS(E’-F’)$_{R=Ph}$
Energy: -1146.560892
Imaginary frequency: 230 cm^{-1}
Charge -1, multiplicity 1

C	2.013849	1.251488	-0.391764
C	1.333500	1.736579	0.712399
C	0.448621	0.936188	1.452375
C	0.295084	-0.398243	1.046747
C	0.962031	-0.912217	-0.053411
C	1.815102	-0.092866	-0.885498
C	1.513300	-0.234776	-2.629512
C	0.576966	0.023833	-3.467996
C	-0.508747	0.300762	-4.193973
O	-0.673349	1.551799	-4.828783
P	-2.023777	1.602710	-5.847494
C	-1.241153	1.139515	-7.494664
C	2.878033	-0.900990	-2.691787
C	2.840104	-2.307523	-3.134713
C	2.801380	-3.461381	-3.495083
C	3.874921	-0.154177	-3.481560
C	4.694649	0.460784	-4.123353
O	3.119218	-0.790519	-1.275239
C	-1.640208	-0.556544	-4.441526
C	-2.571930	-0.042684	-5.282342
H	2.760639	-4.480742	-3.801374
H	5.408102	1.019614	-4.683079
H	0.837272	-1.956100	-0.333215
H	2.703173	1.891781	-0.937449
H	-0.352293	-1.064394	1.619539
H	1.510005	2.768952	1.018441
H	-0.076434	1.327374	2.320313
H	-3.502678	-0.528811	-5.553867
H	-1.697175	-1.527279	-3.957787
H	-2.037977	0.901326	-8.212099
H	-0.671226	1.989629	-7.887506
H	-0.579133	0.273486	-7.391687

F’$_{R=TMS}$
Energy: -1324.266771
Charge -1, multiplicity 1

C	4.904754	0.754674	-1.074902
P	4.479180	-0.946083	-0.367967
C	4.243322	-0.520358	1.386628
C	2.918269	-0.270346	1.594786
C	2.073102	-0.452078	0.459316
O	2.817246	-0.998478	-0.637147
C	0.753665	-0.206780	0.338193
C	-0.508496	-0.057756	0.295460
C	-1.350652	1.147217	-0.041114
C	-0.898134	1.814281	-1.282798
C	-0.481495	2.346739	-2.284661
O	-2.739590	0.774887	-0.233391
Si	-3.236483	-0.833795	-0.084623
C	-1.305744	2.116828	1.077439
C	-1.275265	2.896581	2.000772
H	-0.107681	2.791175	-3.177133
H	2.490172	0.048632	2.542706
H	5.018958	-0.458873	2.143262
H	4.900399	0.719341	-2.171676
H	4.191067	1.510564	-0.732797
H	5.912220	1.038705	-0.744351
C	-5.099868	-0.623903	-0.434136
C	-3.135642	-1.588818	1.650452
C	-2.578710	-2.005549	-1.420120
H	-2.125284	-1.933051	1.881432
H	-3.421811	-0.845129	2.405163
H	-3.833844	-2.434377	1.741229
H	-1.506131	-2.185000	-1.313251
H	-3.107258	-2.968710	-1.378334
H	-2.756752	-1.574400	-2.413693
H	-5.607845	-1.598900	-0.443255
H	-5.583377	-0.003888	0.331259
H	-5.277538	-0.145044	-1.404553
H	-1.238117	3.587665	2.810636

F’$_{R=Ph}$
Energy: -1146.61973
Charge -1, multiplicity 1

C	-0.122991	0.138075	-0.373963
C	-0.328883	0.275108	0.997409
C	0.762277	0.327178	1.868455
C	2.055655	0.241424	1.347105
C	2.268115	0.112163	-0.025478
C	1.174997	0.055468	-0.909647
C	1.367239	-0.101875	-2.377993
C	0.491161	0.342684	-3.240181
C	-0.367891	0.742790	-4.161118
O	-0.299768	2.028196	-4.752250
P	-1.308687	2.192991	-6.095488
C	-0.159574	1.663386	-7.481377
C	2.763741	-0.801558	-2.877485
C	2.670797	-2.223353	-2.336776
C	2.674356	-3.336463	-1.856174
C	2.695865	-0.943121	-4.384935
C	2.764919	-1.041903	-5.590623
O	3.809592	-0.125841	-2.466674
C	-1.480018	-0.011032	-4.713359
C	-2.112399	0.585789	-5.744893
H	2.680208	-4.311976	-1.427673
H	2.839290	-1.120260	-6.650823
H	3.260043	0.054078	-0.467445
H	-0.972388	0.092264	-1.050720
H	2.913020	0.282794	2.016804
H	-1.343789	0.336681	1.386635
H	0.604690	0.433317	2.940403
H	-2.958639	0.170366	-6.282114
H	-1.724170	-0.987238	-4.304419
H	-0.753630	1.426975	-8.374415
H	0.522375	2.484334	-7.730430
H	0.424360	0.785135	-7.187312

TMS-O$^-$
-484.455119
Charge -1, multiplicity 1

C	-0.036213	-0.010418	0.068962
Si	-0.024400	0.154920	1.997937
C	1.859471	0.000180	2.419052
C	-0.720396	-1.559322	2.568417
O	-0.757265	1.420285	2.582556
H	0.420306	0.874631	-0.399475
H	0.510186	-0.896752	-0.292887
H	-1.066627	-0.078925	-0.311158
H	2.012655	-0.053286	3.506263
H	2.328789	-0.891514	1.975050
H	2.414668	0.878650	2.059899
H	-1.784779	-1.652143	2.308331
H	-0.193523	-2.414533	2.116000
H	-0.646912	-1.666345	3.660252

Phenyl O$^-$
Energy: -306.884156
Charge -1, multiplicity 1

C	0.006487	0.008531	-0.015526
C	-0.055405	0.008594	1.435856
C	1.252433	0.004148	2.065985
C	2.436638	-0.000174	1.340278
C	2.444388	-0.001550	-0.064289
C	1.201585	0.004623	-0.721050
O	-1.143353	0.006827	2.083227
H	-0.950859	0.013494	-0.538085
H	1.272697	0.007993	3.156701
H	1.176071	0.007219	-1.814946
H	3.389265	-0.002982	1.877982
H	3.378623	-0.006334	-0.623649

Cumulene’
Energy: -839.724528800
Charge +0, multiplicity 1

C	0.045792	0.127719	0.028416
P	0.216683	0.037823	1.883116
O	1.921219	-0.108870	2.011999
C	2.337508	-1.387540	2.286718
C	1.231215	-2.320358	2.384352
C	0.025685	-1.754059	2.165555
C	3.639798	-1.667940	2.457073
C	4.850601	-1.938643	2.617951
C	6.158087	-2.239643	2.787641
C	6.558067	-3.571905	3.105713
C	6.910020	-4.699273	3.373415
C	7.155791	-1.229748	2.646912
C	8.016696	-0.385551	2.532794
H	7.218488	-5.692215	3.609964
H	8.764581	0.367352	2.429173
H	1.410053	-3.364904	2.616779
H	-0.922114	-2.278677	2.219285
H	0.379688	1.112807	-0.312273
H	0.624720	-0.651930	-0.475386
H	-1.014813	0.020533	-0.229443

References

1. Verkruijsse, H.D., Y.A. Heus-Kloos, and L. Brandsma, *Efficient methods for the preparation of acetylenic ketones.* Journal of Organometallic Chemistry, 1988. **338**(3): p. 289-294.
2. Mukherjee, S., et al., *Improved Method for the Synthesis of β-Carbonyl Silyl-1,3-Dithianes by the Double Conjugate Addition of 1,3-Dithiol to Propargylic Carbonyl Compounds.* The Journal of Organic Chemistry, 2009. **74**(23): p. 9206-9209.
3. Taylor, C.J., M. Motevalli, and C.J. Richards, *Synthesis of Planar Chiral Cobalt Metallocenes by Microwave-Assisted Diastereoselective Complexation.* Organometallics, 2006. **25**(11): p. 2899-2902.
4. Schuler, M., et al., *Gold(I)-Catalyzed Alkoxyhalogenation of β-Hydroxy-α,α-Difluoroynones.* Angewandte Chemie International Edition, 2008. **47**(41): p. 7927-7930.
5. She, Z., et al., *Synthesis of Trisubstituted Isoxazoles by Palladium(II)-Catalyzed Cascade Cyclization–Alkenylation of 2-Alkyn-1-one O-Methyl Oximes.* The Journal of Organic Chemistry, 2012. **77**(7): p. 3627-3633.
6. Chunhai Zou, C.L., Yannick Coppel and Remi Chauvin, *Ring carbo-mers: From questionable homoaromaticity to bench aromaticity.* Pure Appl. Chem., 2006. **78**(4): p. 791-811.
7. Klein, O., H. Hopf, and J. Grunenberg, *The Ever-Elusive Tetra-tert-butylethene (TTBE, 3,4-Di-tert-butyl- 2,2,5,5-tetramethylhex-3-ene): Further Insight on Its Preparation.* European Journal of Organic Chemistry, 2009. **2009**(13): p. 2141-2148.
8. Marshall, J.A., P. Eidam, and H.S. Eidam, *(R)- and (S)-4-TIPS-3-butyn-2-ol. Useful Precursors of Chiral Allenylzinc and Indium Reagents.* The Journal of Organic Chemistry, 2006. **71**(13): p. 4840-4844.
9. Lange, T., et al., *Towards the Synthesis of Tetraethynylallene.* Synthesis, 1996. **1996**(04): p. 537-550.
10. , E., et al., *C,C-Diacetylenic Phosphaalkenes as Heavy Diethynylethene Analogues.* The Journal of Organic Chemistry, 2009. **74**(24): p. 9265-9273.
11. Bowling, N.P., et al., *Synthesis of Simple Diynals, Diynones, Their Hydrazones, and Diazo Compounds: Precursors to a Family of Dialkynyl Carbenes (R1—C≡ — —C≡C—R2).* The Journal of Organic Chemistry, 2010. **75**(19): p. 6382-6390.
12. Eisler, S., et al., *Alkyne Migration in Alkylidene Carbenoid Species: A New Method of Polyyne Synthesis.* Chemistry – A European Journal, 2003. **9**(11): p. 2542-2550.
13. Auffrant, A., et al., *Synthesis of 1,4-Diethynyl- and 1,1,4,4-Tetraethynylbutatrienes.* Helvetica Chimica Acta, 2004. **87**(12): p. 3085-3105.

14. Gaussian 09, Revision A.1, M. J. Frisch, G. W. Trucks, H. B. Schlegel, G. E. Scuseria, M. A. Robb, J. R. Cheeseman, G. Scalmani, V. Barone, B. Mennucci, G. A. Petersson, H. Nakatsuji, M. Caricato, X. Li, H. P. Hratchian, A. F. Izmaylov, J. Bloino, G. Zheng, J. L. Sonnenberg, M. Hada, M. Ehara, K. Toyota, R. Fukuda, J. Hasegawa, M. Ishida, T. Nakajima, Y. Honda, O. Kitao, H. Nakai, T. Vreven, J. A. Montgomery, Jr., J. E. Peralta, F. Ogliaro, M. Bearpark, J. J. Heyd, E. Brothers, K. N. Kudin, V. N. Staroverov, R. Kobayashi, J. Normand, K. Raghavachari, A. Rendell, J. C. Burant, S. S. Iyengar, J. Tomasi, M. Cossi, N. Rega, J. M. Millam, M. Klene, J. E. Knox, J. B. Cross, V. Bakken, C. Adamo, J. Jaramillo, R. Gomperts, R. E. Stratmann, O. Yazyev, A. J. Austin, R. Cammi, C. Pomelli, J. W. Ochterski, R. L. Martin, K. Morokuma, V. G. Zakrzewski, G. A. Voth, P. Salvador, J. J. Dannenberg, S. Dapprich, A. D. Daniels, Ö. Farkas, J. B. Foresman, J. V. Ortiz, J. Cioslowski, and D. J. Fox, Gaussian, Inc., Wallingford CT, 2009.

15. A. D. Becke, *Phys.Rev. A* 1988, **38**, 3098–3100. b) A. D. Becke, *J. Chem. Phys.* 1993, 98, 5648–5652. c) C. Lee, W. Yang, R. G. Parr, *Phys. Rev. B* 1988, **37**, 785–789.

16. R. Ditchfield, W. J. Hehre, and J. A. Pople, *J. Chem. Phys.*, **54** (1971) 724. b) W. J. Hehre, R. Ditchfield, and J. A. Pople, *J. Chem. Phys.*, **56** (1972) 2257. c) M. J. Frisch, J. A. Pople, and J. S. Binkley, *J. Chem. Phys.*, **80** (1984) 3265-69. d) P. C. Hariharan and J. A. Pople*Theor. Chem. Acc.*, **28** (1973) 213-22.

17. P. J. Hay, W. R. Wadt *J. Chem. Phys*. **82** (1985) 299. b) L. E. Roy, P. J. Hay, R. L. Martin *J. Chem. Theory Comput*. **4** (2008),1029. c) P.J. Hay, W. R. Wadt *J. Chem. Phys*. **82** (1985) , 270. d) W. R. Wadt, P.J. Hay *J. Chem. Phys*. **82** (1985), 284.
